# 16p13.11 deletion/duplication: a large cohort study on prenatal diagnosis, postnatal outcomes, and phenotypic manifestations

**DOI:** 10.1186/s12884-025-08467-2

**Published:** 2025-12-23

**Authors:** Xianglian Tang, Jiasun Su, Wei Li, Chaofan Zhou, Yuan Wei, Weiliang Lu, Linlin Wang, Jiao Li, Shujie Zhang, Fei Chen, Yueyun Lan, Sheng He, Zailong Qin, Shengkai Wei, Liang Wang, Peng Huang, Jingsi Luo

**Affiliations:** 1 Genetic and Metabolic Central Laboratory, Guangxi Birth Defects Research and Prevention Institute, Maternal and Child Health Hospital of Guangxi Zhuang Autonomous Region, Nanning, 530003 P.R. China; 2 Guangxi Clinical Research Center for Birth Defects, Guangxi Clinical Research Center for Pediatric Diseases, Guangxi Key Laboratory of Reproductive Health and Birth Defects Prevention, Guangxi Key Laboratory of Precision Medicine for Genetic Diseases, Guangxi Key Laboratory of Birth Defects and Stem Cell Biobank, Guangxi Key Laboratory of Birth Defects Research and Prevention, Maternal and Child Health Hospital of Guangxi Zhuang Autonomous Region, Nanning, China; 3 Guangzhou Women and Children’s Medical Center, Guangzhou Medical University, Guangzhou, Guangdong China; 4 Prenatal Diagnosis Center, Maternal and Child Health Hospital of Guangxi Zhuang Autonomous Region, Nanning, China

**Keywords:** 16p13.11 deletion/duplication, Prenatal diagnosis, Postnatal outcome follow-up, Genetic counsel, Variable expressivity, Incomplete penetrance

## Abstract

**Objectives:**

To expand the clinical phenotype spectrum and improve the understanding of prenatal ultrasound manifestations and fetal prognosis of 16p13.11 deletion/duplication syndrome in the East Asian population.

**Methods:**

We conducted a comprehensive ultrasound phenotypic analysis, pedigree analysis and long-term postnatal outcome follow-up on 201 fetuses with 16p13.11 deletion/duplication, as well as on the phenotypic manifestations of 14 patients who underwent chromosomal microarray analysis between April 2013 and July 2024. Descriptive statistical analysis was used.

**Results:**

The detection rates were 0.08% and 0.18%, the frequencies of de novo occurrence were 26.9% and 14.5%, and the rates of abnormal postnatal phenotypes were 25% and 17.5% in our prenatal cohort of deletion and duplication, respectively. Overall, 28.6% of deletions and 15.9% of duplications exhibited abnormal postnatal phenotypes even if they were inherited from a phenotypically normal parent. Developmental delay was the most common clinical abnormality. Immune disorders, torticollis, concealed penis and cryptorchidism were closely related phenotypes that had previously gone unnoticed. Copy number variations extending to intervals I + II or II + III appeared to be associated with a broader range of phenotypes. Isolated choroid plexus cysts may be the most relevant ultrasound soft marker for deletion, whereas isolated thickened nuchal translucency appears to be more closely associated with duplication. Cardiovascular and urinary malformations were the most frequently detected ultrasound structural abnormalities.

**Conclusion:**

The large East Asian prenatal cohort is conducive to enhancing genetic counseling for 16p13.11 deletion/duplication syndrome by facilitating a more accurate prediction of fetal prognosis and developmental potential.

**Supplementary Information:**

The online version contains supplementary material available at 10.1186/s12884-025-08467-2.

## Introduction

Deletion (Del)/duplication (Dup) in the 16p13.11 recurrent region is associated with a range of neurodevelopmental disorders, including developmental delay (DD), intellectual disability (ID), mental retardation (MR), autism spectrum disorder (ASD), attention-deficit hyperactivity disorder (ADHD), behavioural problems, epilepsy, schizophrenia, and multiple congenital abnormalities and deformative features [1–10].

The pathogenicity of copy number variations (CNVs) in the 16p13.11 region remains debatable because of variable phenotypes and low penetrance. The evidence supporting haploinsufficiency and triplosensitivity for the 16p13.11 recurrent region (BP2-BP3) is emerging, and this region scores a 2 in the ClinGen database (https://search.clinicalgenome.org/kb/gene-dosage/region/ISCA-37415). In the Database of Genomic Variants, the incidences of 16p13.11 Del and Dup in the general population are approximately 0.03% and 0.14%, respectively. Moreover, the limited fetal phenotypes present considerable challenges for variant interpretation and genetic counseling.

Herein, we retrospectively reviewed 201 fetuses and 14 patients with 16p13.11 Del/Dup and summarized the clinical details, ultrasound manifestations, results of chromosomal microarray analysis (CMA), pregnancy outcomes and follow-up information to comprehensively understand the fetal phenotypes and the wide clinical spectrum, thereby dissecting genotype‒phenotype correlations and facilitating the precise assessment of fetal prognosis during genetic counseling.

## Materials and methods

### Subjects and ethics approval

From April 2013 to July 2024, a total of 79,136 prenatal and 4,895 peripheral blood samples underwent CMA at the Maternal and Child Health Hospital of Guangxi Zhuang Autonomous Region, from which data on 16p13.11 Del/Dup were collected. This study was approved by the Medical Ethics Committee of Maternal and Child Health, Guangxi Zhuang Autonomous Region, and signed informed consent was obtained from each patient (File NO. 2023–7–18, 17 July 2023).

Chorionic villus sampling (CVS), amniocentesis or cordocentesis was performed under ultrasound guidance, and peripheral blood samples were collected following the receipt of informed consent and payment from the participating families. This study involved a review and analysis of prenatal examinations, pregnancy outcomes and postnatal evaluations. Descriptive statistical analysis was used.

### Chromosomal microarray analysis

Genomic DNA was extracted from the amniotic fluid, chorionic villi or peripheral blood using a TIANamp Micro DNA Kit (DP316, TIANGEN Biotech, China), and the umbilical cord blood was extracted using a Lab-Aid DNA kit (824 s, Zeesan, China) in accordance with the manufacturer’s protocol. DNA quality was assessed by agarose gel electrophoresis, and the DNA concentration was measured with a Nanodrop spectrophotometer (Thermo, USA).

CMA was performed using an Illumina HumanCytoSNP-12 v2.1 BeadChip (Illumina, San Diego, CA, USA). The SNP data were collected and analyzed using Illumina Genome Studio and KaryoStudio software, with coordinates presented according to the human (GRCh37/hg19) assembly.

### Data collection and follow-up of outcomes

The collected data included sample types, prenatal diagnosis indications, pregnancy outcomes (termination of pregnancy, eutocia or cesarean), delivery date, gestational age at delivery (preterm or full-term), fetal sex, birth weight/length, head/chest circumference, newborn assessment, and pediatrician-evaluated physical examination and developmental details.

Pregnancy outcomes and postnatal assessments, as evaluated by obstetricians and neonatologists, were obtained from the Gui Women and Children’s Health Service Information Management System and supplemented by telephone interviews conducted by the hospital’s clinical follow-up center. The follow-up strategy adhered to the standardized health management protocol for children aged 0–6 years, as outlined in the National Basic Public Health Service Standards. Children underwent neurodevelopmental evaluations conducted by pediatricians that assessed gross motor, cognitive, language, fine motor/visuo-perceptual, and behavioral domains, using the General Movements Assessment, the Gesell Developmental Schedule, and the Wechsler Intelligence Scale. The age range of surviving children who could be followed up was from 1 month to 7 years.

## Results

**Fig. 1:**
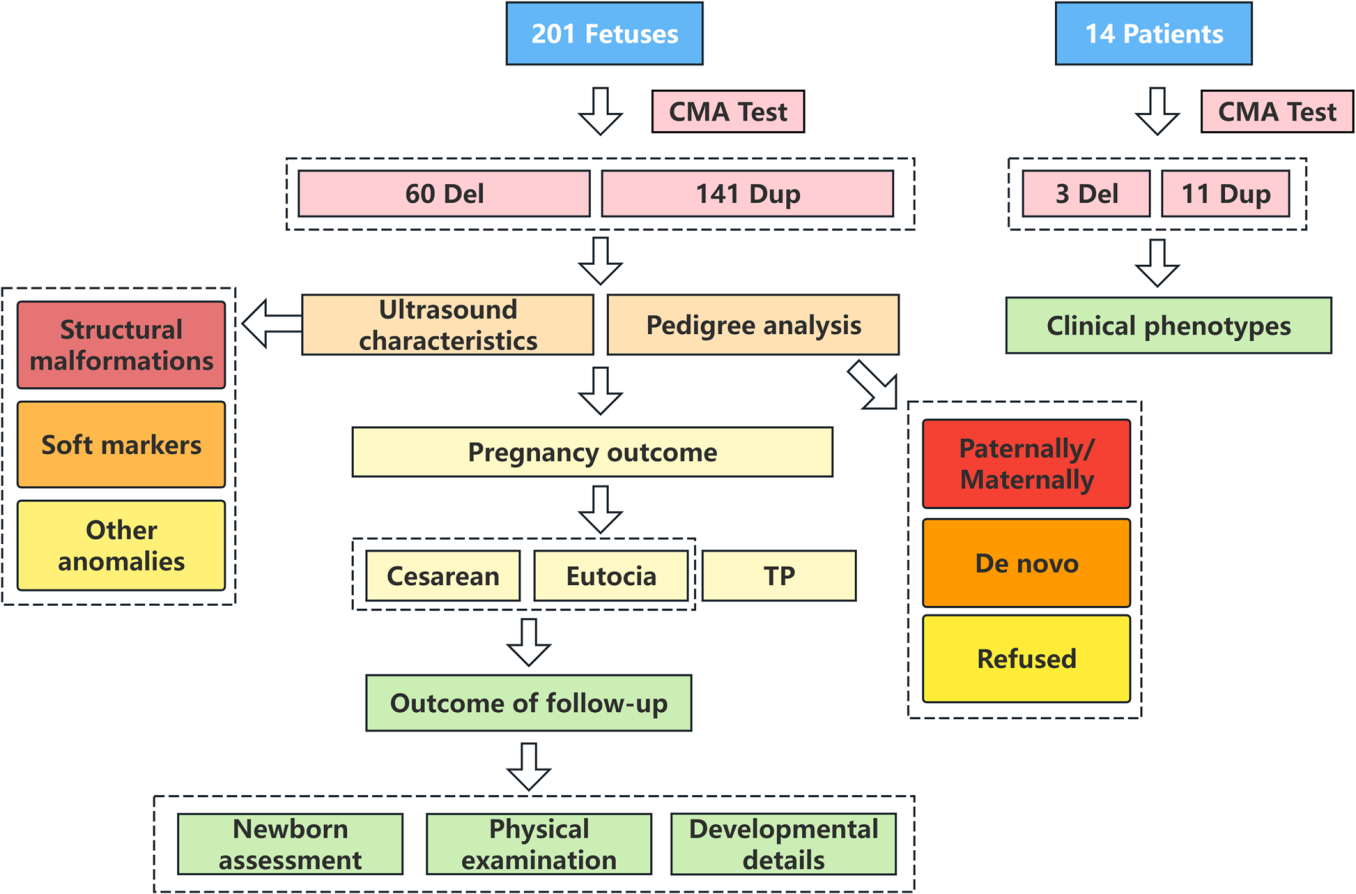
Data flowchart of our prenatal and postnatal cohorts with deletion and duplication

We identified 201 fetuses, including 60 Del and 141 Dup fetuses, in the 16p13.12–p12.2 region between 14.62 and 22.67 Mb (Human Genome Build 37). The detection rates of 16p13.11 Del and Dup were 0.08% (60/79,136) and 0.18% (141/79,136), respectively. The CMA results, clinical information and follow-up outcomes are provided in Tables S1–S2 and summarized in Table 1. Fetuses with abnormal ultrasound characteristics are available in Table 2, along with the corresponding outcome follow-up data provided in Tables S3 and S4. Fetuses with abnormal postnatal phenotypes are listed in Table 3. Furthermore, we detected 91 peripheral blood samples, including 22 Del and 69 Dup in the 16p13.12–p12.3 region between 14.76 and 19.03 Mb (Human Genome Build 37) (Table S5). The data of 14 patients with clinical abnormalities are shown in Table 4. Unless otherwise noted, no additional genomic abnormalities with clinical relevance were observed in the mentioned cases. The data flowchart of our prenatal and postnatal cohorts is provided in Fig. 1.

**Table 1:** Findings of 201 fetuses with 16p13.11 deletion (60 cases) and duplication (141 cases)

CNV	Sample types (%)			Interval (%)			Inheritance (%)				Pregnancy outcome (%)		
	VS	AF	UCB	I + II	II	II + III	Donovo	Paternal	Maternal	Refused	Newborn		TP
Deletion	13.3% (8/60)	68.3% (41/60)	18.3% (11/60)	71.7% (43/60)	5.0% (3/60)	23.3% (14/60)	26.9% (7/26)	26.9% (7/26)	46.2% (12/26)	34	63.3% (38/60)		36.7% (22/60)
Duplication	18.4% (26/141)	66.0% (93/141)	15.6% (22/141)	75.2% (106/141)	10.6% (15/141)	14.2% (20/141)	14.5% (9/62)	41.9% (26/62)	43.6% (27/62)	79	73.8% (104/141)		26.2% (37/141)

CNV	Prenatal diagnosis indications (%)							Postnatal outcome					
	Abnormal ultrasound	Abnormal NIPT results	High-risk screening	Genetic factors	Adverse pregnancy history	Advanced maternal age	Pregnancy exposure	Gender of newborn (%)		Delivery week (%)		Postnatal phenotypes (%)	
								Male	Female	Preterm	Term	Abnormal	Normal
Deletion	51.7% (31/60)	1.7% (1/60)	10.0% (6/60)	16.7% (10/60)	11.7% (7/60)	5.0% (3/60)	3.3% (2/60)	55.3% (21/38)	44.7% (17/38)	5.3% (2/38)	94.7% (36/38)	25% (9/36)	75% (27/36)
Duplication	41.1% (58/141)	6.4% (9/141)	13.5% (19/141)	17.7% (25/141)	9.9% (14/141)	8.5% (12/141)	2.1% (3/141)	60.6% (63/104)	39.4% (41/104)	11.5% (12/104)	88.5% (92/104)	17.5% (18/103)	82.5% (85/103)

Abnormal ultrasound include structural malformations, abnormal soft markers and other abnormalities. Genetic factors include parents with intellectual disability, abnormal karyotypes, CNVs and carried the same thalassemia gene. Advanced maternal age, ≥ 35 years. Pregnancy exposure include poor exposure and medication history during early pregnancy. Above 4 cases of fetuses with deletion and 10 cases of fetuses with duplication had additional genomic abnormalities. Above 2 cases of newborns with deletion and one case of newborns with duplication had additional genomic abnormalities, not included in the calculation of the rate of abnormal postnatal phenotypes

*VS* villus sampling, *AF* amniotic fluid, *UCB* umbilical cord blood, *TP* termination of pregnancy, *NIPT* noninvasive prenatal testing, Preterm, delivery week < 37 w, Term, delivery week ≥ 37 w

**Table 2:** Abnormal ultrasound characteristics of 89 fetuses with 16p13.11 deletion (31 cases) and duplication (58 cases)

CNV	Isolated/Non-isolated ultrasound	Ultrasound classifications	Ultrasound characteristics	n	Case index
Deletion (30 cases)	Isolated ultrasound (23 cases)	Soft markers (19 cases)	Choroid plexus cysts	6	3,11,20,21,33,38
			Dilated renal pelvis	3	6,8,41
			Echogenic intracardiac focus	3	4,31,40
			Thickened nuchal translucency	2	22,50
			Permanent left superior vena cava	2	34,49
			Lateral ventricle broadening	1	1
			Short femur	1	36
			Single umbilical artery	1	24
		Other abnormalities (4 cases)	Subependymal cysts	1	16
			Fast heart rhythm	1	23
			Thickened placenta	1	39
			Abdominal mixed mass	1	26
	Non-isolated ultrasound (7 cases)	Structural malformations (3 cases)	Cardiovascular system	2	12,48
			Urinary system	1	19
		Structural malformations&Soft markers (1 case)	Cardiovascular system, Echogenic intracardiac focus, Single umbilical artery	1	29
		Structural malformations&Other abnormalities (1 case)	Urinary system, FGR	1	46
		Soft markers (≥ 2) (1 case)	Dilated renal pelvis, Lateral ventricle broadening	1	17
		Other abnormalities&Soft markers (1 case)	Colon dilatation, Cavum septum pellucidum not displayed	1	47
Deletion&Additional genomic abnormalities (1 case)	Non-isolated ultrasound (1 case)	Other abnormalities&Soft markers (1 case)	Polyhydramnios, Dilated renal pelvis	1	60
Duplication (54 cases)	Isolated ultrasound (40 cases)	Structural malformations (4 cases)	Cleft lip and palate	1	61
			Urinary system	3	95,124,152
		Soft markers (24 cases)	Thickened nuchal translucency	5	67,139,163,171,186
			Choroid plexus cysts	4	83,123,153,182
			Absent or hypoplastic nasal bone	3	85,93,98
			Aberrant right subclavicular artery	2	136,149
			Echogenic intracardiac focus	2	81,101
			Hyperechogenic bowel	2	112,181
			Dilated renal pelvis/hydronephrosis	2	89,105
			Persistent right umbilical vein	1	190
			Short femur and humerus	1	133
			Single umbilical artery	1	88
			Ventriculomegaly	1	147
		Other abnormalities (12 cases)	Fetal arhythmia	1	66
			Large head circumference	1	68
			Liquid dark area in the right upper fetal bladder	1	69
			Widened inferior vena cava	1	107
			Arachnoid cyst	1	115
			Widened inner diameter of rectum	1	117
			Thickened placenta	1	122
			Fetal left mandible subcutaneous fluid mass	1	134
			Small head circumference	1	165
			FGR	1	87
			Oligohydramnios	2	76, 154
	Non-isolated ultrasound (14 cases)	Structural malformations (2 cases)	Cardiovascular system	2	144, 166
		Structural malformations&Soft markers (1 case)	Syndactyly, Thickened nuchal translucency	1	169
		Soft markers (≥ 2) (4 cases)	Choroid plexus cysts, Aberrant right subclavicular artery	1	125
			Echogenic intracardiac focus, Small cavum septum pellucidum	1	129
			Hydronephrosis, Dilated renal pelvis	1	148
			Choroid plexus cysts, Thickened nuchal translucency	1	174
		Other abnormalities&Soft markers (6 cases)	Oligohydramnios, Small magenblase, Thickened placenta, Hyperechogenic bowel	1	64
			Abnormal echo in the liver, Enlarged cisterna magnal	1	75
			Oligohydramnios, Shallow and flat parietal occipital sulcus, Small cavum septum pellucidum	1	140
			Abnormal echo in the liver, Hyperechogenic bowel	1	155
			Subependymal cysts, Absent or hypoplastic nasal bone, Widened cavum septum pellucidum	1	156
			FGR, Choroid plexus cysts, Hyperechogenic bowel	1	160
		Other abnormalities (1 case)	Oligohydramnios, FGR	1	71
Duplication&Additional genomic abnormalities (4 cases)	Isolated ultrasound (2 cases)	Soft markers (1 case)	Thickened nuchal translucency	1	201
		Other abnormalities (1 case)	Oligohydramnios	1	195
	Non-isolated ultrasound (2 cases)	Structural malformations (1 case)	Nuchal cystic hygroma, Fetal hydrops	1	197
		Structural malformations&Soft markers (1 case)	Cerebellar hypoplasia, Absent or hypoplastic nasal bone, Single umbilical artery	1	199

*FGR* fetal growth restriction

**Table 3:** Abnormal postnatal phenotypes of 139 newborns with 16p13.11 deletion (36 cases) and duplication (103 cases)

CNV	Abnormal clinical phenotypes	n (%)	Case index
Deletion (9 cases)	DD (growth, motor and language development)	16.7% (6/36)	1, 5, 12, 14, 36, 43
	Immune disorders (urticaria and dermatitis)	5.6% (2/36)	36, 41
	Cardiovascular abnormalities (CHD, pulmonary stenosis, patent ductus arteriosus, tricuspid regurgitation, second foramen atrial septal defect (type II), venous sinus defect, coronary sinus defect, patent foramen ovale)	5.6% (2/36)	12, 31
	Dysmorphic features (torticollis)	5.6% (2/36)	41, 43
	Congenital abnormalities (inguinal hernia, cystic teratoma, conjunctivitis, bilateral testicular hydrocele, mild anemia and intestinal adhesions)	8.3% (3/36)	12, 26, 31
Duplication (18 cases)	DD (growth, motor and language development)	5.8% (6/103)	74, 100, 128, 151, 157, 160
	Immune disorders (dermatitis, angular cheilitis and Kawasaki disease)	5.8% (6/103)	76, 100, 123, 134, 141, 175
	Cardiovascular abnormalities (CHD, atrioventricular block, patent ductus arteriosus, patent foramen ovale, tricuspid regurgitation, second foramen atrial septal defect (type II), venous sinus defect, coronary sinus defect, aortic stenosis, increased anterior blood flow velocity of pulmonary valve, pulmonary regurgitation,sinus rhythm, occasional premature beat)	5.8% (6/103)	66, 69, 123, 136, 144, 160
	Ophthalmological abnormalities (eye subconjunctival hemorrhage, immature retina, unvascularized retina, myopia and astigmatism)	4.9% (5/103)	100, 123, 128, 136, 151
	Urogenital system abnormalities (concealed penis and cryptorchidism)	1.9% (2/103)	128, 157
	Dysmorphic features (torticollis)	1.9% (2/103)	100, 105
	Behavioural disturbances (hyperspasmia and epilepsy)	1% (1/103)	108
	Congenital abnormalities (feeding intolerance, umbilical hernia, cystic teratoma, lymphangioma, pigmentation, depigmented nevus and nevus flammeus, subependymal hemorrhage, conjunctivitis, lacrimal duct stenosis, eye nasolacrimal duct obstruction)	10.7% (11/103)	69, 76, 100, 105, 128, 134, 136, 144, 151, 157, 180

There are no additional genomic abnormalities with clinical relevance in above 139 newborns

*DD* developmental delay, *CHD* congenital heart disease

**Table 4:** Abnormal clinical phenotypes of 14 patients with 16p13.11 deletion (3 cases) and duplication (11 cases)

CNV	Abnormal clinical phenotypes	Case index
Deletion (3 cases)	DD (growth, motor and global development)	215, 216
	Immune disorders (tinea versicolor, immunodeficiency and autoimmune diseases)	216, 217
	Behavioural disturbances (epilepsy)	215
	Brain abnormalities (myelin developmental delayed)	215
	Ophthalmological abnormalities (nystagmus)	215
Duplication (11 cases)	DD (idiopathic short stature, motor and global development)	225, 228, 283, 284
	Behavioural disturbances (ADHD, ASD, sensory disorder and epilepsy)	227, 275, 283, 284
	ID and MR	228, 277, 283, 289
	Immune disorders (pityrosporum folliculitis and dermatitis)	255, 278
	Respiratory diseases (severe pneumonia, respiratory failure, acute respiratory distress syndrome, bronchopulmonary dysplasia)	224, 284
	Cardiovascular abnormalities (central atrial septal defect (foramen ovale))	284
	Brain abnormalities (congenital septum pellucidum abnormalities)	284
	Urogenital system abnormalities (cryptorchidism)	284
	Congenital abnormalities (feeding intolerance)	284
	Other (liver function impairment)	224

There are no additional genomic abnormalities with clinical relevance in above 14 patients

*DD* developmental delay, *ASD* autism spectrum disorder, *ADHD* attention-deficit hyperactivity disorder, *ID* intellectual disability, *MR* mental retardation

### Prenatal sample

#### Prenatal sample type

In our database, the proportions of CVS, amniocentesis and cordocentesis procedures performed in cases of Del were 13.3% (8/60), 68.3% (41/60) and 18.3% (11/60), respectively, and those in cases of Dup were 18.4% (26/141), 66.0% (93/141) and 15.6% (22/141), respectively.

#### Interval

CMA revealed that 71.7% (43/60) of Del cases and 75.2% (106/141) of Dup cases were located inside intervals I and II, 5.0% (3/60) of Del cases and 10.6% (15/141) of Dup cases were located inside interval II only, and 23.3% (14/60) of Del cases and 14.2% (20/141) of Dup cases were located inside intervals II and III.

#### Pedigree analysis

In addition to the 26 fetuses, the parents of the remaining 34 fetuses refused pedigree verification in the cohort of Del cohort. Among the 26 cases, 7 (26.9%) were de novo, and 19 cases were either paternally (n = 7) or maternally (n = 12) inherited. In the Dup cohort, the parents of 79 fetuses refused pedigree analysis, whereas in the other 62 cases, 9 (14.5%) were de novo, and 53 cases were either paternally (n = 26) or maternally (n = 27) inherited.

#### Prenatal diagnosis indications

Each case was classified according to its most important indication. Indications were listed in order of importance: abnormal ultrasound, abnormal noninvasive prenatal testing (NIPT) results, high risk of serum screening for pregnant women, genetic factors of parents, adverse pregnancy history, pregnancy at advanced maternal age, and pregnancy exposure. Abnormal ultrasound characteristics included structural malformations, abnormal soft markers and other anomalies. Genetic factors included parents with ID, abnormal karyotypes, CNVs and those who carried the same thalassemia gene. Pregnancy exposure includes poor exposure and medication history during early pregnancy.

#### Prenatal ultrasound phenotype

Among the 60 fetuses with Del, 31 (51.7%) presented abnormal ultrasound characteristics. One case had an 11p15.3 duplication and had polyhydramnios and a dilated renal pelvis. Twenty-three fetuses presented with a single isolated ultrasound sign, whereas 7 fetuses presented with several ultrasound signs. There were 6 cases of isolated choroid plexus cysts, 3 cases of isolated dilated renal pelvis, and 3 cases of isolated echogenic intracardiac focus. Additionally, 3 fetuses had cardiovascular malformations, and 2 had urinary malformations.

Among the 141 fetuses with Dup, 58 (41.1%) presented abnormal ultrasound characteristics. Four of the fetuses had additional genomic abnormalities (Xp22.31 deletion, XO, trisomy 21 or abnormal chromosome X) and had several ultrasound signs. Forty fetuses presented with a single isolated ultrasound sign, whereas 14 fetuses presented with several ultrasound signs. There were 5 cases of isolated thickened nuchal translucency, 4 cases of isolated choroid plexus cysts, and 3 cases of isolated absent or hypoplastic nasal bone. Additionally, 3 fetuses had urinary malformations, 2 had cardiovascular malformations, 1 had cleft lip and palate, and 1 had syndactyly. There were 2 cases of fetal growth restriction (FGR), 2 cases of oligohydramnios, and 1 case of FGR and oligohydramnios.

#### Postnatal outcome of follow-up

In accordance with the decision of the parent, termination of pregnancy was performed for 22 fetuses in the Del cohort. Among the newborns, 55.3% (21/38) were male and 44.7% (17/38) were female, with 5.3% (2/38) being delivered preterm. Nine (25%) newborns with abnormal postnatal phenotypes are listed in Table 3 and depicted in pink in Table S1. Among the 36 newborns without additional genomic abnormalities with clinical relevance, 16.7% (6/36) were diagnosed with DD. A total of 5.6% (2/36) had immune disorders, 5.6% (2/36) had cardiovascular abnormalities, and 5.6% (2/36) had torticollis. Cases also included inguinal hernia, cystic teratoma, conjunctivitis, bilateral testicular hydrocele, mild anemia and intestinal adhesions.

Additionally, termination of pregnancy was performed for 37 fetuses in the Dup cohort. Among the newborns, 60.6% (63/104) were male and 39.4% (41/104) were female, with 12 (11.5%) being delivered preterm. Eighteen (17.5%) newborns with abnormal postnatal phenotypes are listed in Table 3 and highlighted in blue in Table S2. Among the 103 newborns without additional genomic abnormalities with clinical relevance, 5.8% (6/103) were diagnosed with DD. A total of 5.8% (6/103) had immune disorders, 5.8% (6/103) had cardiovascular abnormalities, 4.9% (5/103) had ophthalmological abnormalities, 1.9% (2/103) had torticollis, and 1.9% (2/103) had urogenital system abnormalities. Newborns also presented with epilepsy, feeding intolerance, umbilical hernia, cystic teratoma, lymphangioma, pigmentation, depigmented nevus and nevus flammeus, subependymal hemorrhage, conjunctivitis, lacrimal duct stenosis and eye nasolacrimal duct obstruction.

### Peripheral blood samples

#### Clinical phenotypes

We detected 22 samples with Del, including 18 cases from the pedigree analysis and 2 cases from the health checkup. Three patients with abnormal phenotypes are listed in Table 4 and depicted in pink in Table S5. A female presented tinea versicolor only. Two children had DD, epilepsy, nystagmus and delayed development of myelin, immunodeficiency and autoimmune diseases.

A total of 69 samples with Dup were identified, comprising 55 cases from pedigree analysis and 5 cases from health checkups. Eleven patients with abnormal phenotypes are listed in Table 4 and highlighted in blue in Table S5. Two adults presented with pityrosporum folliculitis or dermatitis only. Two adults had ID. In the clinical referral series, 7 children were diagnosed with acute respiratory distress syndrome, idiopathic short stature, DD, ID and MR, ADHD, sensory disorder, ASD, epilepsy, feeding intolerance, bronchopulmonary dysplasia, congenital septum pellucidum abnormalities, central atrial septal defect (foramen ovale) and cryptorchidism.

## Discussion

Based on a large East Asian prenatal cohort in this recurrent region to date, this study revealed that the detection rates of Del and Dup in southern China were 0.08% (60/79,136) and 0.18% (141/79,136), respectively, which are more representative than those reported in seven small Chinese cohorts. The detection rates of 16p13.11 Del were 0.10% (15/15,263) in 29 Chinese provinces [11], 0.09% (13/15,051) in Chinese Taiwan [12], 0.08% (6/7,617) in Henan [13], 0.06% (5/9,000) in Fujian [14] and 0.06% (4/7,078) in Chengdu [15]. The detection rates of 16p13.11 Dup were 0.06% (9/15,051) in Chinese Taiwan [12], 0.32% (24/7,617) in Henan [13], 0.17% (15/9,000) in Fujian [14], 0.10% (7/7,078) in Chengdu [15], 0.63% (8/1,261) in Chinese Hong Kong [16] and 0.43% (15/3,451) in Shandong [17]. The detection rate in southern China is critical for understanding this recurrent region and for providing genetic counseling within the East Asian population. However, most studies have focused on the frequency of CNVs in patient cohorts. The 16p13.11 Del genotype was present in 0.5–0.6% of patients with epilepsy [4, 5], 0.12% of patients with schizophrenia [6], 0.15% of patients with neurodevelopmental disorders [18] and 0.15% of patients with various abnormal phenotypes [19]. The 16p13.11 Dup genotype was present in 0.30% of patients with schizophrenia [6], 0.27% of patients with neurodevelopmental disorders [18] and 0.19% of Chinese pediatric patients populations with DD [20]. In addition, in the general population, the prevalence of Del is approximately 0.04–0.05% [18, 19], and that of Dup is approximately 0.12% [18]. It follows that the detection rate in the prenatal cohort was lower than that in the patient cohort but higher than that in the general population, providing a reference for the diagnosis and monitoring of this disease.

An estimation of the frequency of de novo occurrence and penetrance in different ethnic populations is essential for genetic counseling. In our prenatal cohort, the frequency of the de novo occurrence of Del was 26.9%, and that of Dup was 14.5%, which was slightly higher than that in an American cohort of Del (21.7%) [19] and a French and Belgian cohort of Dup (8.8%) [21], indicating that these populations are distinct from the Chinese population. Notably, 73.1% of Del and 85.5% of Dup were inherited, and most of them had parents with no significant abnormalities. Among these, 28.6% (4/14) of Del and 15.9% (7/44) of Dup had abnormal phenotypes during the postnatal outcome follow-up (Tables S1 and S2), indicating incomplete penetrance in 16p13.11 Del/Dup. In addition, the penetrance of 16p13.11 Del has been reported to be 13.1% in the United States [19] and 17% in the UK Biobank [22], whereas the penetrance of Dup is 8.43% in France and Belgium [21], 7% in the UK Biobank [22] and 10.6% in Europe and North America [23]. However, the rates of abnormal postnatal phenotypes in our cohort were 25% (9/36) for Del and 17.5% (18/103) for Dup. This difference may be attributed to the fact that our study focused on prenatal fetuses and long-term follow-up for various clinical abnormalities, whereas prior research primarily involved children or adolescents with DD or ID. DD was the most common clinical abnormality, affecting approximately 16.7% (6/36) of Del and 5.8% (6/103) of Dup (Table 3). Immune disorders were observed in 5.6% (2/36) of Del and 5.8% (6/103) of Dup, confirming the broad impact of the 16p13.11 Del/Dup phenotypes on the immune system. Immune disorders have been previously reported only in association with 16p13.11 Dup [24]. Notably, torticollis, concealed penis and cryptorchidism are a series of previously unnoticed related phenotypes that warrant further investigation. Moreover, among Dup, 4.9% (5/103) exhibited ophthalmological abnormalities, whereas 5.8% (6/103) had cardiovascular abnormalities. Taken together, it is crucial to refer individuals with 16p13.11 Del/Dup for growth and developmental examination; neurodevelopmental and behavioral diagnosis; and cardiovascular, ophthalmological, urogenital and immune system assessments.

To date, fetal 16p13.11 Del/Dup phenotypes have rarely been described. In our cohort, 51.7% (31/60) of Del and 41.1% (58/141) of Dup presented abnormal ultrasound findings; that is, ultrasound characteristics should be adequately considered during clinical consultations. Isolated ultrasound soft markers were the most common ultrasound signs in our cohort (Table 2). An echogenic Echogenic intracardiac focus and an aberrant right subclavicular artery warrant particular attention, as they may be associated with congenital cardiac abnormalities in the postnatal phenotypes (Tables S3 and S4). Studies have reported that 16p13.11 Del is associated with thickened nuchal translucency, whereas 16p13.11 Dup is closely related to the echogenic bowel [11, 14]. However, only 3.3% (2/60) of Del with isolated thickened nuchal translucency were detected, whereas 10.0% (6/60) exhibited isolated choroid plexus cysts in our cohort. Moreover, only 1.4% (2/141) of Dup exhibited isolated echogenic bowel, whereas 4.6% (6/141) presented isolated thickened nuchal translucency (Table 2). Therefore, isolated choroid plexus cysts may be the most relevant ultrasound soft marker for Del, whereas isolated thickened nuchal translucency appears to be more closely associated with Dup. Moreover, cardiovascular (3 Del and 2 Dup) and urinary (2 Del and 3 Dup) malformations were the most frequently detected structural abnormalities on ultrasound. These cases also showed congenital cardiac abnormalities in terms of postnatal outcomes, suggesting that a more comprehensive cardiac and renal evaluation should be conducted for prenatal diagnosis and postnatal assessments. In addition, oligohydramnios and FGR have raised concerns in Dup; thus, a more careful assessment of intrauterine fetal growth and development should be performed during genetic counseling. Considering the strong correlations between 16p13.11 Del/Dup and ultrasound soft markers, combining prenatal CMA detection is necessary for the diagnosis of this recurrent region.

**Fig. 2:**
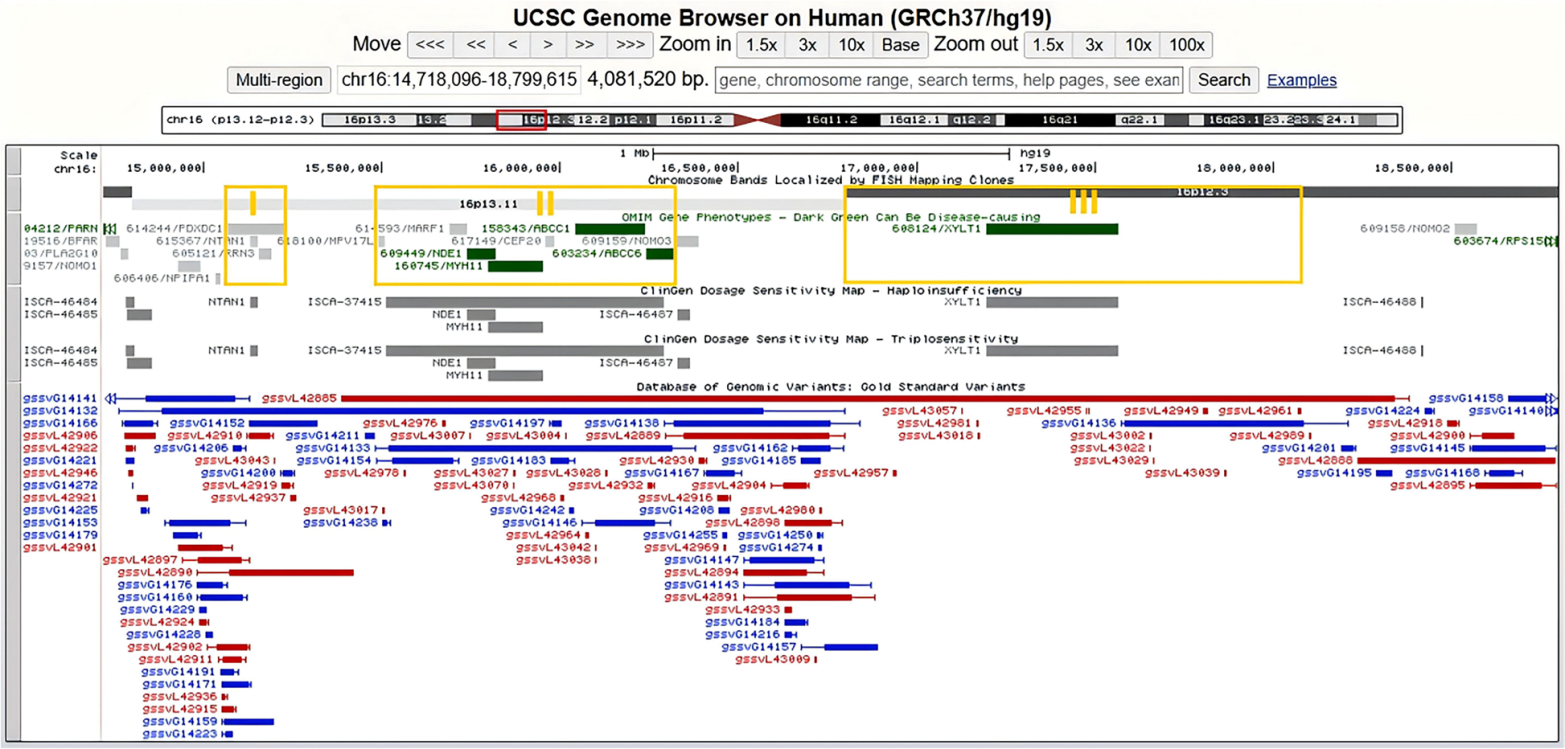
Genomic position of the 16p13.12-p12.3 region. Produced with the University of California, Santa Cruz Genome Browser (http://www.genome.ucsc.edu). Genomic region is subdivided into three intervals (I, II and III) referred to in the article by Ingason et al. are shown as yellow frame

The recurrent 16p13.11 region is subdivided into three main intervals (I, II and III), each flanked by sequences rich in low copy repeats (LCRs) (Fig. 2) [6]. In our prenatal cohort, 5.0% (3/60) of Del II and 10.6% (15/141) of Dup II presented only with DD, cystic teratoma, torticollis, unvascularized retina and pigmentation at the postnatal outcome follow-up. Furthermore, Del II and Dup II were not found in our patient cohort. These 14 patients with Del II + III, Dup I + II and Dup II + III exhibited a wide range of abnormal phenotypes, including DD, ID/MR, ADHD, ASD, sensory disorder, epilepsy, cryptorchidism, respiratory diseases, immune disorders, and cardiovascular, ophthalmological, brain and congenital abnormalities (Table 4). These findings indicated that CNVs extending to intervals I + II or II + III appeared to be associated with a broader range of phenotypes. However, further studies are needed to confirm the gene‒gene interaction effects involved. Interestingly, it has been reported that 16p13.11 Dup is a risk factor for thoracic aortic aneurysm and dissection [9], predisposing individuals to defective cardiac left–right patterning and laterality disorders [25]. However, none of the above cardiovascular abnormalities were detected in our prenatal or patient cohort.

A limitation of this study is the absence of gene sequencing to identify other potential pathological variants. More clinical cases should be included and discussed, with long-term follow-up conducted and further genetic testing performed in future studies. Based on the 2024 American College of Medical Genetics and Genomics, ClinGen technical standards for the interpretation and reporting of constitutional CNVs, cohort studies and local databases, these high-frequency 16p13.11 CNVs may be classified as variants of uncertain significance or low-penetrance risk factors depending on the fetal phenotypes and inheritance patterns in the prenatal setting. Given the incomplete penetrance and variable expressivity of 16p13.11 CNVs, the results for the fetus are difficult to predict, and cautious interpretation and comprehensive genetic counseling are warranted.

## Conclusion

This study presents a large East Asian prenatal cohort and a smaller cohort of pediatric and adult patients with 16p13.11 Del/Dup, providing critical reference data for genetic counseling in East Asian populations. Further comprehensive studies incorporating cardiac and renal evaluations are recommended for prenatal diagnosis. It is essential to refer individuals with 16p13.11 Del/Dup for postnatal assessment, including growth and developmental monitoring; neurodevelopmental and behavioral evaluation; and screening of the cardiovascular, ophthalmological, urogenital and immune systems. Furthermore, fetal outcomes are difficult to predict due to the low penetrance of 16p13.11 Del/Dup. Therefore, cautious interpretation and thorough genetic counseling are strongly warranted.

## Supplementary Information

Supplementary Material 1: Table S1. CMA, prenatal diagnosis indications and outcome follow-up of 60 fetuses with 16p13.11 deletion. Table S2. CMA, prenatal diagnosis indications and outcome follow-up of 141 fetuses with 16p13.11 duplication. Table S3. Ultrasound characteristics and outcome follow-up of 31 fetuses with 16p13.11 deletion. Table S4. Ultrasound characteristics and outcome follow-up of 58 fetuses with 16p13.11 duplication. Table S5. CMA and clinical phenotypes of 91 peripheral blood samples with 16p13.11 deletion and duplication.

## Data Availability

All data generated or analysed during this study are included in this published article and its supplementary information files.
