# Supplementary material for: 16p13.11 deletion/duplication: a large cohort study on prenatal diagnosis, postnatal outcomes, and phenotypic manifestations
Source: BMC Pregnancy Childbirth. 2025 Dec 23;25:1317. doi: 10.1186/s12884-025-08467-2 (PMC12729671; doi:10.1186/s12884-025-08467-2)
Supplement: Supplementary file 1 — Supplementary Material 1: Table S1. CMA, prenatal diagnosis indications and outcome follow-up of 60 fetuses with 16p13.11 deletion. Table S2. CMA, prenatal diagnosis indications and outcome follow-up of 141 fetuses with 16p13.11 duplication. Table S3. Ultrasound characteristics and outcome follow-up of 31 fetuses with 16p13.11 deletion. Table S4. Ultrasound characteristics and outcome follow-up of 58 fetuses with 16p13.11 duplication. Table S5. CMA and clinical phenotypes of 91 peripheral blood samples with 16p13.11 deletion and duplication. [file 12884_2025_8467_MOESM1_ESM.docx]

| Table S1. CMA, prenatal diagnosis indications and outcome follow-up of 60 fetuses with 16p13.11 deletion. | | | | | | | | | | | | | | | | | | |
| --- | --- | --- | --- | --- | --- | --- | --- | --- | --- | --- | --- | --- | --- | --- | --- | --- | --- | --- |
| Case index | Maternal age (y) | Sample type | CMA | OMIM gene | Size (Mb) | Interval | Inheritance | Chromosome karyotype | Prenatal diagnosis indications | Abnormal ultrasound | Pregnancy outcome | Fetus gender | Delivery date | Delivery week (w) | Birth weight (g) | Birth length (cm) | Physical examination | Outcome follow-up |
| 1 | 36 | AF | arr[GRCh37] 16p13.11(15493046-16303388)×1 | 7 | 0.81 | II | Paternal | 46,XY | Left lateral ventricle broadening (1.0cm), advanced maternal age | Yes | Cesarean | Male | 2015/11/16 | 39 | 2500 | / | 3 months: L=58cm(<-1sd); W=5.25kg(<-1sd); HC=38cm(<-2sd) 12 months: L=70cm(<-2sd); W=8.2kg(<-1sd); HC=43cm(<-2sd) 24 months: L=80cm(<-2sd); W=9.6kg(<-2sd); HC=47cm(<Mean) 55 months: L=95cm(<-2sd); W=13kg(<-2sd); HC=49cm(<-1sd) 66 months: L=100cm(<-2sd); W=14.1kg(<-2sd); HC=49cm 79 months: L=105cm(<-2sd); W=15kg(<-2sd) | Full-term delivery and SGA, Physical examination shown short stature and low weight (GDD) |
| 2 | 33 | AF | arr[GRCh37] 16p13.11(15052746-16289532)×1 | 10 | 1.24 | I+II | denovo | 46,XN | Adverse pregnancy history | No | TP |  |  |  |  |  |  |  |
| 3 | 22 | AF | arr[GRCh37] 16p13.11(15052746-16688008)×1 | 11 | 1.64 | I+II | Maternal | 46,XX | Left choroid plexus cysts, fetal father with deaf and dumb | Yes | Cesarean | Female | 2016/6/7 | 40 | 3250 | 50 | 3 months: L=58cm(<Mean); W=5kg(<-1sd); HC=37cm(<-2sd) 6 months: L=61cm(<-2sd); W=7kg(<Mean); HC=40.2cm(<-1sd) 36 months: L=85cm(<-2sd); W=11kg(<-1sd); HC=46.6cm(<-1sd) | Full-term delivery and AGA, Well survivor |
| 4 | 32 | UCB | arr[GRCh37] 16p13.11(15052746-16289532)×1 | 10 | 1.24 | I+II | Maternal | 46,XY | Left echogenic intracardiac focus | Yes | Eutocia | Male | 2016/9/22 | 39 | 3000 | 50 | 1 months: L=54.5cm(<Mean); W=4.1kg(<Mean) 6 months: L=66cm(<Mean); W=7kg(<-1sd); HC=41.3cm(<-1sd) 12 months: L=71.6cm(<-1sd); W=7.8kg(<-1sd); HC=44.6cm(<-1sd) 24 months: L=81cm(<-2sd); W=9.6kg(<-2sd); HC=46.5cm(<-1sd) 36 months: L=91cm(<-1sd); W=10.8kg(<-2sd); HC=47.8cm(<-1sd) 57 months: L=111cm(>Mean); W=17kg(<Mean) 79 months: L=115cm(<Mean); W=17kg(<-1sd) | Full-term delivery and AGA, Well survivor |
| 5 | 27 | AF | arr[GRCh37] 16p13.11(15052746-16289532)×1 | 10 | 1.24 | I+II | denovo | 46,XX | Parent carried α-thalassemia gene | No | Cesarean | Female | 2017/6/9 | 42 | 3530 | 52 | 1 months: L=57cm(>+1sd); W=4.5kg(>Mean) 3 months: L=60cm(>Mean); W=5.1kg(<-1sd); HC=41cm(>+1sd) 12 months: L=71.5cm(<Mean); W=7.5kg(<-1sd); HC=45cm(>Mean) 38 months: L=86.7cm(<-2sd); W=10.5kg(<-2sd); HC=49.5cm(>Mean) 47 months: L=91.5cm(<-2sd); W=11.2kg(<-2sd) 59 months: L=96cm(<-2sd); W=13.2kg(<-2sd) 68 months: L=102cm(<-2sd); W=14.55kg(<-2sd) 84 months: L=108cm(=-3sd); W=15.5kg(<-2sd) | Full-term delivery and AGA, Physical examination shown short stature and low weight (GDD) |
| 6 | 33 | AF | arr[GRCh37] 16p13.11(15052746-16289532)×1 | 10 | 1.24 | I+II | Paternal | 46,XY | Bilateral dilated renal pelvis (Left, 1.07cm; Right, 0.78cm) | Yes | Eutocia | Male | 2017/7/10 | 40 | 3500 | 52 | 1 months: L=56cm(>Mean); W=4.5kg(=Mean) 3 months: L=61cm(<Mean); W=5.6kg(<-1sd); HC=40cm(<Mean) 6 months: L=67cm(<Mean); W=6.2kg(<-2sd); HC=42cm(<-1sd) 8 months: L=68cm(<-1sd); W=6.6kg(<-2sd); HC=42.5cm(<-1sd) 39 months: L=92cm(<-1sd); W=12.8kg(<-1sd); HC=48cm(<-1sd) 57 months: L=103cm(<-1sd); W=15kg(<-1sd) 69 months: L=107cm(<-1sd); W=16.2kg(<-1sd) 80 months: L=112cm(<-1sd); W=18kg(<-1sd) | Full-term delivery and AGA, Well survivor |
| 7 | 32 | VS | arr[GRCh37] 16p13.11p13.12(14618311-16272508)×1 | 15 | 1.65 | I+II | Refused | 46,XN | Parent carried α-thalassemia gene | No | TP |  |  |  |  |  |  | Thalassemia result, αWSα/αα, β41-42/βN, Fetus with mild thalassemia |
| 8 | 40 | AF | arr[GRCh37] 16p13.11(15052746-16303388)×1 | 10 | 1.25 | I+II | Refused | 46,XY | Bilateral dilated renal pelvis (Left, 0.68cm; Right, 0.46cm), advanced maternal age | Yes | Cesarean | Male | 2018/8/18 | 38 | 3150 | 50 | 3 months: L=60cm(<-1sd); W=6kg(<-1sd); HC=40cm(<-1sd) 6 months: L=67cm(=-1sd); W=7.5kg(<Mean); HC=42cm(<-1sd)  12 months: L=75.5cm(<Mean); W=9.5kg(<Mean); HC=46cm(<Mean) 18 months: L=80cm(<Mean); W=10kg(<Mean); HC=47cm(<Mean) 57 months: L=105cm(<Mean); W=16kg(<Mean); HC=50cm(<Mean) 69 months: L=112cm(<Mean); W=20kg(<Mean) | Full-term delivery and AGA, Well survivor |
| 9 | 37 | AF | arr[GRCh37] 16p13.11(15052746-16303388)×1 | 10 | 1.25 | I+II | Refused | 46,XN | Adverse pregnancy history, advanced maternal age, medication history in early pregnancy, pregnant women with schizophrenia | No | TP |  |  |  |  |  |  |  |
| 10 | 29 | AF | arr[GRCh37] 16p13.11(15052746-16303388)×1 | 10 | 1.25 | I+II | Refused | 46,XN | High-risk screening for trisomy 21 | No | TP |  |  |  |  |  |  |  |
| 11 | 26 | AF | arr[GRCh37] 16p13.11(15052746-16303388)×1 | 10 | 1.25 | I+II | Refused | 46,XN | Bilateral choroid plexus cysts (Left, 0.5×0.4cm; Right, 0.6×0.3cm), high-risk screening for trisomy 21, adverse pregnancy history (G5P2A3, a girl with anal atresia and die young) | Yes | TP |  |  |  |  |  |  |  |
| 12 | 21 | UCB | arr[GRCh37] 16p13.11(15493046-18156351)×1 | 9 | 2.66 | II+III | Refused | 46,XY | Pulmonary stenosis (0.2cm), mild tricuspid regurgitation, medication history in early pregnancy, pregnant women with epilepsy | Yes | Eutocia | Male | 2018/12/18 | 35 | 2600 | 48 | 1 months: L=49cm(<-2sd); W=3kg(<-2sd); HC=33.5cm(<-3sd) 38 months: L=88cm(<-2sd); W=11.3kg(<-2sd) 53 months: L=94cm(<-2sd); W=13kg(<-2sd) 64 months: L=101cm(<-2sd); W=15kg(=-2sd) | Preterm delivery and AGA, Mild pulmonary stenosis, Patent ductus arteriosus, Mild tricuspid regurgitation and inguinal hernia, Physical examination shown short stature and low weight (GDD) |
| 13 | 29 | AF | arr[GRCh37] 16p13.11p12.3(15052746-16899495)×1 | 11 | 1.85 | I+II | denovo | 46,XN | Adverse pregnancy history | No | TP |  |  |  |  |  |  |  |
| 14 | 38 | AF | arr[GRCh37] 16p13.11(15052746-16289532)×1 | 10 | 1.24 | I+II | Refused | 46,XY | High-risk screening for trisomy 18 and 21, advanced maternal age | No | Eutocia | Male | 2019/4/18 | 40 | 3300 | 50 | 8 months: L=66cm(<-1sd); W=7.2kg(<-1sd); HC=42.2cm(<-1sd) 12 months: L=70cm(<-1sd); W=8.1kg(<-1sd); HC=44.3cm(<-1sd) 24 months: L=81.2cm(<-2sd); W=10.7kg(<-1sd) 30 months: L=84.2cm(<-2sd); W=11.5kg(<-1sd); HC=47.5cm(=-1sd) 49 months: L=95cm(<-2sd); W=13.38kg(<-1sd) 60 months: L=96.5cm(<-2sd); W=14.4kg(<-1sd) | Full-term delivery and AGA, Physical examination shown short stature and low weight (GDD) |
| 15 | 32 | AF | arr[GRCh37] 16p13.11(15052746-16303388)×1 | 10 | 1.25 | I+II | Refused | 46,XY,9qh+ | High-risk screening for trisomy 21 | No | Cesarean | Male | 2019/4/3 | 41 | 4350 | 56 | 1 months: L=59.6cm(>+2sd); W=5.6kg(>+1sd); HC=39.2cm(>+1sd) 3 months: L=67cm(>+2sd); W=7.4kg(>+1sd); HC=42cm(>+1sd) 12 months: L=76.7cm(>Mean); W=9.7kg(>Mean); HC=47cm(>Mean) 24 months: L=87.7cm(<Mean); W=13kg(>Mean); HC=49cm(>Mean) 36 months: L=95.9cm(<Mean); W=15kg(>Mean); HC=50.5cm(>Mean) 49 months: L=103cm(<Mean); W=17.4kg(>Mean) 61 months: L=108.5cm(<Mean); W=19.3kg(>Mean) | Full-term delivery and LGA, Well survivor |
| 16 | 35 | UCB | arr[GRCh37] 16p13.11(15493046-18156351)×1 | 9 | 2.66 | II+III | Refused | 46,XY | Bilateral subependymal cysts (Left, 2.3×0.8cm; Right, 2.2×0.9cm), advanced maternal age | Yes | Eutocia | Male | 2019/6/20 | 39 | 2900 | 49 | 1 months: L=54.5cm(<Mean); W=4.4kg(<Mean); HC=38.5cm(>+1sd) 3 months: L=62cm(>Mean); W=6.5kg(>Mean); HC=40cm(<Mean) 12 months: L=75.5cm(<Mean); W=10.1kg(>Mean) 24 months: L=88.5cm(>Mean); W=13.2kg(>Mean) 30 months: L=94cm(>Mean); W=13.8kg(>Mean); HC=49.5cm(>Mean) 42 months: L=99cm(<Mean); W=15kg(<Mean); HC=50cm(>Mean) 57 months: L=110.3cm(>Mean); W=23kg(>+1sd) | Full-term delivery and AGA, Well survivor |
| 17 | 27 | UCB | arr[GRCh37] 16p13.11(15052746-16688008)×1 | 11 | 1.64 | I+II | Refused | 46,XX | Dilated renal pelvis (0.5cm), lateral ventricle broadening (1.0cm) | Yes | Eutocia | Female | 2019/8/27 | 39 | 3560 | 50 | 1 months: L=55.2cm(>Mean); W=4.25kg(>Mean) 6 months: L=68cm(=+1sd); W=7.8kg(>Mean); HC=40.5cm(<-1sd) 12 months: L=76cm(>Mean); W=9.9kg(>Mean); HC=44cm(<Mean) 24 months: L=82cm(<-1sd); W=12kg(>Mean); HC=46cm(<Mean) 36 months: L=97cm(>Mean); W=14.8kg(>Mean); HC=48cm(<Mean) 44 months: L=102cm(>Mean); W=16.5kg(>Mean) 56 months: L=110cm(>Mean); W=18.5kg(>Mean) 61 months: L=113cm(>Mean); W=17.85kg(>Mean); HC=50cm | Full-term delivery and AGA, Well survivor |
| 18 | 29 | AF | arr[GRCh37] 16p13.11(15052746-16303388)×1 | 10 | 1.25 | I+II | Maternal | 46,XN | High-risk screening for trisomy 21 | No | TP |  |  |  |  |  |  |  |
| 19 | 30 | AF | arr[GRCh37] 16p13.11(15052746-16303388)×1 | 10 | 1.25 | I+II | Refused | 46,XY | Left renal agenesis, left ectopic kidney with complete dilation of left ureter, DCDA | Yes | Cesarean | Male | 2020/3/5 | 37 | 3180 | 52 | 1 months: L=56cm(>Mean); W=5.5kg(>+1sd); HC=38.1cm(>Mean) 3 months: L=62cm(>Mean); W=7.1kg(>Mean); HC=40.8cm(>Mean) 12 months: L=77.5cm(>+1sd); W=11.2kg(>+1sd); HC=46cm(>Mean) 24 months: L=89cm(>Mean); W=14kg(>+1sd); HC=49cm(>Mean) 38 months: L=99cm(>Mean); W=16kg(>Mean); HC=50.4cm(>Mean) 49 months: L=107cm(>Mean); W=17.9kg(>Mean) | Full-term delivery and AGA, Well survivor |
| 20 | 36 | AF | arr[GRCh37] 16p13.11(15052746-16303388)×1 | 10 | 1.25 | I+II | denovo | 46,XN | Right choroid plexus cysts, advanced maternal age | Yes | TP |  |  |  |  |  |  |  |
| 21 | 40 | AF | arr[GRCh37] 16p13.11(15052746-16303388)×1 | 10 | 1.25 | I+II | Refused | 46,XY | Bilateral choroid plexus cysts, advanced maternal age | Yes | Eutocia | Male | 2020/8/13 | 39 | 3380 | 54 | 1 months: L=57.5cm(>+1sd); W=4.8kg(>Mean); HC=36.5cm(<Mean) 3 months: L=62.5cm(>Mean); W=6.6kg(>Mean); HC=41cm(>Mean) 12 months: L=78cm(>Mean); W=9kg(<Mean); HC=44.3cm(<-1sd) 24 months: L=88.2cm(>Mean); W=13kg(>Mean); HC=46.5cm(<-1sd) 30 months: L=92.3cm(>Mean); W=12.8kg(<Mean); HC=46.5cm(<-1sd) 34 months: L=97cm(>Mean); W=13.3kg(<Mean) 45 months: L=101.5cm(<Mean); W=14.6kg(<Mean) | Full-term delivery and AGA, Well survivor |
| 22 | 26 | VS | arr[GRCh37] 16p13.11p12.3(15493046-18156351)×1 | 9 | 2.66 | II+III | Refused | 46,XN | Thickened nuchal translucency (3.1mm), parent carried β-thalassemia gene | Yes | TP |  |  |  |  |  |  | Thalassemia result, β43/β17, Fetus with severe β-thalassemia |
| 23 | 35 | AF | arr[GRCh37] 16p13.11(15052746-16303388)×1 | 10 | 1.25 | I+II | Refused | 46,XN | Fast heart rhythm (170bpm), advanced maternal age, HSV-II/B19 | Yes | TP |  |  |  |  |  |  |  |
| 24 | 39 | AF | arr[GRCh37] 16p13.11p12.3(15493046-18156351)×1 | 9 | 2.66 | II+III | Refused | 46,XY | Single umbilical artery, parent carried α-thalassemia gene, advanced maternal age, MCDA | Yes | Cesarean | Male | 2020/12/14 | 38 | 2550 | 46 | 1 months: L=51.5cm(<-1sd); W=3.1kg(<-2sd); HC=35.5cm(<-1sd) 3 months: L=58cm(<-1sd); W=5.38kg(<-1sd); HC=39.1cm(<-1sd) 12 months: L=72cm(<-1sd); W=8.5kg(<-1sd); HC=44.5cm(<-1sd) 17 months: L=78cm(<-1sd); W=8.5kg(<-2sd); HC=46cm(<Mean) 33 months: L=91cm(<Mean); W=11.5kg(<-1sd) | Full-term delivery and SGA, Well survivor |
| 25 | 39 | AF | arr[GRCh37] 16p13.11p12.3(15493046-18156351)×1 | 9 | 2.66 | II+III | Refused | 46,XY | Parent carried α-thalassemia gene, advanced maternal age, MCDA | No | Cesarean | Male | 2020/12/14 | 38 | 2000 | 45 | 1 months: L=47.5cm(<-3sd); W=2.5kg(<-3sd); HC=34cm(<-2sd) 3 months: L=55cm(<-3sd); W=4.54kg(<-2sd); HC=37.2cm(<-2sd) 12 months: L=70cm(<-2sd); W=7.5kg(<-2sd); HC=43cm(<-2sd) 17 months: L=76cm(=-2sd); W=7.5kg(<-3sd); HC=45cm(<-1sd) 33 months: L=90cm(<-1sd); W=10.6kg(<-2sd) | Full-term delivery and SGA, Low birth weight infants (1500-2499g), Well survivor |
| 26 | 33 | UCB | arr[GRCh37] 16p13.11(15849059-16272508)×1 | 4 (NO NDE1) | 0.42 | II | Refused | 46,XY | Left upper abdominal mixed mass (5.7×3.8×3.2cm) | Yes | Cesarean | Male | 2020/10/3 | 38 | 3490 | 51 | 1 months: L=55cm(>Mean); W=5kg(>Mean); HC=37cm(<Mean) 6 months: L=67.5cm(<Mean); W=6.7kg(<-1sd); HC=43cm(<Mean) 12 months: L=71.5cm(<-1sd); W=9.3kg(<Mean); HC=45cm(<Mean) 17 months: L=83cm(>Mean); W=10.8kg(>Mean); HC=48cm(>Mean) 36 months: L=91.2cm(<-1sd); W=11.5kg(<-1sd); HC=50cm(>Mean)  47 months: L=99cm(<Mean); W=14.2kg(<-1sd) | Full-term delivery and AGA, Conjunctivitis, Bilateral testicular hydrocele, Retroperitoneal mature cystic teratoma, Mild anemia, Intestinal adhesions |
| 27 | 27 | AF | arr[GRCh37] 16p13.11(15052746-16303388)×1 | 10 | 1.25 | I+II | denovo | 46,XN | Adverse pregnancy history | No | TP |  |  |  |  |  |  |  |
| 28 | 30 | AF | arr[GRCh37] 16p13.11(15052746_16303388)×1 | 10 | 1.25 | I+II | Maternal | 46,XX | High-risk screening for trisomy 21 | No | Cesarean | Female | 2021/3/11 | 41 | 3250 | 50 | 1 months: L=55cm(>Mean); W=4.45kg(>Mean); HC=35.5cm(<Mean) 3 months: L=58.8cm(<Mean); W=5.6kg(<Mean); HC=38.5cm(<Mean) 12 months: L=68cm(<-2sd); W=8.1kg(<Mean); HC=43.5cm(=-1sd) 24 months: L=80cm(=-2sd); W=10kg(<-1sd); HC=45.5cm(<-1sd) 36 months: L=88cm(<-1sd); W=12kg(<-1sd); HC=47cm(<-1sd) 41 months: L=88.5cm(<-2sd); W=11.8kg(<-1sd) | Full-term delivery and AGA, Well survivor |
| 29 | 29 | AF | arr[GRCh37] 16p13.11(15052746_16303388)×1 | 10 | 1.25 | I+II | denovo | 46,XN | Left echogenic intracardiac focus, single umbilical artery, mild pulmonary regurgitation, mild pulmonary stenosis | Yes | TP |  |  |  |  |  |  |  |
| 30 | 38 | AF | arr[GRCh37] 16p13.11p12.3(15493046_18082349)×1 | 9 | 2.59 | II+III | Refused | 46,XN | Advanced maternal age | No | TP |  |  |  |  |  |  |  |
| 31 | 41 | AF | arr[GRCh37] 16p13.11(15052746_16688008)×1 | 11 | 1.64 | I+II | Paternal | 46,XY | Right echogenic intracardiac focus (0.2×0.1cm), advanced maternal age | Yes | Eutocia | Male | 2021/7/6 | 40 | 3510 | 53 | 1 months: L=57.5cm(>+1sd); W=4.5kg(=Mean); HC=38cm(>Mean) 3 months: L=63cm(>Mean); W=6.5kg(>Mean); HC=41cm(>Mean) 12 months: L=75cm(<Mean); W=8.7kg(<Mean); HC=46cm(<Mean) 18 months: L=83cm(>Mean); W=10.5kg(<Mean); HC=47cm(<Mean) 30 months: L=92cm(>Mean); W=14kg(>Mean); HC=48cm(<Mean) 37 months: L=95cm(<Mean); W=13.8kg(<Mean) | W=3510g, L=53.0cm, HC=33cm, CC=33.5cm, Full-term delivery and AGA, CHD, Second foramen atrial septal defect (type II), Venous sinus defect, Coronary sinus defect, Patent foramen ovale (2.5mm), Patent ductus arteriosus (2.0mm), Left eye nasolacrimal duct obstruction, Bilateral testicular hydrocele, Acute pharyngitis and bronchitis |
| 32 | 21 | VS | arr[GRCh37] 16p13.11(15052746_16303388)×1 | 10 | 1.25 | I+II | Refused | 46,XY | Adverse contact history in early pregnancy (X-ray) | No | Eutocia | Male | 2021/8/29 | 38 | 2900 | 50 | 1 months: L=54cm(<Mean); W=4.3kg(<Mean); HC=35.5cm(<-1sd) 3 months: L=62.5cm(>Mean); W=6.65kg(>Mean); HC=40.15cm(<Mean) 12 months: L=73cm(<-1sd); W=8.9kg(<Mean); HC=44cm(<-1sd) 18 months: L=82cm(<Mean); W=11.5kg(>Mean); HC=45.5cm(<-1sd) 30 months: L=94cm(>Mean); W=15kg(=+1sd); HC=49cm(>Mean) 36 months: L=95cm(<Mean); W=15kg(>Mean); HC=50cm(>Mean) | Full-term delivery and AGA, Yellow dyed complexion, Well survivor |
| 33 | 28 | AF | arr[GRCh37] 16p13.11(15052746_16303388)×1 | 10 | 1.25 | I+II | Maternal | 46,XX | Bilateral choroid plexus cysts, high-risk screening for trisomy 21 | Yes | Cesarean | Female | 2021/9/19 | 40 | 3200 | 50 | 1 months: L=54.2cm(>Mean); W=3.7kg(<Mean); HC=36.1cm(<Mean) 3 months: L=58.5cm(<Mean); W=5.1kg(<-1sd); HC=38.7cm(<Mean) 12 months: L=72.5cm(<Mean); W=7.4kg(<-1sd); HC=44.7cm(<Mean) 24 months: L=81.5cm(<-1sd); W=9.1kg(<-1sd); HC=46.5cm(<Mean) 30 months: L=85.5cm(<-1sd); W=10.1kg(<-1sd); HC=47cm(<Mean) 34 months: L=89.5cm(<-1sd); W=9.9kg(<-2sd) 36 months: L=93cm(<Mean); W=10.6kg(<-2sd); HC=48cm(<Mean) | Full-term delivery and AGA, Well survivor |
| 34 | 35 | AF | arr[GRCh37] 16p13.11(15052746_16303388)×1 | 10 | 1.25 | I+II | Refused | 46,XX | Permanent left superior vena cava, advanced maternal age | Yes | Cesarean | Female | 2021/7/23 | 38 | 2550 | 48 | 1 months: L=52cm(<Mean); W=3.6kg(=-1sd) 3 months: L=60cm(>Mean); W=5.7kg(<Mean); HC=38.5cm(<Mean) 12 months: L=74cm(=Mean); W=8.8kg(<Mean); HC=45.5cm(>Mean) 18 months: L=78cm(<Mean); W=9.5kg(<Mean); HC=46.5cm(>Mean) 30 months: L=85cm(<-1sd); W=13.8kg(>Mean); HC=48cm(>Mean) | Full-term delivery and SGA, Well survivor |
| 35 | 29 | AF | arr[GRCh37] 16p13.11(15052746_16303388)×1 | 10 | 1.25 | I+II | Maternal | 46,XN | Parent carried α-thalassemia gene | No | TP |  |  |  |  |  |  | Thalassemia result, -α4.2/--, Fetus with thalassemia intermedia |
| 36 | 23 | UCB | arr[GRCh37] 16p13.11(15052746_16303388)×1 | 10 | 1.25 | I+II | Paternal | 46,XX | Short femur length | Yes | Eutocia | Female | 2021/10/11 | 41 | 3690 | 50 | 1 months: L=53.8cm(>Mean); W=4.05kg(<Mean); HC=35.2cm(<-1sd) 2 months: L=58.1cm(>Mean); W=6kg(>+1sd); HC=38cm(<Mean) 12 months: L=72.8cm(<Mean); W=8.7kg(<Mean); HC=44.2cm(<Mean) 24 months: L=82cm(<-1sd); W=11kg(<Mean); HC=47cm(<Mean) 30 months: L=87cm(<-1sd); W=11.7kg(<Mean); HC=47.3cm(<Mean) 32 months: L=95cm(>Mean); W=11.9kg(<Mean) | Full-term delivery and AGA, LDD at 1 year, Scattered erythema and wheals on both lower limbs and urticaria at 2 years |
| 37 | 36 | AF | arr[GRCh37] 16p13.11p12.3(15514392_18156351)×1 | 8 | 2.64 | II+III | Refused | 46,XX | Adverse pregnancy history, advanced maternal age | No | Eutocia | Female | 2022/1/18 | 40 | 3500 | 51 | 1 months: L=56cm(>+1sd); W=5kg(>+1sd); HC=37cm(>Mean) 3 months: L=61.8cm(>Mean); W=6.1kg(>Mean); HC=40.2cm(>Mean) 12 months: L=78.3cm(>+1sd); W=9.41kg(>Mean); HC=44.8cm(<Mean) 24 months: L=85.8cm(<Mean); W=11.3kg(<Mean); HC=46.7cm(<Mean) 26 months: L=87cm(<Mean); W=12kg(>Mean) | Full-term delivery and AGA, Well survivor |
| 38 | 31 | AF | arr[GRCh37] 16p13.11(15052746_16303388)×1 | 10 | 1.25 | I+II | Refused | 46,XX | Choroid plexus cysts, adverse pregnancy history | Yes | Eutocia | Female | 2022/2/6 | 37 | 2860 | 50 | 1 months: L=51cm(<-1sd); W=3.3kg(<-1sd); HC=34cm(<-2sd) 3 months: L=59cm(<Mean); W=5kg(<-1sd); HC=38cm(<-1sd) 8 months: L=68.5cm(<Mean); W=7.3kg(<Mean); HC=41.5cm(<-1sd) 12 months: L=71cm(<-1sd); W=7.8kg(<-1sd); HC=44cm(<Mean) 27 months: L=86cm(<Mean); W=10.6kg(<-1sd); HC=46.5cm(<Mean) | W=2860g, L=50.0cm, HC=31.0cm, CC=32.0cm, Full-term delivery and AGA, Yellow dyed complexion, Influenza, Well survivor |
| 39 | 34 | UCB | arr[GRCh37] 16p13.11(15052746_16303388)×1 | 10 | 1.25 | I+II | Refused | 46,XX | Thickened placenta, parent carried α-thalassemia gene | Yes | Eutocia | Female | 2022/1/31 | 38 | 3200 | 50 | 1 months: L=53cm(<Mean); W=3.9kg(<Mean); HC=35.5cm(<Mean) 3 months: L=60.4cm(>Mean); W=5.7kg(<Mean); HC=38.5cm(<Mean) 12 months: L=76.5cm(>Mean); W=9.1kg(<Mean); HC=45.8cm(>Mean) 18 months: L=79cm(<Mean); W=9.7kg(<Mean); HC=45.8cm(<Mean) | Full-term delivery and AGA, Upper respiratory infection, Well survivor |
| 40 | 28 | UCB | arr[GRCh37] 16p13.11(15493046_16279283)×1 | 7 | 0.79 | II | Refused | 46,XN | Left echogenic intracardiac focus | Yes | TP |  |  |  |  |  |  |  |
| 41 | 24 | AF | arr[GRCh37] 16p13.11(15052746_16289532)×1 | 10 | 1.24 | I+II | Refused | 46,XY | Dilated renal pelvis (Left, 0.55cm; Right, 0.41cm) | Yes | Eutocia | Male | 2022/3/31 | 40 | 3240 | 50 | 1 months: L=57.2cm(>+1sd); W=5kg(>Mean); HC=38cm(>Mean) 3 months: L=62.2cm(>Mean); W=6.2kg(<Mean); HC=40cm(<Mean) 8 months: L=69cm(<Mean); W=8.1kg(<Mean); HC=43.5cm(<Mean) 12 months: L=73cm(<-1sd); W=9kg(<Mean); HC=44cm(<-1sd) 24 months: L=86cm(<Mean); W=11.5kg(<Mean); HC=47.5cm(<Mean) 30 months: L=90cm(<Mean); W=11.5kg(<-1sd); HC=48.2cm(<Mean) | Full-term delivery and AGA, Torticollis, Eczema, Skin rash, Dryness and itching in face (Dermatitis) at 5 months |
| 42 | 30 | VS | arr[GRCh37] 16p13.11(15052746_16786098)×1 | 11 | 1.73 | I+II | Maternal | 46,XN | Parent carried α-thalassemia gene, maternal 16p13.11 deletion, adverse pregnancy history (16p13.11del) | No | TP |  |  |  |  |  |  | Thalassemia result, αα/αα |
| 43 | 29 | AF | arr[GRCh37] 16p13.11p12.3(15514392_18886254)×1 | 12 | 3.37 | II+III | Maternal | 46,XY | Parent carried α-thalassemia gene, adverse pregnancy history (G3P3, MDD) | No | Eutocia | Male | 2023/4/8 | 39 | 3100 | 50 | 1 months: L=54cm(<Mean); W=4.2kg(<Mean); HC=36.6cm(<Mean) 2 months: L=60.5cm(>+1sd); W=6.4kg(>+1sd); HC=39cm(<Mean) 8 months: L=71cm(>Mean); W=8.25kg(<Mean) 12 months: L=74cm(<Mean); W=9.15kg(<Mean); HC=44.5cm(<-1sd) 18 months: L=80cm(<Mean); W=9.75kg(<-1sd); HC=46cm(=-1sd) | W=3100g, L=50.0cm, HC=33.0cm, CC=32.0cm, Full-term delivery and AGA, A caput succedaneum about 4×3cm, Neonatal pathological jaundice and hyperbilirubinemia, Torticollis at 4 months, MDD at 5 months, Left cerumen embolism, GDD at 1 years, MRI of head showed no obvious abnormalities, provided early education and follow-up examination |
| 44 | 38 | AF | arr[GRCh37] 16p13.11(15052746_16303388)×1 | 10 | 1.25 | I+II | denovo | 46,XY | Adverse pregnancy history (CHD), advanced maternal age | No | Eutocia | Male | 2023/7/22 | 40 | 3235 | 50 | 1 months: L=56cm(>Mean); W=4.4kg(<Mean);  3 months: L=63cm(>Mean); W=6.5kg(>Mean); HC=41.5cm(>Mean) 6 months: L=70cm(>+1sd); W=8kg(>Mean); HC=43cm(<Mean) 8 months: L=73cm(>+1sd); W=8.8kg(>Mean); HC=45cm(>Mean) 12 months: L=75cm(<Mean); W=9.3kg(<Mean); HC=46.2cm(>Mean) | Full-term delivery and AGA, Well survivor |
| 45 | 27 | AF | arr[GRCh37] 16p13.11(15129970_16279283)×1 | 10 | 1.15 | I+II | Maternal | 46,XY | Maternal 16p13.11 deletion | No | Eutocia | Male | 2023/9/2 | 40 | 3230 | 53 | 1 months: L=55.6cm(>Mean); W=4.8kg(>Mean);  3 months: L=62.4cm(>Mean); W=7.1kg(>Mean); HC=40.5cm(=Mean) 6 months: L=68.8cm(>Mean); W=8.5kg(>Mean); HC=42.5cm(<Mean) 8 months: L=70.3cm(<Mean); W=9.1kg(>Mean); HC=43.6cm(<Mean) 12 months: L=78.3cm(>+1sd); W=10.3kg(>Mean); HC=45cm(<Mean) | Full-term delivery and AGA, Well survivor |
| 46 | 22 | VS | arr[GRCh37] 16p13.11p12.3(15493046_18156351)×1 | 9 | 2.66 | II+III | Maternal | 46,XX | Small-sized kidneys (Left, 3.1×1.6cm; Right, 2.7×1.5cm), FGR and IUGR, parent carried α-thalassemia gene | Yes | Eutocia | Female | 2023/10/7 | 38 | 2650 | 48 | 1 months: L=52cm(<Mean); W=3.9kg(<Mean) 2 months: L=56cm(<Mean); W=4.6kg(<Mean); HC=37cm(=-1sd) 4 months: L=58cm(<-1sd); W=5.2kg(<-1sd) 6 months: L=64cm(<Mean); W=6kg(<-1sd) 7 months: L=65cm(=-1sd); W=6.55kg(<-1sd); HC=40cm(<-2sd) 8 months: L=66cm(<-1sd); W=6.8kg(<-1sd); HC=41cm(<-1sd) 12 months: L=71.5cm(<Mean); W=7.6kg(<-1sd); HC=42.2cm(=-2sd) | W=2650g, L=48.0cm, HC=32.0cm, CC=32.0cm, Full-term delivery and SGA, A caput succedaneum about 4×3cm, Jaundice, α-thalassemia intermedia (Hb H), Well survivor, Mother with facial white spots pigmentation and tinea versicolor (Case 217) |
| 47 | 36 | UCB | arr[GRCh37] 16p13.12p13.11 (14760734_16688008)×1 | 15 | 1.93 | I+II | Refused | 46,XX | Cavum septum pellucidum not displayed, colon dilatation (1.57cm), advanced maternal age, DCDA | Yes | Eutocia | Female | 2023/6/25 | 37 | 2070 | 47 | 1 months: L=49cm(<-2sd); W=3.35kg(<-1sd) 3 months: L=59cm(<Mean); W=6kg(>Mean); HC=40cm(>Mean) 6 months: L=66cm(>Mean); W=7.2kg(<Mean); HC=42cm(<Mean) 8 months: L=70cm(>Mean); W=8.2kg(>Mean); HC=43cm(<Mean) 12 months: L=70.5cm(<-1sd); W=8.3kg(<Mean); HC=44cm(<Mean) | Full-term delivery and SGA, Low birth weight infants (1500-2499g), Well survivor |
| 48 | 29 | AF | arr[GRCh37]16p13.11(15052746_16277685)×1 | 10 | 1.22 | I+II | Refused | 46,XN | Tetralogy of Fallot: ventricular septal defect (0.22cm), pulmonary stenosis (0.12cm), aortic ride across | Yes | TP |  |  |  |  |  |  |  |
| 49 | 39 | AF | arr[GRCh37] 16p13.11(15052746_16303388)×1 | 10 | 1.25 | I+II | Maternal | 46,XX | Permanent left superior vena cava, advanced maternal age | Yes | Eutocia | Female | 2023/11/14 | 40 | 3400 | 50 | 1 months: L=53cm(<Mean); W=4.4kg(>Mean) 3 months: L=58cm(<Mean); W=5.5kg(<Mean); HC=39cm(<Mean) 6 months: L=64cm(<Mean); W=6.8kg(<Mean); HC=41cm(<Mean) 8 months: L=66cm(<-1sd); W=7.7kg(<Mean); HC=41cm(<-1sd) 12 months: L=72.5cm(<Mean); W=9.1kg(>Mean); HC=43.5cm(=-1sd) | Full-term delivery and AGA, Well survivor |
| 50 | 37 | VS | arr[GRCh37] 16p13.11p12.3(15493046_18156351)×1 | 9 | 2.66 | II+III | Refused | 46,XX | Thickened nuchal translucency (2.7mm), advanced maternal age | Yes | Eutocia | Female | 2024/2/22 | 39 | 3850 | 52 | 1 months: L=57cm(>+1sd); W=5kg(>+1sd) 3 months: L=62.5cm(>+1sd); W=6kg(>Mean); HC=40cm(>Mean) 6 months: L=70cm(>+1sd); W=7kg(<Mean); HC=41cm(<Mean)  8 months: L=72.5cm(>+1sd); W=8kg(>Mean); HC=43cm(<Mean) | Full-term delivery and AGA, Well survivor |
| 51 | 41 | AF | arr[GRCh37] 16p13.11(15052746_16303388)×1 | 10 | 1.25 | I+II | Refused | 46,XN | Advanced maternal age | No | TP |  |  |  |  |  |  |  |
| 52 | 20 | UCB | arr[GRCh37] 16p13.11p12.3(15443148_18851008)×1 | 13 | 3.41 | II+III | Refused | 46,XX | Pregnant women with intellectual disability | No | Eutocia | Female | 2024/4/26 | 33 | 1950 | 46 | 1 months: L=53cm(<Mean); W=3.3kg(<-1sd) 3 months: L=55.6cm(<Mean); W=5kg(<Mean); HC=37.5cm(<Mean) 6 months: L=65cm(<Mean); W=6.5kg(=-1sd); HC=41cm(<Mean) | Preterm delivery and AGA, Low birth weight infants (1500-2499g), Well survivor |
| 53 | 37 | AF | arr[GRCh37] 16p13.11p12.3(15239631_18886254)×1 | 13 | 3.65 | II+III | Paternal | 46,XY | Advanced maternal age | No | Eutocia | Male | 2024/7/30 | 39 | 3200 | 51 | 1 months: L=53.5cm(<Mean); W=4.5kg(=Mean)  3 months: L=60.6cm(<Mean); W=6.45kg(>Mean); HC=40.3cm(<Mean) | Full-term delivery and AGA, Well survivor |
| 54 | 33 | AF | arr[GRCh37] 16p13.11(15052746_16303388)×1 | 10 | 1.25 | I+II | Refused | 46,XN | Adverse pregnancy history (Trisomy 21) | No | TP |  |  |  |  |  |  |  |
| 55 | 29 | VS | arr[GRCh37] 16p13.12p13.11(14718095_16726571)×1 | 16 | 2.01 | I+II | Paternal | 46,XX | Parent carried α-thalassemia gene, adverse pregnancy history (G3P0) | No | Cesarean | Female | 2025/1/3 | 38 | 3060 | 51 | / | W=3060g, L=51.0cm, HC=34.0cm, CC=34.0cm, Full-term delivery and AGA, Thalassemia result, αα/--, Fetus with mild thalassemia, Well survivor |
| 56 | 32 | VS | arr[GRCh37] 16p13.11p12.3(15493046_18886254)×1 | 13 | 3.39 | II+III | Refused | 46,XN | Parent carried α-thalassemia gene | No | TP |  |  |  |  |  |  | Fetus with severe α-thalassemia |
| 57 | 28 | AF | arr (18)×3, arr[GRCh37] 16p13.11(15052746_16303388)×1 | 10 | 1.25 | I+II | Refused | 47,XN,+18 | High-risk screening for trisomy 18 | No | TP |  |  |  |  |  |  | Fetus with trisomy 18 |
| 58 | 34 | AF | arr[GRCh37] 1q31.3(194955350_196651832)×3, 16p13.11(15052746_16277685)×1 | 10 | 1.22 | I+II | Maternal | 46,XY | Medication history in early pregnancy | No | Eutocia | Male | 2023/3/17 | 40 | 3330 | 50 | 1 months: L=56.5cm(>Mean); W=4.5kg(=Mean) 3 months: L=62cm(>+1sd); W=5.98kg(>Mean); HC=40cm(>Mean) 12 months: L=71.5cm(<-1sd); W=9kg(<Mean); HC=45cm(<Mean) 18 months: L=75cm(<-2sd); W=9.5kg(<-1sd); HC=46cm(=-1sd) | Full-term delivery and AGA, Fetus with paternal 1q31.3 duplication, Umbilicalhernia (2*1.5cm) and constipation at 1 month |
| 59 | 37 | AF | arr(21)×3, arr[GRCh37] 16p13.11p12.3(15514392_18156351)×1 | 8 | 2.64 | II+III | Refused | 47,XN,+21 | Abnormal NIPT results (Trisomy 21), adverse pregnancy history | No | TP |  |  |  |  |  |  | Fetus with trisomy 21 |
| 60 | 29 | UCB | arr[GRCh37] 11p15.3(10918279_12662120)×3, 16p13.11(15052746_16303388)×1 | 10 | 1.25 | I+II | Paternal | 46,XX | Polyhydramnios, bilateral dilated renal pelvis (Left, 0.43cm; Right, 0.52cm) | Yes | Cesarean | Female | 2023/2/9 | 38 | 2500 | 48 | 1 months: L=51.5cm(<-1sd); W=3.3kg(<-1sd) 3 months: L=57.6cm(<-1sd); W=5.3kg(<Mean); HC=39.5cm(=Mean) 12 months: L=70cm(<-1sd); W=7.35kg(<-1sd); HC=43cm(<-1sd) 18 months: L=75cm(<-1sd); W=8.3kg(<-1sd); HC=45.5cm(<Mean) | Full-term delivery and SGA, Fetus with paternal 11p15.3 duplication, Well survivor |
| VS, villus sampling; AF, amniotic fluid; UCB, umbilical cord blood; TP, termination of pregnancy; FGR, fetal growth restriction; IUGR, intrauterine growth retardation; CHD, congenital heart disease; NEC, neonatal necrotizing enterocolitis; SGA, small for gestational age; AGA, appropriate for gestational age; LGA, large for gestational age; W, weight; L, length; HC, head circumference; CC, chest circumference; Well survivor, the level of growth and development in line with peers; DD, global developmental delay; GDD, growth developmental delay; MDD, motor developmental delay; LDD, language developmental delay; DCDA, dichorionic diamniotic twins; MCDA, monochorionic diamniotic twins. Case 24 and 25 are twins. Case 35 and 42 have the same mother (Case 210). These 9 fetuses with abnormal postnatal phenotypes in outcome follow-up are highlighted in pink. These 4 fetuses with additional genomic abnormalities are depicted in green. | | | | | | | | | | | | | | | | | | |
|  |  |  |  |  |  |  |  |  |  |  |  |  |  |  |  |  |  |  |
|  |  |  |  |  |  |  |  |  |  |  |  |  |  |  |  |  |  |  |
|  |  |  |  |  |  |  |  |  |  |  |  |  |  |  |  |  |  |  |

| Table S2. CMA, prenatal diagnosis indications and outcome follow-up of 141 fetuses with 16p13.11 duplication. | | | | | | | | | | | | | | | | | | |
| --- | --- | --- | --- | --- | --- | --- | --- | --- | --- | --- | --- | --- | --- | --- | --- | --- | --- | --- |
| Case index | Maternal age (y) | Sample type | CMA | OMIM gene | Size (Mb) | Interval | Inheritance | Chromosome karyotype | Prenatal diagnosis indications | Abnormal ultrasound | Pregnancy outcome | Fetus gender | Delivery date | Delivery week (w) | Birth weight (g) | Birth length (cm) | Physical examination | Outcome follow-up |
| 61 | 34 | UCB | arr[GRCh37] 16p13.11(14758345-16279283)×3 | 14 | 1.52 | I+II | Refused | 46,XN | Cleft lip and palate, adverse pregnancy history | Yes | TP |  |  |  |  |  |  |  |
| 62 | 31 | UCB | arr[GRCh37] 16p13.11p12.3(15239631-18799615)×3 | 11 | 3.56 | II+III | Refused | 46,XN | Adverse pregnancy history (cerebral palsy) | No | TP |  |  |  |  |  |  |  |
| 63 | 21 | AF | arr[GRCh37] 16p13.11(15127986-16289532)×3 | 10 | 1.16 | I+II | Refused | 46,XY,22pstk+ | High-risk screening for trisomy 21 | No | Eutocia | Male | 2016/5/15 | 40 | 3000 | 50 | / | Full-term delivery and AGA, Well survivor |
| 64 | 24 | UCB | arr[GRCh37] 16p13.11p12.3(15499057-18799615)×3 | 11 | 3.3 | II+III | Refused | 46,XX | Oligohydramnios, hyperechogenic bowel, small magenblase (1.2×0.3cm) and thickened placenta | Yes | Cesarean | Female | 2016/3/9 | 31 | 1340 | 40 | 3 months: L=53cm(=-2sd); W=3.8kg(<-2sd); HC=35.3cm(<-2sd) 12 months: L=72.5cm(<Mean); W=8.1kg(<Mean); HC=44cm(<Mean) 24 months: L=84.5cm(<Mean); W=10.6kg(<Mean); HC=47cm(<Mean) 32 months: L=90cm(<Mean); W=12.6kg(<Mean); HC=49cm(>Mean) | Preterm delivery and AGA, Low birth weight infants (1500-2499g), Well survivor |
| 65 | 32 | VS | arr[GRCh37] 16p13.11(15127986-16308351)×3 | 10 | 1.18 | I+II | Paternal | 46,XX | Adverse pregnancy history | No | Eutocia | Female | 2016/7/29 | 37 | 2490 | 49 | 3 months: L=59cm(>Mean); W=5.5kg(>Mean); HC=37.6cm(<Mean) 12 months: L=71.5cm(<Mean); W=7.9kg(=-1sd); HC=44cm(<Mean) 36 months: L=88.8cm(<-1sd); W=10.6kg(<-2sd); HC=46.6cm(<-1sd) 48 months: L=97.8cm(<-1sd); W=12.5kg(<-1sd); HC=48cm(<Mean) 60 months: L=103.5cm(<-1sd); W=14.8kg(<-1sd); HC=48.3cm 72 months: L=113cm(<Mean); W=15.75kg(<-1sd); HC=53cm(>Mean) | Full-term delivery and SGA, Low birth weight infants (1500-2499g), Well survivor |
| 66 | 29 | UCB | arr[GRCh37] 16p13.11(15052746-16308351)×3 | 10 | 1.26 | I+II | Refused | 46,XX | Fetal arhythmia (Atrial rate, 140bpm; Ventricular rate, 65bpm) | Yes | Cesarean | Female | 2016/7/5 | 37 | 2700 | 47 | 3 months: L=62cm(>+1sd); W=5.9kg(>Mean); HC=38.5cm(<Mean) 51 months: L=105.5cm(>Mean); W=16kg(<Mean) 63 months: L=110cm(=Mean); W=18.4kg(>Mean) | Full-term delivery and AGA, CHD, Atrioventricular block, Patent ductus arteriosus |
| 67 | 33 | VS | arr[GRCh37] 16p13.11(15127986-16308351)×3 | 10 | 1.18 | I+II | Refused | 46,XX | Thickened nuchal translucency, DCDA | Yes | Cesarean | Female | 2016/11/21 | 37 | 2380 | 46 | 1 months: L=49.5cm(<-2sd); W=3.4kg(<-1sd) 3 months: L=55cm(=-1sd); W=4.5kg(=-1sd); HC=38cm(<Mean) 12 months: L=69cm(<-1sd); W=7.2kg(<-1sd); HC=43.5cm(=-1sd) 24 months: L=81cm(<-1sd); W=9.5kg(<-1sd); HC=46cm(<Mean) 36 months: L=91cm(<-1sd); W=12.5kg(<Mean); HC=48.5cm(=Mean) 67 months: L=109.7cm(<Mean); W=21.4kg(>Mean); HC=51cm 77 months: L=114cm(<Mean); W=24.55kg(>+1sd) | Full-term delivery and SGA, Low birth weight infants (1500-2499g), Well survivor |
| 68 | 39 | UCB | arr[GRCh37] 16p13.11(15126890-16289532)×3 | 10 | 1.16 | I+II | Refused | 46,XY | Large head circumference (31cm), advanced maternal age | Yes | Cesarean | Male | 2016/9/16 | 39 | 3500 | 50 | 3 months: L=65cm(>+1sd); W=7.4kg(>+1sd); HC=43cm(>+2sd) 12 months: L=79cm(>+1sd); W=11.54kg(>+1sd); HC=47cm(>Mean) 24 months: L=93cm(>+1sd); W=14.5kg(>+1sd); HC=48.5cm(>Mean) 34 months: L=100cm(>+1sd); W=17.1kg(>+1sd) 45 months: L=106cm(>+1sd); W=18.5kg(>+1sd) 68 months: L=121cm(>+1sd); W=24.4kg(>+1sd) | W=3500g, L=50cm, HC=37cm, CC=37cm, Full-term delivery and AGA, Well survivor |
| 69 | 35 | UCB | arr[GRCh37] 16p13.11(15126890-16289532)×3 | 10 | 1.16 | I+II | Refused | 46,XX | Liquid dark area in the right upper fetal bladder (4.6×2.9×3.9cm) | Yes | Eutocia | Female | 2016/6/25 | 40 | 4240 | 54 | 1 months: L=53.5cm(<Mean); W=5.1kg(>+1sd) 3 months: L=62.3cm(>+1sd); W=6.8kg(>+1sd); HC=40.2cm(>Mean) 12 months: L=73.5cm(>Mean); W=7.8kg(<Mean); HC=44.2cm(<Mean) 24 months: L=85.2cm(<Mean); W=9.8kg(<-1sd); HC=47.5cm(>Mean) 36 months: L=93.2cm(<Mean); W=12.2kg(=-1sd); HC=48.5cm(=Mean) 48 months: L=104.5cm(>Mean); W=15kg(<Mean); HC=47.8cm(<-1sd) 72 months: L=119cm(>Mean); W=21.4kg(>Mean); HC=49.5cm | Full-term delivery and LGA, Patent foramen ovale, Mild tricuspid regurgitation, A cystic teratoma about 4×3cm in right lower abdomen |
| 70 | 27 | AF | arr[GRCh37] 16p13.11(15127986-16308351)×3 | 10 | 1.18 | I+II | Refused | 46,XX | High-risk screening for open neural tube defects (ONTD) | No | Eutocia | Female | 2017/1/13 | 40 | 3845 | 51 | 3 months: L=60.5cm(>Mean); W=5.1kg(<-1sd); HC=38.5cm(<Mean) 12 months: L=77.4cm(>+1sd); W=9.5kg(>Mean); HC=44cm(<Mean) 24 months: L=86cm(<Mean); W=12kg(>Mean); HC=46cm(<Mean) 43 months: L=99cm(<Mean); W=15kg(<Mean); HC=48.8cm(<Mean) 75 months: L=119cm(>Mean); W=23kg(>Mean) | Full-term delivery and AGA, Well survivor |
| 71 | 36 | AF | arr[GRCh37] 16p13.11(15140211-16308351)×3 | 10 | 1.17 | I+II | Refused | 46,XN | Oligohydramnios, FGR, advanced maternal age | Yes | TP |  |  |  |  |  |  |  |
| 72 | 28 | UCB | arr[GRCh37] 16p13.11(15052746-16308351)×3 | 10 | 1.26 | I+II | Refused | 46,XY | High-risk screening for trisomy 21 | No | Eutocia | Male | 2017/1/19 | 39 | 2950 | 50 | 1 months: L=54cm(<Mean); W=4.58kg(>Mean) | Full-term delivery and AGA, Well survivor |
| 73 | 26 | AF | arr[GRCh37] 16p13.11(15140211-16279283)×3 | 10 | 1.14 | I+II | Maternal | 46,XN | High-risk screening for trisomy 21 | No | TP |  |  |  |  |  |  |  |
| 74 | 36 | AF | arr[GRCh37] 16p13.11(15126890-16925005)×3 | 11 | 1.8 | I+II | Paternal | 46,XX | Abnormal NIPT results, advanced maternal age | No | Cesarean | Female | 2017/6/26 | 39 | 3960 | 51 | 1 months: L=57.5cm(>+1sd); W=4.5kg(>Mean) 3 months: L=60cm(>Mean); W=4.95kg(<-1sd); HC=40cm(>Mean) 12 months: L=73cm(<Mean); W=6.55kg(<-2sd); HC=44.6cm(<Mean) 36 months: L=92.8cm(<Mean); W=11.1kg(<-1sd); HC=47.8cm(<Mean) 74 months: L=112cm(<Mean); W=14.9kg(<-2sd) 82 months: L=117cm(<Mean); W=15.8kg(<-2sd) | Full-term delivery and LGA, Local pediatric assessment showed no significant abnormalities at 8 months, Follow-up showed cannot roll over and crawl at 9 months, GDD, LDD but normal motor development at 2 years and 4 months |
| 75 | 27 | UCB | arr[GRCh37] 16p13.11(15140211-16308351)×3 | 10 | 1.17 | I+II | Refused | 46,XY | Abnormal echo in the liver (2.1×1.1×1.7cm), enlarged cisterna magnal (1.19cm) | Yes | Eutocia | Male | 2017/7/25 | 38 | 3750 | 53 | 1 months: L=56cm(<Mean); W=4.5kg(=Mean) 3 months: L=66cm(>+2sd); W=7kg(>Mean); HC=40cm(<Mean) 72 months: L=113cm(<Mean); W=18.7kg(<Mean); HC=52cm(=Mean) | Full-term delivery and AGA, Well survivor |
| 76 | 32 | AF | arr[GRCh37] 16p13.11(15127986-16308531)×3 | 10 | 1.18 | I+II | Paternal | 46,XY | Oligohydramnios, parent carried α-thalassemia gene, DCDA | Yes | Eutocia | Male | 2017/9/30 | 34 | 1810 | 44 | 4 months: L=59.3cm(<-2sd); W=5.8kg(<-1sd); HC=39.1cm(<-2sd) 12 months: L=73.8cm(<Mean); W=9.6kg(=Mean); HC=46.3cm(>Mean) 24 months: L=88cm(>Mean); W=13kg(>Mean); HC=49cm(>Mean) 37 months: L=95cm(<Mean); W=16kg(>Mean); HC=49cm(<Mean) 71 months: L=115cm(<Mean); W=21kg(>Mean) 79 months: L=119.5cm(>Mean); W=22kg(>Mean) | W=1810g, L=44cm, HC=29cm, CC=27cm, Preterm delivery and SGA, Low birth weight infants (1500-2499g), NEC, Patent foramen ovale (2.0mm), Jaundice, No abnormalities in the nervous system and ophthalmological evaluation, Depigmented nevus and nevus flammeus in right lateral thigh, Diffused pinpoint sized skin rash in all over the body (Dermatitis), Angular cheilitis |
| 77 | 30 | AF | arr[GRCh37] 16p13.11(15127986-16308351)×3 | 10 | 1.18 | I+II | Refused | 46,XY | Paternal balanced chromosomal translocation | No | Cesarean | Male | 2018/2/14 | 37 | 3000 | 50 | 1 months: L=53.5cm(<Mean); W=4kg(<Mean) 3 months: L=60cm(<Mean); W=6.5kg(>Mean); HC=41cm(>Mean) 12 months: L=75cm(<Mean); W=9.2kg(<Mean); HC=48.6cm(=+2sd) 24 months: L=87cm(<Mean); W=12kg(<Mean); HC=51cm(>+1sd) 36 months: L=93cm(<Mean); W=14.1kg(<Mean); HC=52.2cm(>+1sd) 63 months: L=109cm(<Mean); W=17kg(=-1sd); HC=51cm(>Mean) 74 months: L=113cm(<Mean); W=18kg(<-1sd) | Full-term delivery and AGA, Well survivor |
| 78 | 28 | AF | arr[GRCh37] 16p13.11(15127986-16308351)×3 | 10 | 1.18 | I+II | denovo | 46,XX | Medication history in early pregnancy | No | Eutocia | Female | 2018/2/12 | 38 | 3310 | 51 | 1 months: L=52.8cm(<Mean); W=4.4kg(>Mean) 3 months: L=60cm(>Mean); W=6.1kg(>Mean); HC=39cm(<Mean) 12 months: L=73.2cm(<Mean); W=8.5kg(<Mean); HC=45.5cm(>Mean) 24 months: L=87cm(>Mean); W=11.5kg(=Mean); HC=47.5cm(>Mean) 36 months: L=98.6cm(>Mean); W=14kg(>Mean); HC=49cm(>Mean) 73 months: L=116.5cm(>Mean); W=20.3kg(>Mean) | Full-term delivery and AGA, Well survivor |
| 79 | 34 | VS | arr[GRCh37] 16p13.11(15052746-16308351)×3 | 10 | 1.26 | I+II | Paternal | 46,XX | Parent carried α-thalassemia gene | No | Cesarean | Female | 2018/4/28 | 39 | 3300 | 50 | 1 months: L=55cm(>Mean); W=4.6kg(>Mean) 3 months: L=62.5cm(>+1sd); W=6.8kg(>+1sd); HC=40cm(>Mean) 13 months: L=74.5cm(<Mean); W=9.3kg(>Mean); HC=46.5cm(<=+1sd) 23 months: L=87.5cm(>Mean); W=12.2kg(>Mean); HC=48cm(>Mean) 35 months: L=98cm(>Mean); W=14.4kg(>Mean); HC=50cm(>+1sd) 57 months: L=111cm(>Mean); W=20.4kg(>Mean) 73 years: L=121cm(>+1sd); W=24kg(>+1sd) | Full-term delivery and AGA, Well survivor |
| 80 | 37 | AF | arr[GRCh37] 16p13.11(15126890-16308351)×3 | 10 | 1.18 | I+II | Refused | 46,XY | Advanced maternal age | No | Cesarean | Male | 2018/3/22 | 38 | 3050 | 50 | 1 months: L=55cm(>Mean); W=4.5kg(=Mean) 3 months: L=62cm(>Mean); W=6kg(<Mean); HC=40cm(<Mean) 12 months: L=76.5cm(>Mean); W=9.2kg(<Mean); HC=46cm(<Mean) 25 months: L=89cm(>Mean); W=11.5kg(<Mean); HC=48cm(<Mean) 36 months: L=96cm(<Mean); W=14kg(<Mean); HC=47cm(<-1sd) 66 months: L=115cm(>Mean); W=18.5kg(<Mean) 73 months: L=118cm(>Mean); W=19.2kg(<Mean) | Full-term delivery and AGA, Well survivor |
| 81 | 31 | AF | arr[GRCh37] 16p13.11(14771789-16308351)×3 | 13 | 1.54 | I+II | Maternal | 46,XX | Left echogenic intracardiac focus (4×3mm), high-risk screening for trisomy 21 | Yes | Cesarean | Female | 2018/4/25 | 39 | 3750 | 51 | 1 months: L=56cm(>+1sd); W=4.9kg(>+1sd)  3 months: L=62cm(>+1sd); W=6.4kg(>Mean); HC=40.8cm(=+1sd) 12 months: L=74.5cm(>Mean); W=9.7kg(>Mean); HC=45.8cm(>Mean) 24 months: L=86cm(<Mean); W=11.4kg(<Mean); HC=47.2cm(=Mean) 36 months: L=96.5cm(>Mean); W=14kg(>Mean); HC=48.5cm(=Mean) 60 months: L=110.5cm(>Mean); W=16.4kg(<Mean) 73 months: L=122cm(>+1sd); W=22kg(>Mean); HC=52cm(=Mean) | Full-term delivery and AGA, Well survivor |
| 82 | 25 | AF | arr[GRCh37] 16p13.11(15052746-16289532)×3 | 10 | 1.24 | I+II | Maternal | / | High-risk screening for trisomy 21 | No | Eutocia | Female | 2018/6/29 | 38 | 2950 | 50 | 1 months: L=52.2cm(<Mean); W=4.6kg(>Mean) 3 months: L=60.8cm(>Mean); W=6.69kg(>+1sd); HC=40.5cm(>Mean)  12 months: L=73.2cm(<Mean); W=9.1kg(>Mean); HC=44.8cm(<Mean) 24 months: L=85cm(<Mean); W=12kg(>Mean); HC=47.2cm(=Mean) 36 months: L=95.5cm(>Mean); W=14.1kg(>Mean); HC=48.1cm(<Mean) 59 months: L=108cm(<Mean); W=19kg(>Mean) 70 months: L=114cm(>Mean); W=20.8kg(>Mean) | Full-term delivery and AGA, Well survivor |
| 83 | 24 | AF | arr[GRCh37] 16p13.11(15129970-16308351)×3 | 10 | 1.18 | I+II | denovo | 46,XN | Bilateral choroid plexus cysts (Left, 1.6×0.6cm; Right, 1.8×0.6cm), high-risk screening for trisomy 21 | Yes | TP |  |  |  |  |  |  |  |
| 84 | 19 | AF | arr[GRCh37] 16p13.11(15126890-16289532)×3 | 10 | 1.16 | I+II | Paternal | 46,XY | Abnormal NIPT results | No | Eutocia | Male | 2018/6/21 | 38 | 3000 | 51 | 1 months: L=53.4cm(<Mean); W=4.5kg(=Mean) 2 months: L=59.5cm(>Mean); W=5.7kg(>Mean); HC=38.4cm(<Mean)  12 months: L=73cm(<-1sd); W=8.8kg(<Mean); HC=44.5cm(<-1sd)  24 months: L=82cm(<-1sd); W=10.9kg(<Mean); HC=46cm(<-1sd) 36 months: L=90cm(<-1sd); W=12.3kg(<-1sd); HC=46.6cm(=-2sd) 62 months: L=103cm(<-1sd); W=14.8kg(<-1sd) 70 months: L=105cm(<-2sd); W=16.8kg(<-1sd) | Full-term delivery and AGA, Well survivor |
| 85 | 21 | VS | arr[GRCh37] 16p13.11(15052746-16308351)×3 | 10 | 1.26 | I+II | Refused | 46,XX | Absent or hypoplastic nasal bone | Yes | Eutocia | Female | 2018/10/22 | 40 | 3040 | 51 | 1 months: L=55cm(>Mean); W=4.45kg(>Mean); HC=37cm(>Mean) 3 months: L=60cm(>Mean); W=6.7kg(>+1sd); HC=41cm(>+1sd) 12 months: L=72.5cm(<Mean); W=8.5kg(<Mean); HC=45.6cm(>Mean) 24 months: L=84cm(<Mean); W=12.7kg(>Mean); HC=47.5cm(>Mean) 31 months: L=89cm(<Mean); W=13.1kg(>Mean); HC=49cm(>Mean) 53 months: L=104cm(<Mean); W=18.7kg(>Mean) 66 months: L=112cm(>Mean); W=20.8kg(>Mean) | W=3040g, L=51cm, HC=31.5cm, CC=32cm, Full-term delivery and AGA, Well survivor |
| 86 | 26 | AF | arr[GRCh37] 16p13.11(15239631-18156351)×3 | 9 | 2.92 | II+III | denovo | 45,XY,der(13,15)(q10,q10) | High-risk screening for trisomy 21 | No | Eutocia | Male | 2018/8/16 | 37 | 3400 | 51 | 1 months: L=54cm(<Mean); W=4.6kg(>Mean); HC=36cm(<-1sd) 3 months: L=65cm(>+1sd); W=6.5kg(>Mean); HC=41cm(>Mean) 11 months: L=76cm(>Mean); W=9.1kg(<Mean); HC=47cm(=+1sd) 19 months: L=82cm(<Mean); W=12.3kg(>Mean); HC=48cm(>Mean) 36 months: L=93cm(<Mean); W=14.8kg(>Mean); HC=48cm(=-1sd) 60 months: L=108cm(<Mean); W=16kg(=-1sd); HC=51cm(>Mean) 69 months: L=115cm(>Mean); W=22kg(>Mean) | Full-term delivery and AGA, Well survivor |
| 87 | 26 | UCB | arr[GRCh37] 16p13.12p13.11(14758345-16289532)×3 | 14 | 1.53 | I+II | Maternal | 46,XY | FGR | Yes | Cesarean | Male | 2018/11/1 | 37 | 2050 | 44 | 1 months: L=47cm(<-3sd); W=3.25kg(<-2sd); HC=37cm(<Mean) 3 months: L=53cm(<-3sd); W=4kg(=-3sd); HC=39cm(<-1sd)  12 months: L=75cm(<Mean); W=10kg(>Mean); HC=45cm(<Mean) 24 months: L=86cm(<Mean); W=12kg(<Mean); HC=49cm(>Mean) 37 months: L=96cm(<Mean); W=14.3kg(<Mean); HC=50.3cm(>Mean) 47 months: L=101cm(<Mean); W=17kg(>Mean); HC=50.5cm(>Mean) 52 months: L=104.5cm(<Mean); W=19.2kg(>Mean) 66 months: L=114cm(>Mean); W=22kg(>Mean) | Full-term delivery and SGA, Low birth weight infants (1500-2499g), Well survivor |
| 88 | 31 | UCB | arr[GRCh37] 16p13.11(15129970-16308351)×3 | 10 | 1.18 | I+II | Refused | 46,XY | Single umbilical artery | Yes | Eutocia | Male | 2018/6/27 | 37 | 2400 | 48 | 1 months: L=53cm(<Mean); W=3.9kg(=-1sd); HC=36cm(<-1sd) 3 months: L=59cm(<-1sd); W=6.5kg(>Mean); HC=39.5cm(<Mean) 12 months: L=71cm(=-2sd); W=8.3kg(<-1sd); HC=44cm(<-1sd) 24 months: L=82.2cm(<-1sd); W=10.9kg(<Mean); HC=47.1cm(<Mean) 36 months: L=92.5cm(<Mean); W=13kg(<Mean); HC=48.4cm(<Mean) 60 months: L=107.2cm(<Mean); W=21kg(>Mean); HC=50.5cm(<Mean) 69 months: L=113cm(<Mean); W=25kg(>+1sd) | Full-term delivery and SGA, Low birth weight infants (1500-2499g), Well survivor |
| 89 | 26 | UCB | arr[GRCh37] 16p13.11(15052746-16279283)×3 | 10 | 1.23 | I+II | Refused | 46,XN | Bilateral dilated renal pelvis (Left, 0.51cm; Right, 0.55 cm), high-risk screening for open neural tube defects (ONTD) | Yes | TP |  |  |  |  |  |  |  |
| 90 | 28 | AF | arr[GRCh37] 16p13.11(15127986-16279283)×3 | 10 | 1.15 | I+II | Refused | 46,XN | High-risk screening for trisomy 21 | No | TP |  |  |  |  |  |  |  |
| 91 | 41 | AF | arr[GRCh37] 16p13.11(15126890-16308351)×3 | 10 | 1.18 | I+II | Refused | 46,XN | Advanced maternal age | No | TP |  |  |  |  |  |  |  |
| 92 | 25 | VS | arr[GRCh37] 16p13.11p12.3(15499057-18156351)×3 | 9 | 2.66 | II+III | Paternal | 46,XY | Parent carried β-thalassemia gene | No | Eutocia | Male | 2019/6/21 | 40 | 2560 | 49 | 1 months: L=58cm(>+1sd); W=5kg(>Mean); HC=37.5cm(>Mean) 3 months: L=63.4cm(<Mean); W=6.6kg(>Mean); HC=40.8cm(>Mean) 12 months: L=75cm(<Mean); W=8.31kg(<-1sd); HC=44.5cm(<-1sd)  24 months: L=86cm(<Mean); W=10kg(<-1sd); HC=46.5cm(<-1sd) 30 months: L=92cm(>Mean); W=12.8kg(<Mean); HC=46cm(<-2sd) 36 months: L=96.2cm(>Mean); W=13.1kg(<Mean) 55 months: L=100.5cm(<Mean); W=14kg(<-1sd) 58 months: L=107cm(<Mean); W=15.2kg(<-1sd) | W=2560g, L=49cm, HC=32cm, CC=30cm, Full-term delivery and SGA, Well survivor |
| 93 | 35 | AF | arr[GRCh37] 16p13.11(15126890-16308351)×3 | 10 | 1.18 | I+II | Refused | 46,XN | Absent or hypoplastic nasal bone, advanced maternal age, DCDA, IVF | Yes | TP |  |  |  |  |  |  |  |
| 94 | 36 | AF | arr[GRCh37] 16p13.11(15494746-16308351)×3 | 7 | 0.81 | II | Paternal | 46,XX | Advanced maternal age | No | Cesarean | Female | 2019/6/11 | 40 | 4180 | 52 | 1 months: L=55.5cm(>Mean); W=4.8kg(=+1sd); HC=36.8cm(>Mean) 3 months: L=61.5cm(>Mean); W=6.35kg(>Mean); HC=41.5cm(>+1sd) 17 months: L=82cm(>Mean); W=10kg(=Mean); HC=46cm(<Mean) 24 months: L=85cm(<Mean); W=12kg(>Mean); HC=48cm(>Mean) 37 months: L=100cm(>+1sd); W=15.8kg(>Mean); HC=49cm(>Mean) 58 months: L=114.5cm(>+1sd); W=21kg(>+1sd) | Full-term delivery and LGA, Well survivor |
| 95 | 28 | AF | arr[GRCh37] 16p13.11(15052746-16308351)×3 | 10 | 1.26 | I+II | Refused | 46,XN | Right polycystic kidney | Yes | TP |  |  |  |  |  |  |  |
| 96 | 24 | AF | arr[GRCh37] 16p13.11(15127986-16308351)×3 | 10 | 1.18 | I+II | Paternal | 46,XY | Medication history in early pregnancy | No | Eutocia | Male | 2019/8/18 | 40 | 3030 | 50 | 1 months: L=56.1cm(>Mean); W=4.7kg(>Mean); HC=37.2cm(<Mean) 40 months: L=94cm(<-1sd); W=14kg(<Mean); HC=48cm(<-1sd) 49 months: L=104.2cm(>Mean); W=17.1kg(>Mean); HC=48.1cm(<-1sd) 57 months: L=110.2cm(>Mean); W=17.3kg(<Mean) | Full-term delivery and AGA, Well survivor |
| 97 | 26 | AF | arr[GRCh37] 16p13.11(15239631-16289532)×3 | 7 | 1.05 | II | Refused | 46,XX | High-risk screening for trisomy 13 | No | Cesarean | Female | 2018/8/3 | 39 | 3220 | 51 | 1 months: L=54cm(>Mean); W=4kg(<Mean); HC=36.5cm(=Mean) 3 months: L=60cm(>+1sd); W=6kg(>+1sd); HC=40.5cm(>+1sd) 12 months: L=74cm(=Mean); W=9.5kg(>Mean); HC=47cm(>+1sd) 24 months: L=89cm(>Mean); W=12.5kg(>Mean); HC=48.5cm(>Mean) 35 months: L=96cm(>Mean); W=14.2kg(>Mean); HC=50cm(>+1sd) 55 months: L=106.2cm(<Mean); W=19.6kg(>Mean) | Full-term delivery and AGA, Well survivor |
| 98 | 31 | AF | arr[GRCh37] 16p13.11(15052746-16308351)×3 | 10 | 1.26 | I+II | Refused | 46,XN | Absent or hypoplastic nasal bone | Yes | TP |  |  |  |  |  |  |  |
| 99 | 27 | VS | arr[GRCh37] 16p13.11(15052746-16308351)×3 | 10 | 1.26 | I+II | Paternal | 46,XY | Parent carried α-thalassemia gene | No | Cesarean | Male | 2020/5/15 | 39 | 3700 | 52 | 3 months: L=60.1cm(<Mean); W=4.8kg(<-2sd); HC=39cm(<-1sd) 12 months: L=73.1cm(<-1sd); W=7.25kg(<-2sd); HC=44.8cm(=-1sd)  24 months: L=84cm(<-1sd); W=10kg(<-1sd); HC=47cm(<Mean) 36 months: L=92cm(<-1sd); W=11.5kg(<-1sd); HC=48cm(=-1sd) 48 months: L=98.4cm(<-1sd); W=14.2kg(<-1sd); HC=49cm(<Mean) | Full-term delivery and AGA, Well survivor |
| 100 | 32 | VS | arr[GRCh37] 16p13.11(15127986-16308351)×3 | 10 | 1.18 | I+II | Maternal | 46,XY | Parent carried α-thalassemia gene | No | Eutocia | Male | 2020/1/23 | 38 | 2870 | 50 | 1 months: L=50.5cm(<-2sd); W=2.7kg(<-3sd); HC=35.5cm(<-1sd) 3 months: L=60cm(<Mean); W=5.65kg(<-1sd); HC=40.5cm(=Mean) 12 months: L=76cm(>Mean); W=9.6kg(=Mean); HC=46.6cm(>Mean)  24 months: L=88.5cm(>Mean); W=12.4kg(>Mean); HC=49cm(>Mean) 36 months: L=94cm(<Mean); W=14.2kg(<Mean); HC=50cm(>Mean) 51 months: L=102cm(<Mean); W=15kg(<Mean) | W=2870g, L=50cm, HC=33cm, CC=33cm, Full-term delivery and AGA, A subconjunctival hemorrhage in the right eye, GDD and umbilical hernia at 1 month, Torticollis at 5 months, Skin rash in all over the body (Dermatitis) at 8 months, LDD at 1 and 2 years |
| 101 | 34 | AF | arr[GRCh37] 16p13.11(15052746-16308351)×3 | 10 | 1.26 | I+II | Maternal | 46,XY | Left echogenic intracardiac focus (0.2×0.2cm), high-risk screening for trisomy 21 | Yes | Cesarean | Male | 2020/1/5 | 39 | 3200 | 50 | 1 months: L=55cm(>Mean); W=4.4kg(<Mean); HC=37cm(<Mean) 3 months: L=62cm(>Mean); W=6.25kg(<Mean); HC=39cm(<-1sd) 12 months: L=72.5cm(<-1sd); W=8.4kg(<-1sd); HC=44cm(<-1sd) 24 months: L=84.3cm(<-1sd); W=10.5kg(<-1sd); HC=45cm(<-1sd) 38 months: L=95.5cm(<Mean); W=13kg(<-1sd); HC=45.8cm(<-1sd) 42 months: L=97cm(<Mean); W=13.6kg(=-1sd) 51 months: L=101.5cm(<Mean); W=14.4kg(<-1sd) | Full-term delivery and AGA, Well survivor |
| 102 | 28 | VS | arr[GRCh37] 16p13.11(15052746-16308351)×3 | 10 | 1.26 | I+II | Maternal | 46,XX | Adverse pregnancy history, medication history in early pregnancy | No | Cesarean | Female | 2020/3/13 | 40 | 3005 | 52 | 1 months: L=52cm(<Mean); W=4.3kg(>Mean) 3 months: L=60cm(>Mean); W=5.8kg(=Mean); HC=38cm(<-1sd)  12 months: L=75.5cm(>Mean); W=9kg(>Mean); HC=43.5cm(=-1sd) 24 months: L=85.5cm(<Mean); W=12kg(>Mean); HC=45cm(<-1sd) 34 months: L=93cm(<Mean); W=12kg(<Mean); HC=46cm(<-1sd) 38 months: L=96cm(<Mean); W=13.3kg(<Mean) 49 months: L=102cm(<Mean); W=15kg(<Mean) | Full-term delivery and AGA, Well survivor |
| 103 | 35 | AF | arr[GRCh37] 16p13.11(15239631-16308351)×3 | 7 | 1.07 | II | Paternal | 46,XY | Adverse pregnancy history, advanced maternal age | No | Cesarean | Male | 2020/1/1 | 33 | 2230 | 46 | 1 months: L=52cm(<-1sd); W=3.4kg(=-2sd); HC=35cm(<-1sd) 5 months: L=66.5cm(>Mean); W=7kg(<Mean); HC=43cm(>Mean)  12 months: L=75cm(<Mean); W=10kg(>Mean); HC=47cm(>Mean)  24 months: L=87cm(<Mean); W=12.1kg(<Mean); HC=48.3cm(=Mean) 36 months: L=95cm(<Mean); W=13.3kg(<Mean); HC=49cm(<Mean) 39 months: L=98cm(=Mean); W=14kg(<Mean) 51 months: L=105cm(=Mean); W=15.5kg(<Mean) | W=2230g, L=46cm, HC=32cm, CC=30cm, Preterm delivery and AGA, Low birth weight infants (1500-2499g), Neonatal pneumonia, Hyperbilirubinemia, Feeding intolerance, Left subependymal hemorrhage (0.9×0.7cm), Patent foramen ovale (2mm), Right hydrocele (4.6×1.3cm), Bacterial conjunctivitis, Well survivor |
| 104 | 37 | VS | arr[GRCh37] 16p13.11(15052746-16308351)×3 | 10 | 1.26 | I+II | Paternal | 46,XX | Parent carried α-thalassemia gene, advanced maternal age | No | Cesarean | Female | 2020/4/4 | 38 | 3400 | 50 | 3 months: L=59cm(<Mean); W=6.2kg(>Mean); HC=49cm(>Mean)  12 months: L=72.3cm(<Mean); W=9kg(>Mean); HC=46cm(>Mean) 24 months: L=85cm(<Mean); W=11.5kg(=Mean); HC=47cm(<Mean) 36 months: L=95cm(<Mean); W=15kg(>Mean); HC=48.5cm(=Mean) 39 months: L=94cm(<Mean); W=14kg(<Mean) 49 months: L=98cm(<-1sd); W=15.1kg(<Mean) | Full-term delivery and AGA, Well survivor |
| 105 | 43 | AF | arr[GRCh37] 16p13.11(15569892-16308351)×3 | 6 | 0.74 | II | Paternal | 46,XY | Mild right hydronephrosis, advanced maternal age | Yes | Cesarean | Male | 2020/4/26 | 38 | 2980 | 50 | 1 months: L=54cm(<Mean); W=4kg(<Mean); HC=38.5cm(>+1sd)  4 months: L=65cm(>Mean); W=7.8kg(=+1sd); HC=43.5cm(>+1sd)  13 months: L=79cm(>Mean); W=10kg(>Mean); HC=47cm(>Mean) 24 months: L=89cm(>Mean); W=14kg(>+1sd); HC=49cm(>Mean) 36 months: L=99cm(>Mean); W=17kg(>+1sd); HC=52cm(>+1sd) 38 months: L=102cm(>+1sd); W=19.6kg(>+2sd) 47 months: L=108cm(>+1sd); W=22.5kg(>+2sd) | W=2980g, L=50cm, HC=35cm, CC=33cm, Full-term delivery and AGA, A red skin pigmentation about 3×2cm on the forehead of the newborn, Torticollis at 4 months |
| 106 | 33 | AF | arr[GRCh37] 16p13.11(15127986-16308351)×3 | 10 | 1.18 | I+II | Paternal | 46,XY | High-risk screening for trisomy 18 | No | Cesarean | Male | 2020/6/7 | 39 | 3200 | 50 | 1 months: L=56cm(>Mean); W=4.2kg(<Mean); HC=38cm(>Mean) 3 months: L=63.3cm(>Mean); W=5.8kg(<Mean); HC=41.3cm(>Mean)  12 months: L=77cm(>Mean); W=8.4kg(<-1sd); HC=46cm(<Mean) 24 months: L=91cm(>+1sd); W=10.7kg(<-1sd); HC=48cm(<Mean) 36 months: L=94cm(<Mean); W=15kg(>Mean); HC=49cm(<Mean) 48 months: L=105.3cm(>Mean); W=15kg(<Mean) | Full-term delivery and AGA, Well survivor |
| 107 | 21 | AF | arr[GRCh37] 16p13.11p12.3(15239631-18156351)×3 | 9 | 2.92 | II+III | Maternal | 46,XY | Widened inferior vena cava | Yes | Cesarean | Male | 2020/4/30 | 39 | 3650 | 52 | 1 months: L=58cm(>+1sd); W=5kg(>Mean); HC=38.5cm(>+1sd) 2 months: L=63cm(>+2sd); W=6.7kg(>+1sd); HC=41cm(>+1sd) 12 months: L=77.5cm(>Mean); W=9.5kg(<Mean); HC=45.8cm(<Mean) 36 months: L=97cm(>Mean); W=12.6kg(<-1sd); HC=48cm(=-1sd) 48 months: L=105cm(>Mean); W=17.8kg(>Mean) | Full-term delivery and AGA, Well survivor |
| 108 | 33 | UCB | arr[GRCh37] 16p13.11(15140211-16308351)×3 | 10 | 1.17 | I+II | Refused | 46,XY | High-risk screening for trisomy 21, adverse pregnancy history | No | Eutocia | Male | 2020/6/3 | 39 | 3575 | 51 | 1 months: L=58cm(>+1sd); W=5kg(>Mean); HC=37cm(<Mean) 3 months: L=64cm(>+1sd); W=6.3kg(<Mean); HC=38cm(<-2sd) 12 months: L=74.8cm(<Mean); W=8.3kg(<-1sd); HC=44.3cm(<-1sd) 36 months: L=93.2cm(<Mean); W=12.7kg(=-1sd); HC=47cm(<-1sd) 48 months: L=109.5cm(>+1sd); W=14.4kg(=-1sd) | Full-term delivery and AGA, Hyperspasmia and epilepsy at 6 months |
| 109 | 33 | VS | arr[GRCh37] 16p13.11p12.3(15578856-18986309)×3 | 12 | 3.41 | II+III | Refused | 46,XN | Parent carried β-thalassemia gene | No | TP |  |  |  |  |  |  | Thalassemia result, β17/βIVS-I-1, Fetus with severe β-thalassemia |
| 110 | 30 | AF | arr[GRCh37] 16p13.11(15127986-16279283)×3 | 10 | 1.15 | I+II | Refused | 46,XN | Parent carried β-thalassemia gene, medication history in early pregnancy | No | TP |  |  |  |  |  |  | Thalassemia result, αCSα/αα, Fetus with static α-thalassemia |
| 111 | 33 | AF | arr[GRCh37] 16p13.11(15127986-16289532)×3 | 10 | 1.16 | I+II | Maternal | 46,XX | High-risk screening for trisomy 21 | No | Cesarean | Female | 2020/10/8 | 38 | 3100 | 50 | 1 months: L=57cm(>+1sd); W=4.4kg(>Mean); HC=36cm(<Mean) 3 months: L=62cm(>+1sd); W=6.3kg(>Mean); HC=40cm(>Mean) 12 months: L=76cm(>Mean); W=8.3kg(<Mean); HC=45.7cm(>Mean) 30 months: L=92.5cm(>Mean); W=12.3kg(<Mean); HC=48cm(>Mean) 34 months: L=96cm(>Mean); W=12kg(<Mean)  42 months: L=100cm(>Mean); W=14.5kg(<Mean) | Full-term delivery and AGA, Well survivor |
| 112 | 32 | AF | arr[GRCh37] 16p13.11(15126890-16289532)×3 | 10 | 1.16 | I+II | Maternal | 46,XN | Hyperechogenic bowel (2.12×1.55×2.06cm), high-risk screening for trisomy 21 | Yes | TP |  |  |  |  |  |  | Mother with pityrosporum folliculitis and dermatitis (Case 238) |
| 113 | 24 | VS | arr[GRCh37] 16p13.11(15127986-16279283)×3 | 10 | 1.15 | I+II | Paternal | 46,XX | Parent carried β-thalassemia gene | No | Eutocia | Female | 2020/12/12 | 40 | 3300 | 50 | 1 months: L=55cm(>Mean); W=4.4kg(>Mean); HC=34.5cm(<-1sd) 3 months: L=62cm(>+1sd); W=6.2kg(>Mean); HC=38.5cm(<Mean) 24 months: L=88cm(>Mean); W=13kg(=+1sd); HC=44.5cm(<-1sd) 36 months: L=102cm(>+1sd); W=18kg(>+1sd); HC=47.5cm(<Mean) | Full-term delivery and AGA, Well survivor |
| 114 | 25 | VS | arr[GRCh37] 16p13.11(15129970-16308351)×3 | 10 | 1.18 | I+II | Maternal | 46,XX | Parent carried α-thalassemia gene | No | Eutocia | Female | 2020/12/6 | 39 | 3000 | 50 | 1 months: L=53cm(<Mean); W=4.2kg(=Mean); HC=37cm(>Mean) 3 months: L=60cm(>Mean); W=6kg(>Mean); HC=40cm(<Mean) 12 months: L=74.5cm(>Mean); W=8.57kg(<Mean); HC=45.5cm(>Mean) 39 months: L=94cm(<Mean); W=13.5kg(<Mean) 41 months: L=101cm(>Mean); W=14kg(<Mean) | Full-term delivery and AGA, Well survivor |
| 115 | 29 | UCB | arr[GRCh37] 16p13.11(15239631-16289532)×3 | 7 | 1.05 | II | Paternal | 46,XN | Arachnoid cyst (2.1×2.0×2.1cm) | Yes | TP |  |  |  |  |  |  |  |
| 116 | 21 | AF | arr[GRCh37] 16p13.11p12.3(15493046-18093086)×3 | 9 | 2.6 | II+III | Refused | 46,XY | Adverse contact history in early pregnancy, medication history in early pregnancy | No | Eutocia | Male | 2020/11/1 | 37 | 2970 | 50 | 1 months: L=53cm(<Mean); W=3.95kg(<Mean); HC=36cm(<-1sd) 3 months: L=58.7cm(<-1sd); W=6.2kg(<Mean); HC=39.3cm(=-1sd) 11 months: L=75.3cm(>Mean); W=10kg(>Mean); HC=46.7cm(>Mean) 24 months: L=89.3cm(>Mean); W=12.8kg(>Mean); HC=49.5cm(>Mean) 36 months: L=97.5cm(>Mean); W=15kg(>Mean); HC=50.5cm(>Mean) | Full-term delivery and AGA, Recurrent bronchitis and pneumonia, Well survivor |
| 117 | 41 | VS | arr[GRCh37] 16p13.11p12.3(15239631-18156351)×3 | 9 | 2.92 | II+III | denovo | 46,XN | Widened inner diameter of rectum (0.74cm), adverse pregnancy history (Trisomy 21), advanced maternal age | Yes | TP |  |  |  |  |  |  |  |
| 118 | 34 | AF | arr[GRCh37] 16p13.11(15129970-16308351)×3 | 10 | 1.18 | I+II | Refused | 46,XY | Parent carried α-thalassemia gene | No | Eutocia | Male | 2020/12/31 | 38 | 3150 | 53 | 1 months: L=55cm(>Mean); W=4.15kg(<Mean); HC=36.5cm(<Mean) 3 months: L=61.5cm(>Mean); W=6.4kg(=Mean); HC=40.5cm(=Mean) 12 months: L=73.7cm(<Mean); W=10kg(>Mean); HC=46cm(<Mean) 24 months: L=82.2cm(<-1sd); W=11.2kg(<Mean); HC=47.6cm(<Mean) 36 months: L=90cm(<-1sd); W=13kg(<Mean); HC=49cm(<Mean) | Full-term delivery and AGA, Well survivor |
| 119 | 26 | AF | arr[GRCh37] 16p13.11(15126890-16633361)×3 | 11 | 1.51 | I+II | Maternal | 46,XY | Adverse pregnancy history (albinism) | No | Eutocia | Male | 2020/12/26 | 37 | 2850 | 49 | 1 months: L=54cm(<Mean); W=4.2kg(<Mean); HC=37cm(<Mean) 3 months: L=58cm(<-1sd); W=5.8kg(<Mean); HC=39cm(<-1sd) 12 months: L=72cm(<-1sd); W=8.5kg(<-1sd); HC=44cm(<-1sd) 24 months: L=82cm(<-1sd); W=10.8kg(=-1sd); HC=46cm(<-1sd) 36 months: L=90cm(<-1sd); W=13.8kg(<Mean); HC=47.2cm(<-1sd) 41 months: L=97cm(<Mean); W=13.5kg(<Mean) | Full-term delivery and AGA, Well survivor |
| 120 | 36 | AF | arr[GRCh37] 16p13.11p12.3(15499057-18156351)×3 | 9 | 2.66 | II+III | Refused | 46,XY | Advanced maternal age, MCDA | No | Cesarean | Male | 2020/12/3 | 31 | 2050 | 46 | 1 months: L=56.4cm(=-1sd); W=4.1kg(<-2sd); HC=35.5cm(<-3sd) 3 months: L=58.5cm(<-1sd); W=5.9kg(<Mean); HC=37.2cm(<-2sd) 12 months: L=78.8cm(>+1sd); W=10.1kg(>Mean); HC=45.8cm(<Mean) 26 months: L=93cm(>+1sd); W=14.5kg(>+1sd); HC=49cm(>Mean) 29 months: L=94cm(>Mean); W=14kg(>Mean); HC=49cm(>Mean) 40 months: L=103cm(>+1sd); W=16kg(>Mean) | Preterm delivery and AGA, Low birth weight infants (1500-2499g), Well survivor |
| 121 | 36 | AF | arr[GRCh37] 16p13.11p12.3(15499057-18156351)×3 | 9 | 2.66 | II+III | Refused | 46,XY | Advanced maternal age, MCDA | No | Cesarean | Male | 2020/12/3 | 31 | 1650 | 40 | 1 months: L=53.6cm(<-2sd); W=3.5kg(<-3sd); HC=35cm(<-3sd) 3 months: L=57cm(<-2sd); W=5.4kg(<-1sd); HC=37.4cm(<-2sd) 12 months: L=77cm(>Mean); W=9.8kg(>Mean); HC=44.8cm(=-1sd) 26 months: L=90cm(>Mean); W=13kg(>Mean); HC=47cm(<-1sd) 29 months: L=94cm(>Mean); W=14kg(>Mean); HC=48.6cm(<Mean) 40 months: L=104cm(>+1sd); W=16kg(>Mean) | Preterm delivery and AGA, Low birth weight infants (1500-2499g), Well survivor |
| 122 | 28 | UCB | arr[GRCh37] 16p13.11(15127986-16308351)×3 | 10 | 1.18 | I+II | Paternal | 46,XX | Thickened placenta (4.2cm) | Yes | Eutocia | Female | 2020/11/4 | 35 | 2450 | 46 | 1 months: L=51cm(<-1sd); W=3.5kg(<-1sd) 3 months: L=53cm(<-3sd); W=4kg(=-3sd); HC=37cm(<-2sd) 12 months: L=70cm(<-1sd); W=8kg(<Mean); HC=44cm(<Mean) 24 months: L=84cm(<Mean); W=11kg(<Mean); HC=47cm(<Mean) 36 months: L=92cm(<Mean); W=12kg(<-1sd); HC=48cm(<Mean) 40 months: L=96cm(<Mean); W=12.4kg(<-1sd) | Preterm delivery and AGA, Low birth weight infants (1500-2499g), Well survivor |
| 123 | 29 | AF | arr[GRCh37] 16p13.11(15052746-16633361)×3 | 11 | 1.58 | I+II | denovo | 46,XY | Left choroid plexus cysts (5mm) | Yes | Eutocia | Male | 2021/2/19 | 36 | 2630 | 47 | 1 months: L=56cm(>Mean); W=4.3kg(<Mean); HC=37cm(<Mean) 3 months: L=62cm(>Mean); W=6.2kg(<Mean); HC=41.7cm(=+1sd) 12 months: L=76cm(>Mean); W=10.5kg(>Mean); HC=49cm(>+2sd) 24 months: L=90cm(>Mean); W=13.5kg(>Mean); HC=51cm(=+2sd) 36 months: L=99.5cm(>Mean); W=15.7kg(>Mean); HC=51.5cm(>+1sd) 37 months: L=101.1cm(>+1sd); W=15.3kg(<Mean) 39 months: L=101.8cm(>Mean); W=15.3kg(>Mean) | Preterm delivery and AGA, NEC, Neonatal hyperbilirubinemia, Abnormal coagulation function, Patent foramen ovale (2mm), Immature retina, Sinus rhythm, Occasional premature beat, Skin rash and itching all over the body (Dermatitis), Bronchitis, Myopia (0.6 score) at 3 years |
| 124 | 21 | AF | arr[GRCh37] 16p13.11p12.3(15239631_18156351)×3 | 9 | 2.92 | II+III | Refused | 46,XY | Left renal agenesis or dysplasia | Yes | Eutocia | Male | 2021/1/29 | 37 | 3800 | 51 | 1 months: L=54cm(<Mean); W=4.6kg(>Mean); HC=37.7cm(>Mean) 3 months: L=61.5cm(>Mean); W=6.8kg(>Mean); HC=41cm(>Mean) 13 months: L=74cm(<-1sd); W=10.1kg(>Mean); HC=46.8cm(>Mean) 24 months: L=86.4cm(<Mean); W=12.3kg(>Mean); HC=49cm(>Mean) 37 months: L=96cm(<Mean); W=13.9kg(<Mean); HC=49.6cm(>Mean) 40 months: L=96cm(<Mean); W=15kg(=Mean) | Full-term delivery and LGA, Well survivor |
| 125 | 32 | AF | arr[GRCh37] 16p13.11(15052746_16279283)×3 | 10 | 1.23 | I+II | Maternal | 46,XY | Right choroid plexus cysts (0.3×0.24cm), aberrant right subclavicular artery, high-risk screening for trisomy 21, adverse pregnancy history (cleft lip and palate) | Yes | Cesarean | Male | 2021/3/20 | 38 | 3400 | 50 | 1 months: L=55cm(>Mean); W=4.6kg(>Mean); HC=37cm(<Mean) 3 months: L=61cm(<Mean); W=6.7kg(>Mean); HC=40.5cm(=Mean) 15 months: L=77.1cm(<Mean); W=9.3kg(<Mean); HC=45cm(<-1sd) 24 months: L=83cm(<-1sd); W=10.7kg(<-1sd); HC=47.5cm(<Mean) 31 months: L=88.8cm(<-1sd); W=11.9kg(<-1sd) 38 months: L=94.7cm(<Mean); W=12.7kg(<-1sd); HC=48.3cm(<Mean) 40 months: L=94.9cm(<Mean); W=12.8kg(<-1sd) | Full-term delivery and AGA, Well survivor |
| 126 | 32 | AF | arr[GRCh37] 16p13.11(15156180_16633361)×3 | 11 | 1.48 | I+II | Refused | 46,XY | Adverse pregnancy history (G2P2, a girl and boy with hypotonia, cannot hold up head, die young) | No | Eutocia | Male | 2021/4/8 | 38 | 3100 | 51 | 1 months: L=51cm(<-1sd); W=4kg(<Mean); HC=36.8cm(<Mean) 3 months: L=58cm(<-1sd); W=5.7kg(=-1sd); HC=39.5cm(<Mean) 12 months: L=73.5cm(<Mean); W=8.3kg(<-1sd); HC=44cm(<-1sd) 24 months: L=86cm(<Mean); W=11kg(<Mean); HC=48cm(<Mean) 30 months: L=88cm(<-1sd); W=11.3kg(<-1sd) 36 months: L=91.5cm(<-1sd); W=11.3kg(=-2sd); HC=48.5cm(<Mean) | Full-term delivery and AGA, Well survivor |
| 127 | 28 | AF | arr[GRCh37] 16p13.11(15127986_16308351)×3 | 10 | 1.18 | I+II | Refused | 46,XX,t(1,7)(q11,q11) | Maternal chromosomal balanced translocation (46,XX,t(1,7)(q11,q11)), PGD | No | Eutocia | Female | 2021/4/26 | 40 | 3350 | 50 | 1 months: L=53cm(<Mean); W=4.2kg(=Mean); HC=36cm(<Mean) 3 months: L=61cm(>Mean); W=5.9kg(>Mean); HC=40cm(>Mean) 13 months: L=75cm(<Mean); W=8.5kg(<Mean); HC=45.5cm(>Mean) 24 months: L=86cm(<Mean); W=10.6kg(<Mean); HC=47.9cm(>Mean) 36 months: L=93cm(<Mean); W=12.5kg(<Mean); HC=48.5cm(=Mean) | W=3350g, L=50cm, HC=33cm, CC=34cm, Full-term delivery and AGA, Well survivor |
| 128 | 28 | AF | arr[GRCh37] 16p13.11(15129970_16308351)×3 | 10 | 1.18 | I+II | Paternal | 46,XY | Abnormal NIPT results | No | Eutocia | Male | 2021/4/19 | 38 | 2710 | 49 | 1 months: L=56.8cm(>+1sd); W=5.2kg(>+1sd); HC=38.5cm(>+1sd) 6 months: L=67.5cm(<Mean); W=8.6kg(>Mean); HC=43cm(<Mean) 9 months: L=73.2cm(>Mean); W=9.5kg(>Mean) 36 years: L=100cm(>+1sd); W=14.7kg(>Mean); HC=49.5cm(=Mean) | W=2710g, L=49cm, HC=33cm, CC=32cm, Full-term delivery and AGA, Neonatal pathological jaundice, Feeding intolerance for 9 months, MDD, Concealed penis, Astigmatism at 3 years |
| 129 | 18 | AF | arr[GRCh37] 16p13.11(15127986_16279283)×3 | 10 | 1.15 | I+II | Maternal | 46,XX | Left echogenic intracardiac focus (0.2×0.2cm), small cavum septum pellucidum (0.07cm) | Yes | Eutocia | Female | 2021/4/17 | 40 | 3550 | 51 | 1 months: L=55cm(>Mean); W=4.3kg(>Mean); HC=37.7cm(=+1sd) 3 months: L=61.3cm(>Mean); W=5.4kg(<Mean); HC=40cm(>Mean) 12 months: L=73.4cm(<Mean); W=7.85kg(<-1sd); HC=45cm(>Mean) 24 months: L=84.5cm(<Mean); W=9.9kg(<-1sd); HC=47.5cm(>Mean) 30 months: L=88cm(<Mean); W=11.2kg(=-1sd) 36 months: L=91cm(<-1sd); W=12kg(<-1sd); HC=49.2cm(>Mean) | W=3350g, L=51cm, HC=34cm, CC=33cm, Full-term delivery and AGA, A caput succedaneum about 6×7cm, Well survivor |
| 130 | 37 | AF | arr[GRCh37] 16p13.12p13.11(14750607_16308351)×3 | 14 | 1.56 | I+II | Paternal | 46,XX | Adverse pregnancy history (recurrent miscarriage twice), advanced maternal age | No | Eutocia | Female | 2021/6/9 | 40 | 3390 | 50 | 1 months: L=53.6cm(<Mean); W=4kg(<Mean); HC=36cm(<Mean) 3 months: L=65.5cm(>+2sd); W=6.4kg(>Mean); HC=39cm(<Mean) 12 months: L=78.2cm(>+1sd); W=9.3kg(>Mean); HC=44cm(<Mean) 24 months: L=93cm(>+2sd); W=12.9kg(>Mean); HC=46.5cm(<Mean) 30 months: L=95cm(>+1sd); W=14.9kg(>+1sd) 36 months: L=102.7cm(=+2sd); W=17.5kg(>+1sd); HC=48.3cm(<Mean) | Full-term delivery and AGA, Well survivor |
| 131 | 28 | AF | arr[GRCh37] 16p13.11(15127986_16308351)×3 | 10 | 1.18 | I+II | Maternal | 46,XX | High-risk screening for trisomy 21 | No | Eutocia | Female | 2021/7/24 | 38 | 3150 | 50 | 1 months: L=54.5cm(>Mean); W=4.3kg(>Mean); HC=35cm(<-1sd) 3 months: L=60cm(>Mean); W=5.75kg(<Mean); HC=37.8cm(<-1sd) 6 months: L=65cm(<Mean); W=8kg(>Mean); HC=41.5cm(<Mean) 18 months: L=78cm(<Mean); W=9.5kg(<Mean); HC=45cm(<Mean) 24 months: L=83cm(<-1sd); W=9.3kg(<-1sd); HC=46cm(<Mean) 30 months: L=87cm(<-1sd); W=11.8kg(<Mean); HC=46cm(<-1sd) 36 months: L=91.5cm(<Mean); W=13kg(<Mean); HC=47cm(<-1sd) | Full-term delivery and AGA, Well survivor |
| 132 | 29 | AF | arr[GRCh37] 16p13.11(15126890_16277685)×3 | 10 | 1.15 | I+II | Refused | 46,XX | Abnormal NIPT results | No | Eutocia | Female | 2021/9/14 | 40 | 3400 | 54 | 1 months: L=55.4cm(>Mean); W=4.17kg(<Mean); HC=37.4cm(>Mean) 3 months: L=59.4cm(<Mean); W=5.6kg(<Mean); HC=39cm(<Mean) 6 months: L=65.9cm(>Mean); W=7.3kg(=Mean); HC=42.4cm(>Mean) 12 months: L=72.5cm(<Mean); W=8.3kg(<Mean); HC=45.5cm(>Mean) 18 months: L=80cm(<Mean); W=10.5kg(>Mean); HC=47cm(>Mean) | Full-term delivery and AGA, Well survivor |
| 133 | 28 | AF | arr[GRCh37] 16p13.11(15147788_16308351)×3 | 10 | 1.16 | I+II | Refused | 46,XY | Short femur and humerus length (<-2sd), adverse pregnancy history (16p13.11dup) | Yes | Eutocia | Male | 2021/9/4 | 39 | 3000 | 50 | 1 months: L=52.7cm(<-1sd); W=3.9kg(=-1sd); HC=35cm(<-1sd) 3 months: L=59.3cm(<-1sd); W=6.1kg(<Mean); HC=40cm(<Mean) 6 months: L=63.2cm(<-2sd); W=7kg(<-1sd); HC=42cm(<-1sd) 12 months: L=69.5cm(<-2sd); W=8kg(<-1sd); HC=44.8cm(=-1sd) 25 months: L=82.3cm(<-1sd); W=10.4kg(<-1sd); HC=47.3cm(<Mean) 30 months: L=84.5cm(<-2sd); W=11.3kg(<-1sd); HC=47.5cm(=-1sd) 31 months: L=87.8cm(<-1sd); W=11kg(<-1sd) | Full-term delivery and AGA, Well survivor |
| 134 | 31 | UCB | arr[GRCh37] 16p13.11(15052746_16308351)×3 | 10 | 1.26 | I+II | Refused | 46,XY | Fetal left mandible subcutaneous fluid mass (3.0×1.4×2.4cm) | Yes | Cesarean | Male | 2021/8/3 | 39 | 2980 | 49 | 1 months: L=55cm(>Mean); W=4.6kg(>Mean); HC=37.3cm(=Mean) 3 months: L=62cm(>Mean); W=6.2kg(<Mean); HC=41cm(>Mean) 12 months: L=75cm(<Mean); W=8.3kg(<-1sd); HC=46cm(<Mean) 24 months: L=87cm(<Mean); W=10.6kg(<-1sd); HC=48.3cm(=Mean) 31 months: L=92cm(<Mean); W=12kg(=-1sd) 36 months: L=96cm(<Mean); W=13kg(<Mean); HC=50cm(>Mean) | W=2980g, L=49cm, HC=34cm, CC=32cm, Full-term delivery and AGA, A 3×2cm left mandibular lymphangioma, Skin rash and drying all over the body, Atopic dermatitis |
| 135 | 33 | VS | arr[GRCh37] 16p13.11(15493046_16308351)×3 | 7 | 0.82 | II | Refused | 46,XY | Parent carried α-thalassemia gene | No | Eutocia | Male | 2021/11/11 | 39 | 3350 | 51 | 1 months: L=54.4cm(<Mean); W=4.3kg(<Mean); HC=36.7cm(<Mean) 3 months: L=60.3cm(<Mean); W=6kg(<Mean); HC=39.8cm(<Mean) 12 months: L=74.6cm(<Mean); W=8.8kg(<Mean); HC=45.8cm(<Mean) 24 months: L=86cm(<Mean); W=10.7kg(<-1sd); HC=48cm(<Mean) 29 months: L=89cm(<Mean); W=11.5kg(<-1sd); HC=48.3cm(<Mean) | Full-term delivery and AGA, Well survivor |
| 136 | 29 | AF | arr[GRCh37] 16p13.11(15052746_16308351)×3 | 10 | 1.26 | I+II | Refused | 46,XX | Aberrant right subclavicular artery | Yes | Eutocia | Female | 2021/10/23 | 38 | 3440 | 50 | 1 months: L=53.9cm(>Mean); W=4.7kg(>Mean); HC=37cm(>Mean) 4 months: L=60.5cm(<Mean); W=6.7kg(>Mean); HC=40.5cm(<Mean) 12 months: L=73cm(<Mean); W=8.3kg(<Mean); HC=45cm(>Mean) 24 months: L=85cm(<Mean); W=10.4kg(<Mean); HC=46cm(<Mean) | W=3440g, L=50.0cm, HC=34.0cm, CC=35.0cm, Full-term delivery and AGA, Bilateral eye subconjunctival hemorrhage, CHD, Second foramen atrial septal defect (type II), Venous sinus defect, Coronary sinus defect, Patent foramen ovale (2mm), Patent ductus arteriosus (1mm) |
| 137 | 34 | VS | arr[GRCh37] 16p13.11(15126890_16212104)×3 | 10 | 1.09 | I+II | Paternal | 46,XX | Parent carried α-thalassemia gene | No | Cesarean | Female | 2021/12/27 | 38 | 3230 | 51 | 1 months: L=56cm(>+1sd); W=4.7kg(>Mean); HC=37cm(>Mean) 3 months: L=60.5cm(>Mean); W=5.2kg(=-1sd); HC=39cm(<Mean) 13 months: L=73cm(<Mean); W=8.5kg(<Mean); HC=44cm(<Mean) 25 months: L=83cm(<-1sd); W=11kg(<Mean); HC=46cm(<Mean) 30 months: L=88.6cm(<Mean); W=12kg(<Mean); HC=46.5cm(=-1sd) | Full-term delivery and AGA, Well survivor |
| 138 | 32 | AF | arr[GRCh37] 16p13.11(15126890_16308351)×3 | 10 | 1.18 | I+II | Maternal | 46,XX,inv(9)(p12q13) | Elevated HCGb, paternal chromosomal pericentric inversion (46,XY,inv(9)(p12q21)), adverse pregnancy history | No | Eutocia | Female | 2021/11/24 | 39 | 3500 | 52 | 1 months: L=54.9cm(>Mean); W=4.9kg(>+1sd); HC=37.4cm(>Mean) 3 months: L=61.4cm(>+2sd); W=6.31kg(>+1sd); HC=39cm(>Mean) 13 months: L=79.2cm(>+1sd); W=11.15kg(>+1sd); HC=39cm(>Mean) 24 months: L=88.2cm(>Mean); W=13kg(=+1sd); HC=47cm(<Mean) 30 months: L=92.3cm(>Mean); W=14.2kg(>Mean); HC=48.2cm(>Mean) | Full-term delivery and AGA, Well survivor |
| 139 | 27 | AF | arr[GRCh37] 16p13.11(15127986_16308351)×3 | 10 | 1.18 | I+II | Paternal | 46,XX | Thickened nuchal translucency (2.6mm) | Yes | Cesarean | Female | 2021/12/13 | 39 | 2880 | 48 | 1 months: L=52cm(<Mean); W=3.65kg(<Mean); HC=35.8cm(<Mean) 3 months: L=58cm(<Mean); W=4.85kg(<-1sd); HC=39cm(<Mean) 11 months: L=70cm(<-1sd); W=7kg(<-1sd); HC=43.3cm(<Mean) 17 months: L=74.3cm(<-1sd); W=7.9kg(=-2sd); HC=45cm(<Mean) 21 months: L=78cm(<-1sd); W=8.6kg(=-2sd) 32 months: L=87.5cm(<-1sd); W=10.41kg(<-1sd) | W=2880g, L=48.0cm, HC=33.0cm, CC=34.0cm, Full-term delivery and AGA, Pharyngitis, Bronchitis, pneumonia, Innutrition, Well survivor |
| 140 | 27 | AF | arr[GRCh37] 16p13.11(15493046_16289532)×3 | 7 | 0.8 | II | Refused | 46,XX | Oligohydramnios, shallow and flat parietal occipital sulcus, small cavum septum pellucidum (0.17cm), high-risk screening for trisomy 21 | Yes | Cesarean | Female | 2022/1/1 | 38 | 3000 | 48 | 1 months: L=51cm(<-1sd); W=4.28kg(>Mean); HC=36.5cm(=Mean) 3 months: L=57.5cm(<-1sd); W=5.35kg(<Mean); HC=39.2cm(<Mean) 12 months: L=72.3cm(<Mean); W=8.6kg(<Mean); HC=44cm(<Mean) 24 months: L=80cm(=-2sd); W=10.1kg(<-1sd); HC=46.5cm(<Mean) 30 months: L=85.5cm(<-1sd); W=10.4kg(<-1sd); HC=46.5cm(=-1sd) | Full-term delivery and AGA, Well survivor |
| 141 | 37 | AF | arr[GRCh37] 16p13.11(15156180_16289532)×3 | 10 | 1.13 | I+II | Refused | 46,XY | Advanced maternal age, HBV carriers | No | Cesarean | Male | 2022/1/7 | 39 | 3260 | 50 | 1 months: L=56cm(>Mean); W=4.7kg(>Mean); HC=36.7cm(<Mean) 3 months: L=61.5cm(>Mean); W=6.8kg(>Mean); HC=39cm(<-1sd) 12 months: L=75cm(<Mean); W=8.2kg(<-1sd); HC=45cm(<Mean) 24 months: L=87.3cm(<Mean); W=10.7kg(<-1sd); HC=46.6cm(<-1sd) 30 months: L=90cm(<Mean); W=11.8kg(=-1sd); HC=48cm(<Mean) | W=3260g, L=50.0cm, HC=34.0cm, CC=33.0cm, Full-term delivery and AGA, Acute pharyngitis, Kawasaki disease, Glucose-6-phosphatase deficiency |
| 142 | 27 | AF | arr[GRCh37] 16p13.11(15127986_16308351)×3 | 10 | 1.18 | I+II | Maternal | 46,XY | Adverse pregnancy history (hydrocephalus) | No | Cesarean | Male | 2022/3/3 | 40 | 3560 | 53 | 1 months: L=57.1cm(>+1sd); W=4.8kg(>Mean); HC=37.5cm(>Mean) 3 months: L=65cm(>+1sd); W=7.4kg(>+1sd); HC=42cm(>+1sd) 12 months: L=79cm(>+1sd); W=10.8kg(=+1sd); HC=46cm(<Mean) 24 months: L=88cm(>Mean); W=12.8kg(>Mean); ; HC=49cm(>Mean) 30 months: L=92cm(>Mean); W=14kg(>Mean); ; HC=50cm(>Mean) | Full-term delivery and AGA, Well survivor |
| 143 | 28 | AF | arr[GRCh37] 16p13.11p12.3(15493046_18143302)×3 | 9 | 2.65 | II+III | Paternal | 46,XN | Parent carried β-thalassemia gene | No | TP |  |  |  |  |  |  | Thalassemia result, βIVS-Ⅱ-654/β17, Fetus with severe β-thalassemia |
| 144 | 33 | VS | arr[GRCh37] 16p13.11(15127986_16308351)×3 | 10 | 1.18 | I+II | Refused | 46,XY | Thickened pulmonary valve, increased anterior blood flow velocity of pulmonary valve (113cm/s), mild pulmonary regurgitation, mild tricuspid regurgitation, high-risk screening for trisomy 21 | Yes | Eutocia | Male | 2022/7/1 | 38 | 3170 | 50 | 1 months: L=54cm(<Mean); W=4.2kg(<Mean); HC=36cm(<-1sd) 3 months: L=60.5cm(<Mean); W=6.5kg(>Mean); HC=39.5cm(<Mean) 6 months: L=68cm(>Mean); W=8kg(>Mean); HC=42.5cm(<Mean) 12 months: L=75.5cm(<Mean); W=9.6kg(=Mean); HC=45cm(<Mean) 18 months: L=80cm(<Mean); W=10.7kg(<Mean); HC=46cm(=-1sd) | W=3170g, L=50.0cm, HC=35.0cm, CC=33.0cm, Full-term delivery and AGA, Neonatal pathological jaundice, Left eye nasolacrimal duct obstruction, Increased anterior blood flow velocity of pulmonary valve, Mild pulmonary regurgitation, Mild tricuspid regurgitation, Respiratory infection |
| 145 | 29 | AF | arr[GRCh37] 16p13.11(15052746_16289532)×3 | 10 | 1.24 | I+II | Maternal | 46,XN | Parent carried α-thalassemia gene | No | TP |  |  |  |  |  |  | Thalassemia result, αα/αα |
| 146 | 29 | AF | arr[GRCh37] 16p13.11(15126890_16308351)×3 | 10 | 1.18 | I+II | Refused | 46,XX | High-risk screening for trisomy 21 | No | Eutocia | Female | 2022/8/9 | 39 | 3160 | 53 | 1 months: L=57cm(>+1sd); W=5kg(>+1sd); HC=37.5cm(>Mean) 3 months: L=64cm(=+2sd); W=7.5kg(=+2sd); HC=41cm(>+1sd) 6 months: L=71cm(>+2sd); W=9.3kg(=+2sd); HC=45cm(>+2sd) 12 months: L=78cm(>+1sd); W=10.7kg(>+1sd); HC=45cm(>Mean) 24 months: L=91cm(>+1sd); W=13.2kg(>+1sd); HC=48cm(>Mean) | Full-term delivery and AGA, Well survivor |
| 147 | 34 | AF | arr[GRCh37] 16p13.11(15052746_16289532)×3 | 10 | 1.24 | I+II | Refused | 46,XY | Bilateral ventriculomegaly (1.2cm) | Yes | Eutocia | Male | 2022/8/15 | 38 | 3800 | 49 | 1 months: L=54cm(<Mean); W=4.4kg(<Mean); HC=38.5cm(>+1sd) 3 months: L=63.5cm(=+1sd); W=7kg(>Mean); HC=42cm(>+1sd) 6 months: L=65cm(<-1sd); W=8.2kg(>Mean); HC=44cm(>Mean) 13 months: L=76cm(<Mean); W=9.7kg(<Mean); HC=46cm(<Mean) 24 months: L=82.5cm(<-1sd); W=11.3kg(<Mean); HC=49cm(>Mean) | Full-term delivery and LGA, Well survivor |
| 148 | 25 | UCB | arr[GRCh37] 16p13.11(15052746_16308351)×3 | 10 | 1.26 | I+II | Refused | 46,XY | Left hydronephrosis, right dilated renal pelvis | Yes | Eutocia | Male | 2022/8/27 | 39 | 3050 | 50 | 1 months: L=54cm(<Mean); W=4.3kg(<Mean); HC=37.5cm(>Mean) 3 months: L=61cm(<Mean); W=5.5kg(<-1sd); HC=41.5cm(>Mean) 6 months: L=67cm(<Mean); W=7.1kg(=-1sd); HC=43cm(<Mean) 12 months: L=75cm(<Mean); W=8.5kg(<-1sd); HC=46cm(<Mean) 18 months: L=78cm(<-1sd); W=9.7kg(<-1sd); HC=46.5cm(<Mean) 24 months: L=87cm(<Mean); W=10kg(<-1sd); HC=47cm(<Mean) | Full-term delivery and AGA, Well survivor |
| 149 | 33 | AF | arr[GRCh37] 16p13.12p13.11(14758345_16308351)×3 | 14 | 1.55 | I+II | Paternal | 46,XY | Aberrant right subclavicular artery, adverse pregnancy history (cleft lip and palate) | Yes | Cesarean | Male | 2022/11/7 | 35 | 2250 | 47 | 2 months: L=54cm(<-2sd); W=5.3kg(<Mean)  6 months: L=65cm(<-1sd); W=7.2kg(<Mean); HC=43cm(<Mean)  8 months: L=67cm(<-1sd); W=7.9kg(<Mean); HC=44cm(<Mean)  18 months: L=79.8cm(<Mean); W=10.2kg(<Mean); HC=46cm(=-1sd) | W=2250g, L=47.0cm, HC=30.0cm, CC=31.0cm, Preterm delivery and AGA, Low birth weight infants (1500-2499g), Jaundice, Neonatal hyperbilirubinemia, NEC, Patent foramen ovale, Well survivor, Father with dermatitis (Case 261) |
| 150 | 40 | AF | arr[GRCh37] 16p13.11p12.3(15493046_18116792)×3 | 9 | 2.62 | II+III | Refused | 46,XN | Advanced maternal age | No | TP |  |  |  |  |  |  |  |
| 151 | 26 | AF | arr[GRCh37] 16p13.11(15627274_16308964)×3 | 6 | 0.63 | II | Refused | 46,XY | High-risk screening for trisomy 21 | No | Cesarean | Male | 2022/12/22 | 34 | 1700 | 44 | 1 months: L=46.6cm(<-1sd); W=2.6kg(<-1sd); HC=33.3cm(<Mean) 3 months: L=54.7cm(<-3sd); W=4.3kg(<-3sd); HC=36.5cm(<-3sd) 6 months: L=63.5cm(<-1sd); W=6kg(<-2sd); HC=41cm(<-1sd) 12 months: L=72cm(<-1sd); W=7.2kg(<-2sd); HC=44cm(<-1sd) 18 months: L=76.4cm(<-2sd); W=8.2kg(<-2sd); HC=46.1cm(<Mean) | Preterm delivery and SGA, Low birth weight infants (1500-2499g), Jaundice, NEC, Neonatal metabolic acidosis, Hypoalbuminemia, Hypophosphatemia, Patent foramen ovale (2mm), Left subependymal hemorrhage (0.6×0.4cm), Conjunctivitis, Unvascularized retina |
| 152 | 32 | UCB | arr[GRCh37] 16p13.11(15052746_16308351)×3 | 10 | 1.26 | I+II | Refused | 46,XY | Left renal cyst (0.8×0.7cm) | Yes | Cesarean | Male | 2022/12/6 | 36 | 2350 | 47 | 1 months: L=51.4cm(<-1sd); W=3.4kg(=-2sd); HC=35.2cm(<-1sd) 3 months: L=58.4cm(<-1sd); W=5kg(=-2sd); HC=39cm(<-1sd) 6 months: L=67.6cm(=Mean); W=6.5kg(<-1sd); HC=42cm(<-1sd) 12 months: L=75cm(<Mean); W=9.2kg(<Mean); HC=44cm(<-1sd) 14 months: L=77.8cm(<Mean); W=9.7kg(<Mean) 18 months: L=81cm(<Mean); W=10.9kg(=Mean); HC=46cm(=-1sd) | Preterm delivery and AGA, Low birth weight infants (1500-2499g), Well survivor |
| 153 | 37 | AF | arr[GRCh37] 16p13.11(15129970_16308351)×3 | 10 | 1.18 | I+II | Refused | 46,XY | Bilateral choroid plexus cysts (Left, 0.6×0.3cm; Right, 0.5×0.2cm), advanced maternal age | Yes | Cesarean | Male | 2023/2/5 | 38 | 3000 | 50 | 1 months: L=52cm(<-1sd); W=3.8kg(<-1sd) 3 months: L=60cm(<Mean); W=6.4kg(=Mean); HC=41cm(>Mean) 6 months: L=68cm(=+1sd); W=7.9kg(>Mean); HC=44cm(>+1sd) 8 months: L=76cm(>+2sd); W=8.5kg(<Mean); HC=45.7cm(>Mean) 12 months: L=79cm(>+1sd); W=9.6kg(=Mean); HC=48cm(>+1sd) 18 months: L=85cm(=+1sd); W=11.4kg(>Mean); HC=49cm(>+1sd) | Full-term delivery and AGA, Well survivor |
| 154 | 27 | AF | arr[GRCh37] 16p13.11(15052746_16308351)×3 | 10 | 1.26 | I+II | Refused | 46,XY | Oligohydramnios, high-risk screening for trisomy 21, adverse contact history in early pregnancy | Yes | Eutocia | Male | 2023/2/17 | 39 | 2900 | 50 | 1 months: L=55cm(>Mean); W=4kg(<Mean); HC=37cm(<Mean) 3 months: L=63cm(>Mean); W=5.6kg(<-1sd); HC=40.5cm(=Mean) 8 months: L=71cm(>Mean); W=7.85kg(<Mean); HC=43cm(<-1sd) 12 months: L=75.5cm(<Mean); W=8.7kg(<Mean); HC=44cm(<-1sd) 18 months: L=82cm(<Mean); W=9.8kg(=-1sd); HC=45cm(<-1sd) | W=2900g, L=50.0cm, HC=34.0cm, CC=32.0cm, Full-term delivery and AGA, Glucose-6-phosphatase deficiency, Well survivor |
| 155 | 35 | AF | arr[GRCh37] 16p13.11p12.3(15493046_18986309)×3 | 13 | 3.49 | II+III | Maternal | 46,XN | Abnormal echo in the liver (0.8×0.8×0.8cm), hyperechogenic bowel, advanced maternal age | Yes | TP |  |  |  |  |  |  |  |
| 156 | 29 | UCB | arr[GRCh37] 16p13.11(15705605_16308351)×3 | 6 | 0.6 | II | Refused | 46,XY | Subependymal cysts, absent or hypoplastic nasal bone, widened cavum septum pellucidum, high-risk screening for trisomy 21 | Yes | Cesarean | Male | 2023/2/14 | 38 | 3300 | 50 | 1 months: L=53cm(<Mean); W=4.6kg(>Mean); HC=37.5cm(>Mean) 3 months: L=62.5cm(>Mean); W=6.3kg(<Mean); HC=39cm(<-1sd) 6 months: L=72cm(>+2sd); W=8.9kg(>+1sd); HC=42cm(<-1sd) 8 months: L=72cm(>Mean); W=9.1kg(>Mean); HC=42.3cm(<-1sd) 12 months: L=77cm(>Mean); W=10kg(>Mean); HC=46cm(<Mean) 18 months: L=84cm(>Mean); W=12kg(>Mean) | Full-term delivery and AGA, Well survivor |
| 157 | 37 | VS | arr[GRCh37] 16p13.11(15052746_16633361)×3 | 11 | 1.58 | I+II | Refused | 46,XY | Adverse pregnancy history (twin pregnancy-one of the twins has stopped), advanced maternal age | No | Eutocia | Male | 2023/5/29 | 40 | 2790 | 49 | 1 months: L=53.5cm(<Mean); W=3.9kg(=-1sd); HC=36.5cm(<Mean) 3 months: L=61.2cm(>+1sd); W=5.4kg(<Mean); HC=38cm(=-1sd) 6 months: L=67cm(<Mean); W=7kg(<-1sd); HC=41cm(<-1sd) 8 months: L=70cm(<Mean); W=7.7kg(=-1sd); HC=42cm(=-2sd) 12 months: L=73.5cm(<Mean); W=8.2kg(<-1sd); HC=43cm(<-2sd) | W=2790g, L=49.0cm, HC=32.0cm, CC=32.0cm, Full-term delivery and SGA, MDD at 6 months, Conjunctivitis, Lacrimal duct stenosis, Left cryptorchidism |
| 158 | 35 | AF | arr[GRCh37] 16p13.11p12.2(15499057_22670586)×3 | 45 | 7.17 | II+III | Refused | 46,XN,dup(16)(p13.1p12.2) | Adverse pregnancy history, advanced maternal age | No | TP |  |  |  |  |  |  |  |
| 159 | 31 | AF | arr[GRCh37] 16p13.11(15126890_16289532)×3 | 10 | 1.16 | I+II | Maternal | 46,XX | Parent carried α-thalassemia gene | No | Cesarean | Female | 2023/6/2 | 39 | 3150 | 52 | 1 months: L=54cm(<Mean); W=4kg(<Mean);  3 months: L=59cm(<Mean); W=5.5kg(<Mean); HC=39cm(<Mean) 6 months: L=64cm(<Mean); W=6.5kg(=-1sd); HC=41cm(<Mean) 8 months: L=67.5cm(<Mean); W=7.5kg(<Mean); HC=42.3cm(<Mean) 12 months: L=71cm(<-1sd); W=8.1kg(<Mean); HC=42.5cm(<-1sd) | Full-term delivery and AGA, Well survivor |
| 160 | 35 | AF | arr[GRCh37] 16p13.11(15126890_16308351)×3 | 10 | 1.18 | I+II | Maternal | 46,XX | FGR, bilateral choroid plexus cysts (Left, 0.7×0.4cm; Right, 0.4×0.3cm), hyperechogenic bowel (3.2×2.0×2.2cm), advanced maternal age, maternal 16p13.11 duplication | Yes | Eutocia | Female | 2023/7/19 | 39 | 2440 | 46 | 1 months: L=51cm(<-1sd); W=3.8kg(<Mean) 3 months: L=56cm(<-1sd); W=5kg(<-1sd); HC=39.5cm(=Mean) 6 months: L=61cm(<-2sd); W=5.9kg(<-1sd); HC=43cm(>Mean) 8 months: L=63cm(<-2sd); W=6kg(<-2sd); HC=43.5cm(>Mean) 12 months: L=68.5cm(<-2sd); W=7kg(=-2sd); HC=45.7cm(>Mean) | W=2440g, L=46.0cm, HC=31.0cm, CC=30.0cm, Full-term delivery and SGA, Low birth weight infants (1500-2499g), Neonatal hypoglycemia and pneumonia, Patent ductus arteriosus (1.5mm), Patent foramen ovale (3mm), Aortic stenosis (3.1mm), Mother with pityrosporum folliculitis and dermatitis (Case 238) |
| 161 | 27 | AF | arr[GRCh37] 16p13.11(15052746_16308351)×3 | 10 | 1.26 | I+II | Maternal | 46,XY | Abnormal NIPT results | No | Eutocia | Male | 2023/7/21 | 40 | 3750 | 53 | 1 months: L=56.5cm(>Mean); W=5kg(>Mean) 3 months: L=65cm(>+1sd); W=7.1kg(>Mean); HC=41.2cm(>Mean) 6 months: L=70cm(>+1sd); W=8kg(>Mean); HC=44cm(>Mean) 8 months: L=72cm(>Mean); W=9kg(>Mean); HC=44.5cm(=Mean) 11 months: L=76.5cm(>Mean); W=9.8kg(>Mean); HC=46.2cm(>Mean) | Full-term delivery and AGA, Well survivor |
| 162 | 38 | AF | arr[GRCh37] 16p13.11(15131523_16666154)×3 | 11 | 1.53 | I+II | denovo | 46,XY | Advanced maternal age | No | Cesarean | Male | 2023/7/17 | 38 | 3200 | 49 | 1 months: L=55cm(>Mean); W=4.3kg(<Mean);  3 months: L=61cm(<Mean); W=5.6kg(<-1sd); HC=39.1cm(<-1sd) 6 months: L=67.7cm(>Mean); W=7.1kg(=-1sd); HC=41.8cm(<-1sd) 8 months: L=68.5cm(<Mean); W=7.5kg(<-1sd); HC=42cm(=-2sd) 12 months: L=74cm(<Mean); W=7.6kg(<-2sd); HC=43cm(<-2sd) | Full-term delivery and AGA, Well survivor |
| 163 | 28 | VS | arr[GRCh37] 16p13.11p12.3(15239631_18156351)×3 | 9 | 2.92 | II+III | Maternal | 46,XY | Thickened nuchal translucency (2.7mm) | Yes | Eutocia | Male | 2023/9/16 | 39 | 3150 | 50 | 1 months: L=56cm(>Mean); W=4.5kg(=Mean) 3 months: L=65cm(>+1sd); W=7.4kg(>+1sd); HC=41cm(>Mean) 6 months: L=69.3cm(>Mean); W=8.75kg(>Mean); HC=45cm(>+1sd) 8 months: L=73cm(>+1sd); W=8.5kg(<Mean); HC=46cm(>+1sd) 12 months: L=79cm(>+1sd); W=9.75kg(>Mean); HC=47cm(>Mean) | Full-term delivery and AGA, Well survivor |
| 164 | 28 | VS | arr[GRCh37] 16p13.11(15052746_16308351)×3 | 10 | 1.26 | I+II | Maternal | 46,XY | Maternal 16p13.11 duplication, parent carried α-thalassemia gene | No | Eutocia | Male | 2023/10/1 | 40 | 3000 | 50 | 1 months: L=56cm(>Mean); W=5.6kg(>+1sd) 3 months: L=64cm(>+1sd); W=6.9kg(>Mean); HC=42cm(>+1sd) 6 months: L=70cm(>+1sd); W=7.5kg(<Mean); HC=44cm(>Mean) 8 months: L=72cm(>Mean); W=8kg(<Mean); HC=45cm(>Mean) 12 months: L=78cm(>Mean); W=8.8kg(<Mean); HC=45.5cm(<Mean) | Full-term delivery and AGA, Well survivor |
| 165 | 36 | UCB | arr[GRCh37] 16p13.11(15239631_16308351)×3 | 7 | 1.07 | II | Refused | 46,XX | Small head circumference (=-2.24sd), advanced maternal age | Yes | Cesarean | Female | 2023/6/19 | 39 | 3350 | 50 | 1 months: L=54cm(>Mean); W=4.7kg(>Mean) 3 months: L=60cm(>Mean); W=5.9kg(>Mean); HC=38cm(<-1sd) 6 months: L=65cm(<Mean); W=7kg(<Mean); HC=39cm(<-2sd) 12 months: L=71cm(<-1sd); W=7.5kg(<-1sd); HC=43cm(<-1sd) | Full-term delivery and AGA, Well survivor |
| 166 | 25 | AF | arr[GRCh37] 16p13.12p12.3(14761435_17211891)×3 | 15 | 2.45 | I+II | Maternal | / | Complex cardiac malformation, complete endocardial cushion defect, right ventricular double outlet | Yes | TP |  |  |  |  |  |  |  |
| 167 | 30 | VS | arr[GRCh37] 16p13.11 (15126890_16308351)×3 | 10 | 1.18 | I+II | Refused | 46,XN | Parent carried β-thalassemia gene | No | TP |  |  |  |  |  |  | Thalassemia result, β17/β-28, Fetus with severe β-thalassemia |
| 168 | 43 | AF | arr[GRCh37] 16p13.11(15129970_16308351)×3 | 10 | 1.18 | I+II | Refused | 46,XY | Advanced maternal age | No | Cesarean | Male | 2023/10/26 | 39 | 3200 | 51 | 1 months: L=53.3cm(<Mean); W=4.2kg(<Mean) 3 months: L=61cm(<Mean); W=6.2kg(<Mean); HC=39.6cm(<Mean) 6 months: L=67cm(<Mean); W=7.5kg(<Mean); HC=42.5cm(<Mean) 8 months: L=69.6cm(<Mean); W=8.1kg(<Mean); HC=43.5cm(<Mean)  12 months: L=77cm(>Mean); W=9.7kg(>Mean); HC=44.3cm(<-1sd) | Full-term delivery and AGA, Well survivor |
| 169 | 36 | VS | arr[GRCh37] 16p13.11p12.3(15626960_18156351)×3 | 8 | 2.53 | II+III | Refused | 46,XN | Thickened nuchal translucency (3.1mm), syndactyly (Apert syndrome), adverse pregnancy history (G4P2, cleft lip and palate), advanced maternal age | Yes | TP |  |  |  |  |  |  |  |
| 170 | 36 | AF | arr[GRCh37] 16p13.11(15052746_16308351)×3 | 10 | 1.26 | I+II | denovo | 46,XN | Advanced maternal age | No | TP |  |  |  |  |  |  |  |
| 171 | 22 | VS | arr[GRCh37] 16p13.11p12.3(15239631_18143302)×3 | 9 | 2.9 | II+III | Refused | 46,XN | Thickened nuchal translucency (2.9mm), parent carried α-thalassemia gene | Yes | TP |  |  |  |  |  |  | Thalassemia result, Bart's syndrome, Fetus with severe α-thalassemia |
| 172 | 36 | AF | arr[GRCh37] 16p13.11 (15129970_16308351)×3 | 10 | 1.18 | I+II | Refused | 46,XY | Adverse pregnancy history, advanced maternal age | No | Eutocia | Male | 2023/11/25 | 37 | 3400 | 50 | 1 months: L=55.3cm(>Mean); W=4.9kg(>Mean) 3 months: L=63cm(>Mean); W=6.8kg(>Mean); HC=41cm(>Mean) 6 months: L=69cm(>Mean); W=8.2kg(>Mean); HC=45.7cm(>+1sd) 8 months: L=73.3cm(>+1sd); W=9.6kg(=+1sd); HC=47.3cm(>+2sd) | Full-term delivery and AGA, Well survivor |
| 173 | 39 | AF | arr[GRCh37] 16p13.11(15052746_16279283)×3 | 10 | 1.23 | I+II | Refused | 46,XY | Adverse pregnancy history, advanced maternal age | No | Cesarean | Male | 2023/12/6 | 38 | 3210 | 49 | 3 months: L=62cm(>Mean); W=7.1kg(<Mean); HC=41.7cm(=+1sd) 6 months: L=69.3cm(>Mean); W=8.7kg(>Mean); HC=44.6cm(=+1sd)  8 months: L=73cm(>+1sd); W=8.9kg(>Mean); HC=44.8cm(>Mean) 12 months: L=79cm(>+1sd); W=9.9kg(>Mean); HC=46cm(<Mean) | W=3210g, L=49.0cm, HC=34.0cm, CC=33.0cm, Full-term delivery and AGA, Well survivor |
| 174 | 36 | AF | arr[GRCh37] 16p13.11(15140211_16289532)×3 | 10 | 1.15 | I+II | Refused | 46,XY | Bilateral choroid plexus cysts (Left, 0.84×0.29cm; Right, 0.97×0.39cm), thickened nuchal translucency (3.0mm), HBV carriers, IVF, advanced maternal age | Yes | Cesarean | Male | 2024/1/15 | 38 | 2900 | 50 | 1 months: L=55cm(>Mean); W=4.6kg(>Mean) 3 months: L=62cm(>Mean); W=7.3kg(>+1sd); HC=41.2cm(>Mean) 6 months: L=68cm(>Mean); W=8.5kg(>Mean); HC=44.5cm(>Mean)  8 months: L=70.5cm(<Mean); W=8.5kg(<Mean); HC=44.5cm(=Mean) | W=2900g, L=50.0cm, HC=33.0cm, CC=32.0cm, Full-term delivery and AGA, Well survivor |
| 175 | 37 | AF | arr[GRCh37] 16p13.11(15126890_16308351)×3 | 10 | 1.18 | I+II | Paternal | 46,XY | Parent carried β-thalassemia gene, advanced maternal age | No | Eutocia | Male | 2024/3/15 | 39 | 2810 | 49 | 1 months: L=55cm(>Mean); W=4.6kg(>Mean) 3 months: L=62cm(>Mean); W=6.9kg(>Mean); HC=41cm(>Mean) 6 months: L=67.6cm(=Mean); W=7.6kg(<Mean); HC=42.9cm(<Mean)  8 months: L=70.2cm(<Mean); W=8.1kg(<Mean); HC=43.3cm(=-1sd) | Full-term delivery and SGA, Patchy erythema and papules on the thighs and upper limbs (dermatitis) |
| 176 | 43 | AF | arr[GRCh37] 16p13.11(15129970_16308351)×3 | 10 | 1.18 | I+II | Refused | 46,XX | Advanced maternal age | No | Cesarean | Female | 2024/3/29 | 40 | 2950 | 51 | 1 months: L=51cm(<-1sd); W=3.9kg(<Mean) 3 months: L=60.3cm(>Mean); W=5.1kg(<-1sd); HC=40.2cm(>Mean) 6 months: L=65cm(<Mean); W=6.5kg(=-1sd)  8 months: L=69cm(>Mean); W=7.1kg(<Mean); HC=43.4cm(=Mean) | Full-term delivery and AGA, Well survivor |
| 177 | 31 | AF | arr[GRCh37] 16p13.11p12.3(15493046_18986309)×3 | 13 | 3.49 | II+III | Refused | 46,XX | Abnormal NIPT result, high-risk screening for trisomy 21 | No | Eutocia | Female | 2024/3/26 | 38 | 3465 | 50 | 1 months: L=52cm(<-1sd); W=4.8kg(>Mean);  3 months: L=61cm(>Mean); W=7.6kg(>+2sd); HC=41cm(<+1sd) 6 months: L=66cm(>Mean); W=8.7kg(>+1sd); HC=43.5cm(=+1sd)  8 months: L=68cm(<Mean); W=8.8kg(>Mean); HC=44.1cm(>Mean) | Full-term delivery and AGA, Well survivor |
| 178 | 33 | AF | arr[GRCh37] 16p13.11(15126890_16768780)×3 | 11 | 1.64 | I+II | Refused | 46,XN | High-risk screening for trisomy 18 and 21 | No | TP |  |  |  |  |  |  |  |
| 179 | 20 | AF | arr[GRCh37] 16p13.11(15140211_16279283)×3 | 10 | 1.14 | I+II | Refused | 46,XY | Parent carried α-thalassemia gene | No | Eutocia | Male | 2024/7/14 | 37 | 2500 | 48 | 1 months: L=53.3cm(<Mean); W=3.8kg(<-1sd) 3 months: L=61.3cm(<Mean); W=6kg(<Mean); HC=40cm(<Mean) | Full-term delivery and AGA, Well survivor |
| 180 | 37 | AF | arr[GRCh37] 16p13.11(15494087_16287134)×3 | 7 | 0.79 | II | Paternal | 46,XY | Advanced maternal age | No | Eutocia | Male | 2024/5/13 | 38 | 2920 | 48 | 1 months: L=54.5cm(<Mean); W=4.8kg(>Mean)  3 months: L=60cm(<Mean); W=6kg(<Mean); HC=40.5cm(=Mean)  6 months: L=66.8cm(<Mean); W=7.6kg(<Mean); HC=44cm(>Mean) | W=2920g, L=48.0cm, HC=30.0cm, CC=30.0cm, Full-term delivery and AGA, A red skin pigmentation about 2×1cm on the right eyelid of the newborn |
| 181 | 34 | UCB | arr[GRCh37] 16p13.11(15052746_16768780)×3 | 11 | 1.72 | I+II | Refused | 46,XX | Hyperechogenic bowel, high-risk screening for open neural tube defects (ONTD) | Yes | Eutocia | Female | 2024/6/5 | 38 | 3030 | 50 | 1 months: L=53.5cm(<Mean); W=4.4kg(>Mean) 3 months: L=62cm(>+1sd); W=7.1kg(>+1sd); HC=40cm(>Mean) 6 months: L=67cm(>Mean); W=8.5kg(>+1sd); HC=42cm(<Mean) | Full-term delivery and AGA, Well survivor |
| 182 | 29 | AF | arr[GRCh37] 16p13.11(15147155_16319120)×3 | 10 | 1.17 | I+II | Maternal | 46,XX | Bilateral choroid plexus cysts, IVF | Yes | Cesarean | Female | 2024/7/18 | 39 | 2850 | 50 | 1 months: L=53cm(<Mean); W=3.7kg(<Mean) 3 months: L=60cm(>Mean); W=5.7kg(<Mean); HC=40cm(>Mean) | Full-term delivery and AGA, Well survivor |
| 183 | 36 | AF | arr[GRCh37] 16p13.11(15121567_16696042)×3 | 11 | 1.57 | I+II | Refused | 46,XY | Maternal chromosomal karyotype (45,X[3]/46，XX[51]), adverse pregnancy history (G3P0A2, 16p13.11 dup), advanced maternal age, IVF | No | Cesarean | Male | 2024/7/27 | 36 | 2530 | 47 | 1 months: L=53cm(<Mean); W=3.9kg(=-1sd) 3 months: L=59cm(>Mean); W=5.5kg(<Mean); HC=40.2cm(>Mean) | W=2530g, L=47.0cm, HC=33.0cm, CC=31.5cm, Preterm delivery and AGA, A depression can be seen at the midline of the soft palate, the local mucosa is relatively thin, Well survivor |
| 184 | 37 | VS | arr[GRCh37] 16p13.11(15127986_16308351)×3 | 10 | 1.18 | I+II | Refused | 46,XY | Parent carried β-thalassemia gene, advanced maternal age | No | Eutocia | Male | 2024/9/4 | 36 | 2870 | 49 | / | Preterm delivery and AGA, Thalassemia result, αWSα/αα, β17/βN, Fetus with mild thalassemia, Well survivor |
| 185 | 37 | AF | arr[GRCh37] 16p13.12p13.11(14771789_16308351)×3 | 13 | 1.54 | I+II | Refused | 46,XY | Abnormal NIPT results, advanced maternal age | No | Cesarean | Male | 2024/8/9 | 40 | 2740 | 49 | 1 months: L=56.5cm(>Mean); W=4.8kg(>Mean) | W=2740g, L=49.0cm, HC=32.0cm, CC=32.0cm, Full-term delivery and SGA, Well survivor |
| 186 | 23 | VS | arr[GRCh37] 16p13.11p12.3(15239631_18116792)×3 | 9 | 2.88 | II+III | Refused | 46,XX | Thickened nuchal translucency (2.9mm), parent carried α-thalassemia gene | Yes | Eutocia | Female | 2024/10/2 | 39 | 2500 | 48 | 1 months: L=53cm(<Mean); W=4kg(<Mean) 3 months: L=59cm(<Mean); W=5.5kg(<Mean); HC=40cm(>Mean) | Full-term delivery and SGA, Thalassemia result, αα/--, Fetus with mild α-thalassemia, Well survivor |
| 187 | 26 | AF | arr[GRCh37] 16p13.11(15052746_16308351)×3 | 10 | 1.26 | I+II | Refused | 46,XX | Adverse pregnancy history (duodenal obstruction) | No | Cesarean | Female | 2024/9/23 | 39 | 3200 | 51 | 1 months: L=53cm(<Mean); W=3.8kg(<Mean) 3 months: L=58.2cm(<Mean); W=5.3kg(<Mean); HC=38.5cm(<Mean) | Full-term delivery and AGA, Well survivor |
| 188 | 28 | VS | arr[GRCh37] 16p13.11(15126890_16633361)×3 | 11 | 1.51 | I+II | Paternal | 46,XY | Parent carried α-thalassemia gene | No | Eutocia | Male | 2024/10/28 | 38 | 3300 | 53 | 1 months: L=55.5cm(>Mean); W=4.3kg(<Mean) | Full-term delivery and AGA, Thalassemia result, αα/--, Fetus with mild α-thalassemia, Well survivor |
| 189 | 29 | AF | arr[GRCh37] 16p13.11(15493046_16672757)×3 | 8 | 1.18 | II | Refused | 46,XY | High-risk screening for trisomy 21 | No | Cesarean | Male | 2024/9/29 | 40 | 3500 | 52 | 1 months: L=57cm(>+1sd); W=5kg(>Mean) 3 months: L=65.5cm(=+2sd); W=8kg(=+2sd); HC=41cm(>Mean) | Full-term delivery and AGA, Well survivor |
| 190 | 24 | AF | arr[GRCh37] 16p13.11(15935444_16305868)×3 | 4 (NO NDE1) | 0.37 | II | denovo | 46,XY | Persistent right umbilical vein | Yes | Eutocia | Male | 2024/9/23 | 38 | 2860 | 48 | 1 months: L=52cm(<-1sd); W=3.5kg(<-1sd) 3 months: L=57.3cm(=-2sd); W=5.75kg(<Mean); HC=38.5cm(<-1sd) | Full-term delivery and AGA, Well survivor |
| 191 | 22 | AF | arr[GRCh37] 16p13.11(15126890_16236431)×3, 22q11.23(23692738_24992266)×3 | 9 | 1.11 | I+II | Paternal | 46,XX | High-risk screening for trisomy 21 | No | Eutocia | Female | 2017/11/27 | 40 | 2780 | 47 | 1 months: L=52.2cm(<Mean); W=4.6kg(>Mean) 3 months: L=60cm(>Mean); W=5.25kg(<Mean); HC=38cm(<-1sd)  12 months: L=72cm(<Mean); W=8kg(<Mean); HC=44cm(<Mean) 24 months: L=85cm(<Mean); W=10.5kg(<Mean); HC=46.2cm(<Mean) 36 months: L=93cm(<Mean); W=13kg(<Mean); HC=48cm(<Mean) 51 months: L=104cm(<Mean); W=15.5kg(<Mean) 75 months: L=119cm(>Mean); W=21.5kg(>Mean) | Full-term delivery and SGA, Fetus with maternal 22q11.23 duplication, Well survivor |
| 192 | 28 | AF | arr (18)×3, arr[GRCh37] 16p13.11(15129970_16308351)×3 | 10 | 1.18 | I+II | Refused | 47,XN,+18 | Abnormal NIPT result | No | TP |  |  |  |  |  |  | Fetus with trisomy 18 |
| 193 | 28 | AF | arr[GRCh37] 16p13.11(15239631_16289532)×3, 16q12.2q22.1(53909185_69042861)×3 | 7 | 1.05 | II | Maternal | 46,XN,dup(16)(q12.2q22.1) | Adverse pregnancy history | No | TP |  |  |  |  |  |  | Fetus with denovo 16q12.2q22.1 duplication |
| 194 | 28 | AF | arr (Y)×0~1, arr[GRCh37] 16p13.11(15127986_16308351)×3 | 10 | 1.18 | I+II | Refused | 45,X | High-risk screening for trisomy 18 | No | TP |  |  |  |  |  |  | Fetus with chromosome Y chimerism |
| 195 | 28 | UCB | arr[GRCh37] Xp22.31(6690232_7855994)×1, 16p13.11(15239631_16682080)×3 | 8 | 1.44 | II | denovo | 46,XN | Oligohydramnios | Yes | TP |  |  |  |  |  |  | Fetus with maternal Xp22.31 deletion |
| 196 | 27 | AF | arr[GRCh37] 8p23.3p23.1(176818_11455106)×1, 16p13.11(15129970_16308351)×3 | 10 | 1.18 | I+II | Refused | 46,XN,del(8)(p23.3p23.1) | Abnormal NIPT result, advanced maternal age | No | TP |  |  |  |  |  |  | Fetus with 8p23.3p23.1 deletion |
| 197 | 27 | AF | arr(X)×1, arr[GRCh37] 16p13.11(15156180_16308351)×3 | 9 | 1.15 | I+II | Refused | 45,X | Nuchal cystic hygroma, fetal hydrops, MCDA | Yes | TP |  |  |  |  |  |  | Fetus with XO |
| 198 | 27 | AF | arr[GRCh37] 16p13.11(15127986_16212104)×3 | 9 | 1.08 | I+II | Refused | 46,XN | Abnormal ultrasound in another one of twins, MCDA | No | TP |  |  |  |  |  |  |  |
| 199 | 30 | UCB | arr[GRCh37] Xp22.33(43118_2697868)×1, Xp22.33p22.31(2704609_7228499)×0, Xp22.3p22.12(7244205_20293730)×2, (Y)×1, 16p13.11(15052746_16308351)×3 | 10 | 1.26 | I+II | Refused | 46,Y,dup(X)(p22.3p22.12) | Cerebellar hypoplasia, absent or hypoplastic nasal bone, single umbilical artery | Yes | TP |  |  |  |  |  |  | Fetus with abnormal chromosome X |
| 200 | 38 | AF | arr(18)×3, arr[GRCh37] 16p13.11(15127986_16308351)×3 | 10 | 1.18 | I+II | Refused | 47,XN,+18 | Parent carried β-thalassemia gene, advanced maternal age | No | TP |  |  |  |  |  |  | Fetus with trisomy 18 |
| 201 | 33 | VS | arr(21)×3, arr[GRCh37] 16p13.11(15052746_16279283)×3 | 10 | 1.23 | I+II | Refused | 47,XN,+21 | Thickened nuchal translucency | Yes | TP |  |  |  |  |  |  | Fetus with trisomy 21 |
| VS, villus sampling; AF, amniotic fluid; UCB, umbilical cord blood; TP, termination of pregnancy; FGR, fetal growth restriction; CHD, congenital heart disease; NEC, neonatal necrotizing enterocolitis; SGA, small for gestational age; AGA, appropriate for gestational age; LGA, large for gestational age; W, weight; L, length; HC, head circumference; CC, chest circumference; Well survivor, the level of growth and development in line with peers; DD, global developmental delay; GDD, growth developmental delay; MDD, motor developmental delay; LDD, language developmental delay; DCDA, dichorionic diamniotic twins; MCDA, monochorionic diamniotic twins. Case 120 and 121 are twins. Case 197 and 198 are twins. Case 171 and 186 have the same mother; Case 114 and 164 have the same mother (Case 258); Case 89 and 136 have the same mother; Case 112 and 160 have the same mother (Case 255). These 18 fetuses with abnormal postnatal phenotypes in outcome follow-up are highlighted in blue. These 10 fetuses with additional genomic abnormalities are depicted in green. | | | | | | | | | | | | | | | | | | |
|  |  |  |  |  |  |  |  |  |  |  |  |  |  |  |  |  |  |  |
|  |  |  |  |  |  |  |  |  |  |  |  |  |  |  |  |  |  |  |
|  |  |  |  |  |  |  |  |  |  |  |  |  |  |  |  |  |  |  |

| Table S3. Ultrasound characteristics and outcome follow-up of 31 fetuses with 16p13.11 deletion. | | | | | | | | | | |
| --- | --- | --- | --- | --- | --- | --- | --- | --- | --- | --- |
| Case index | Ultrasound characteristics | Isolated ultrasound | Non-isolated ultrasound | Soft markers | Structural malformations | Structural malformations | | Other abnormalities | Pregnancy outcome | Outcome follow-up |
|  |  |  |  |  |  | Cardiovascular system | Urinary system |  |  |  |
| 1 | Lateral ventricle broadening | Yes | / | Yes | / | / | / | / | Cesarean | Male, Full-term delivery and SGA, Physical examination shown short stature and low weight (Growth developmental delay) |
| 3 | Choroid plexus cysts | Yes | / | Yes | / | / | / | / | Cesarean | Female, Well survivor |
| 4 | Echogenic intracardiac focus | Yes | / | Yes | / | / | / | / | Eutocia | Male, Well survivor |
| 6 | Dilated renal pelvis | Yes | / | Yes | / | / | / | / | Eutocia | Male, Well survivor |
| 8 | Dilated renal pelvis | Yes | / | Yes | / | / | / | / | Cesarean | Male, Well survivor |
| 11 | Choroid plexus cysts | Yes | / | Yes | / | / | / | / | TP | / |
| 12 | Pulmonary stenosis, Mild tricuspid regurgitation | / | Yes | / | Yes | Yes | / | / | Eutocia | Male, Preterm delivery and AGA, Mild pulmonary stenosis, Patent ductus arteriosus, Mild tricuspid regurgitation, Inguinal hernia, Physical examination shown short stature and low weight (Growth developmental delay) |
| 16 | Subependymal cysts | Yes | / | / | / | / | / | Yes | Eutocia | Male, Well survivor |
| 17 | Dilated renal pelvis, Lateral ventricle broadening | / | Yes | Yes | / | / | / | / | Eutocia | Female, Well survivor |
| 19 | Left renal agenesis, Left ectopic kidney with complete dilation of left ureter | / | Yes | / | Yes | / | Yes | / | Cesarean | Male, Well survivor |
| 20 | Choroid plexus cysts | Yes | / | Yes | / | / | / | / | TP | / |
| 21 | Choroid plexus cysts | Yes | / | Yes | / | / | / | / | Eutocia | Male, Well survivor |
| 22 | Thickened nuchal translucency | Yes | / | Yes | / | / | / | / | TP | Fetus with severe β-thalassemia |
| 23 | Fast heart rhythm | Yes | / | / | / | / | / | Yes | TP | / |
| 24 | Single umbilical artery | Yes | / | Yes | / | / | / | / | Cesarean | Male, Well survivor |
| 26 | Left upper abdominal mixed mass | Yes | / | / | / | / | / | Yes | Cesarean | Male, Full-term delivery and AGA, Conjunctivitis, Bilateral testicular hydrocele, Retroperitoneal mature cystic teratoma, Mild anemia, Intestinal adhesions |
| 29 | Echogenic intracardiac focus, Single umbilical artery, Mild pulmonary regurgitation, Mild pulmonary stenosis | / | Yes | Yes | Yes | Yes | / | / | TP | / |
| 31 | Echogenic intracardiac focus | Yes | / | Yes | / | / | / | / | Eutocia | Male, Full-term delivery and AGA, Congenital heart disease, Second foramen atrial septal defect (type II), Venous sinus defect, Coronary sinus defect, Patent foramen ovale (2.5mm), Patent ductus arteriosus (2.0mm), Left eye nasolacrimal duct obstruction, Bilateral testicular hydrocele, Acute pharyngitis, Bronchitis |
| 33 | Choroid plexus cysts | Yes | / | Yes | / | / | / | / | Cesarean | Female, Well survivor |
| 34 | Permanent left superior vena cava | Yes | / | Yes | / | / | / | / | Cesarean | Female, Well survivor |
| 36 | Short femur length | Yes | / | Yes | / | / | / | / | Eutocia | Female, Full-term delivery and AGA, Language developmental delay at 1 year, Scattered erythema and wheals on both lower limbs and urticaria at 2 years |
| 38 | Choroid plexus cysts | Yes | / | Yes | / | / | / | / | Eutocia | Female, Well survivor |
| 39 | Thickened placenta | Yes | / | / | / | / | / | Yes | Eutocia | Female, Well survivor |
| 40 | Echogenic intracardiac focus | Yes | / | Yes | / | / | / | / | TP | / |
| 41 | Dilated renal pelvis | Yes | / | Yes | / | / | / | / | Eutocia | Male, Full-term delivery and AGA, Torticollis, Eczema, Skin rash, Dryness and itching in face (dermatitis) at 5 months |
| 46 | Small-sized kidneys, FGR and IUGR | / | Yes | / | Yes | / | Yes | Yes | Eutocia | Female, Well survivor |
| 47 | Cavum septum pellucidum not displayed, Colon dilatation | / | Yes | Yes | / | / | / | Yes | Eutocia | Female, Well survivor |
| 48 | Ventricular septal defect, pulmonary stenosis, aortic ride across (Tetralogy of Fallot) | / | Yes | / | Yes | Yes | / | / | TP | / |
| 49 | Permanent left superior vena cava | Yes | / | Yes | / | / | / | / | Eutocia | Female, Well survivor |
| 50 | Thickened nuchal translucency | Yes | / | Yes | / | / | / | / | Eutocia | Female, Well survivor |
| 60 | Polyhydramnios, Dilated renal pelvis | / | Yes | Yes | / | / | / | Yes | Cesarean | Female, Well survivor, Fetus with paternal 11p15.3 duplication |
| FGR, fetal growth restriction; IUGR, intrauterine growth retardation; TP, termination of pregnancy; SGA, small for gestational age; AGA, appropriate for gestational age; Well survivor, the level of growth and development in line with peers; These 6 fetuses with abnormal postnatal phenotypes in outcome follow-up are highlighted in pink. Case 60 with additional genomic abnormalities is depicted in green. Soft markers: Choroid plexus cysts, Echogenic intracardiac focus, Dilated renal pelvis, Thickened nuchal translucency, Permanent left superior vena cava, Lateral ventricle broadening, Single umbilical artery, Short femur length, Cavum septum pellucidum not displayed. Structural malformations: Cardiovascular system (Pulmonary stenosis, Tricuspid regurgitation, Pulmonary regurgitation, Ventricular septal defect, Aortic ride across), Urinary system (Renal agenesis, Ectopic kidney with complete dilation of left ureter, Small-sized kidneys). Other ultrasound abnormalities: Subependymal cysts, Fast heart rhythm, Left upper abdominal mixed mass, Thickened placenta, Colon dilatation, FGR, Abnormal amniotic fluid volume (Polyhydramnios). | | | | | | | | | | |
|  |  |  |  |  |  |  |  |  |  |  |
|  |  |  |  |  |  |  |  |  |  |  |
|  |  |  |  |  |  |  |  |  |  |  |
|  |  |  |  |  |  |  |  |  |  |  |
|  |  |  |  |  |  |  |  |  |  |  |

| Table S4. Ultrasound characteristics and outcome follow-up of 58 fetuses with 16p13.11 duplication. | | | | | | | | | | | | | | |
| --- | --- | --- | --- | --- | --- | --- | --- | --- | --- | --- | --- | --- | --- | --- |
| Case index | Ultrasound characteristics | Isolated ultrasound | Non-isolated ultrasound | Soft markers | Structural malformations | Structural malformations | | | | | | Other abnormalities | Pregnancy outcome | Outcome follow-up |
|  |  |  |  |  |  | Brain | Cardiovascular system | Urinary system | Cleft lip and palate | Syndactyly | Nuchal cystic hygroma and Fetal hydrops |  |  |  |
| 61 | Cleft lip and palate | Yes | / | / | Yes | / | / | / | Yes | / | / | / | TP | / |
| 64 | Oligohydramnios, Hyperechogenic bowel, Small magenblase, Thickened placenta | / | Yes | Yes | / | / | / | / | / | / | / | Yes | Cesarean | Female, Well survivor |
| 66 | Fetal arhythmia | Yes | / | / | / | / | / | / | / | / | / | Yes | Cesarean | Female, Full-term delivery and AGA, Congenital heart disease, Atrioventricular block, Patent ductus arteriosus |
| 67 | Thickened nuchal translucency | Yes | / | Yes | / | / | / | / | / | / | / | / | Cesarean | Female, Well survivor |
| 68 | Large head circumference | Yes | / | / | / | / | / | / | / | / | / | Yes | Cesarean | Male, Well survivor |
| 69 | Liquid dark area in the right upper fetal bladder | Yes | / | / | / | / | / | / | / | / | / | Yes | Eutocia | Female, Full-term delivery and LGA, Patent foramen ovale, Mild tricuspid regurgitation, A cystic teratoma about 4×3cm in right lower abdomen |
| 71 | Oligohydramnios, FGR | / | Yes | / | / | / | / | / | / | / | / | Yes | TP | / |
| 75 | Abnormal echo in the liver, Enlarged cisterna magnal | / | Yes | Yes | / | / | / | / | / | / | / | Yes | Eutocia | Male, Well survivor |
| 76 | Oligohydramnios | Yes | / | / | / | / | / | / | / | / | / | Yes | Eutocia | Male, Preterm delivery and SGA, Low birth weight infants (1500-2499g), Neonatal necrotizing enterocolitis, Patent foramen ovale (2.0mm), Jaundice, No abnormalities in the nervous system and ophthalmological evaluation, Depigmented nevus and nevus flammeus in right lateral thigh, Diffused pinpoint sized skin rash in all over the body (dermatitis), Angular cheilitis |
| 81 | Echogenic intracardiac focus | Yes | / | Yes | / | / | / | / | / | / | / | / | Cesarean | Female, Well survivor |
| 83 | Choroid plexus cysts | Yes | / | Yes | / | / | / | / | / | / | / | / | TP | / |
| 85 | Absent or hypoplastic nasal bone | Yes | / | Yes | / | / | / | / | / | / | / | / | Eutocia | Female, Well survivor |
| 87 | FGR | Yes | / | / | / | / | / | / | / | / | / | Yes | Cesarean | Male, Well survivor |
| 88 | Single umbilical artery | Yes | / | Yes | / | / | / | / | / | / | / | / | Eutocia | Male, Well survivor |
| 89 | Dilated renal pelvis | Yes | / | Yes | / | / | / | / | / | / | / | / | TP | / |
| 93 | Absent or hypoplastic nasal bone | Yes | / | Yes | / | / | / | / | / | / | / | / | TP | / |
| 95 | Right polycystic kidney | Yes | / | / | Yes | / | / | Yes | / | / | / | / | TP | / |
| 98 | Absent or hypoplastic nasal bone | Yes | / | Yes | / | / | / | / | / | / | / | / | TP | / |
| 101 | Echogenic intracardiac focus | Yes | / | Yes | / | / | / | / | / | / | / | / | Cesarean | Male, Well survivor |
| 105 | Hydronephrosis | Yes | / | Yes | / | / | / | / | / | / | / | / | Cesarean | Male, Full-term delivery and AGA, A red skin pigmentation about 3×2cm on the forehead of the newborn, Torticollis at 4 months |
| 107 | Widened inferior vena cava | Yes | / | / | / | / | / | / | / | / | / | Yes | Cesarean | Male, Well survivor |
| 112 | Hyperechogenic bowel | Yes | / | Yes | / | / | / | / | / | / | / | / | TP | / |
| 115 | Arachnoid cyst | Yes | / | / | / | / | / | / | / | / | / | Yes | TP | / |
| 117 | Widened inner diameter of rectum | Yes | / | / | / | / | / | / | / | / | / | Yes | TP | / |
| 122 | Thickened placenta | Yes | / | / | / | / | / | / | / | / | / | Yes | Eutocia | Female, Well survivor |
| 123 | Choroid plexus cysts | Yes | / | Yes | / | / | / | / | / | / | / | / | Eutocia | Male, Preterm delivery and AGA, Neonatal necrotizing enterocolitis, Neonatal hyperbilirubinemia, Abnormal coagulation function, Patent foramen ovale (2mm), Immature retina, Sinus rhythm, Occasional premature beat, Skin rash and itching all over the body (dermatitis), Bronchitis, Myopia (0.6 score) at 3 years |
| 124 | Left renal agenesis or dysplasia | Yes | / | / | Yes | / | / | Yes | / | / | / | / | Eutocia | Male, Well survivor |
| 125 | Choroid plexus cysts, Aberrant right subclavicular artery | / | Yes | Yes | / | / | / | / | / | / | / | / | Cesarean | Male, Well survivor |
| 129 | Echogenic intracardiac focus, Small cavum septum pellucidum | / | Yes | Yes | / | / | / | / | / | / | / | / | Eutocia | Female, Well survivor |
| 133 | Short femur and humerus length | Yes | / | Yes | / | / | / | / | / | / | / | / | Eutocia | Male, Well survivor |
| 134 | Fetal left mandible subcutaneous fluid mass | Yes | / | / | / | / | / | / | / | / | / | Yes | Cesarean | Male, Full-term delivery and AGA, A 3×2cm left mandibular lymphangioma, Skin rash and drying all over the body, Atopic dermatitis |
| 136 | Aberrant right subclavicular artery | Yes | / | Yes | / | / | / | / | / | / | / | / | Eutocia | Female, Full-term delivery and AGA, Bilateral eye subconjunctival hemorrhage, Congenital heart disease, Second foramen atrial septal defect (type II), Venous sinus defect, Coronary sinus defect, Patent foramen ovale (2mm), Patent ductus arteriosus (1mm) |
| 139 | Thickened nuchal translucency | Yes | / | Yes | / | / | / | / | / | / | / | / | Cesarean | Female, Well survivor |
| 140 | Oligohydramnios, Shallow and flat parietal occipital sulcus, Small cavum septum pellucidum | / | Yes | Yes | / | / | / | / | / | / | / | Yes | Cesarean | Female, Well survivor |
| 144 | Thickened pulmonary valve, Increased anterior blood flow velocity of pulmonary valve, Mild pulmonary regurgitation, Mild tricuspid regurgitation | / | Yes | / | Yes | / | Yes | / | / | / | / | / | Eutocia | Male, Full-term delivery and AGA, Neonatal pathological jaundice, Left eye nasolacrimal duct obstruction, Increased anterior blood flow velocity of pulmonary valve, Mild pulmonary regurgitation, Mild tricuspid regurgitation, Respiratory infection |
| 147 | Ventriculomegaly | Yes | / | Yes | / | / | / | / | / | / | / | / | Eutocia | Male, Well survivor |
| 148 | Hydronephrosis, Dilated renal pelvis | / | Yes | Yes | / | / | / | / | / | / | / | / | Eutocia | Male, Well survivor |
| 149 | Aberrant right subclavicular artery | Yes | / | Yes | / | / | / | / | / | / | / | / | Cesarean | Male, Well survivor |
| 152 | Left renal cyst | Yes | / | / | Yes | / | / | Yes | / | / | / | / | Cesarean | Male, Well survivor |
| 153 | Choroid plexus cysts | Yes | / | Yes | / | / | / | / | / | / | / | / | Cesarean | Male, Well survivor |
| 154 | Oligohydramnios | Yes | / | / | / | / | / | / | / | / | / | Yes | Eutocia | Male, Well survivor |
| 155 | Abnormal echo in the liver, Hyperechogenic bowel | / | Yes | Yes | / | / | / | / | / | / | / | Yes | TP | / |
| 156 | Subependymal cysts, Absent or hypoplastic nasal bone, Widened cavum septum pellucidum | / | Yes | Yes | / | / | / | / | / | / | / | Yes | Cesarean | Male, Well survivor |
| 160 | FGR, Choroid plexus cysts, Hyperechogenic bowel | / | Yes | Yes | / | / | / | / | / | / | / | Yes | Eutocia | Female, Full-term delivery and SGA, Low birth weight infants (1500-2499g), Neonatal hypoglycemia and pneumonia, Patent ductus arteriosus (1.5mm), Patent foramen ovale (3mm), Aortic stenosis (3.1mm), Physical examination shown short stature and low weight (Growth developmental delay) |
| 163 | Thickened nuchal translucency | Yes | / | Yes | / | / | / | / | / | / | / | / | Eutocia | Male, Well survivor |
| 165 | Small head circumference | Yes | / | / | / | / | / | / | / | / | / | Yes | Cesarean | Female, Well survivor |
| 166 | Complex cardiac malformation, Complete endocardial cushion defect, Right ventricular double outlet | / | Yes | / | Yes | / | Yes | / | / | / | / | / | TP | / |
| 169 | Thickened nuchal translucency, Syndactyly | / | Yes | Yes | Yes | / | / | / | / | Yes | / | / | TP | / |
| 171 | Thickened nuchal translucency | Yes | / | Yes | / | / | / | / | / | / | / | / | TP | Fetus with severe α-thalassemia, Bart's syndrome |
| 174 | Choroid plexus cysts, Thickened nuchal translucency | / | Yes | Yes | / | / | / | / | / | / | / | / | Cesarean | Male, Well survivor |
| 181 | Hyperechogenic bowel | Yes | / | Yes | / | / | / | / | / | / | / | / | Eutocia | Female, Well survivor |
| 182 | Choroid plexus cysts | Yes | / | Yes | / | / | / | / | / | / | / | / | Cesarean | Female, Well survivor |
| 186 | Thickened nuchal translucency | Yes | / | Yes | / | / | / | / | / | / | / | / | Eutocia | Female, Well survivor |
| 190 | Persistent right umbilical vein | Yes | / | Yes | / | / | / | / | / | / | / | / | Eutocia | Male, Well survivor |
| 195 | Oligohydramnios | Yes | / | / | / | / | / | / | / | / | / | Yes | TP | Fetus with maternal Xp22.31 deletion |
| 197 | Nuchal cystic hygroma, Fetal hydrops | / | Yes | / | Yes | / | / | / | / | / | Yes | / | TP | Fetus with XO |
| 199 | Cerebellar hypoplasia, Absent or hypoplastic nasal bone, Single umbilical artery | / | Yes | Yes | Yes | Yes | / | / | / | / | / | / | TP | Fetus with abnormal chromosome X |
| 201 | Thickened nuchal translucency | Yes | / | Yes | / | / | / | / | / | / | / | / | TP | Fetus with T21 |
| FGR, fetal growth restriction; TP, termination of pregnancy; SGA, small for gestational age; AGA, appropriate for gestational age; LGA, large for gestational age; Well survivor, the level of growth and development in line with peers; These 8 fetuses with abnormal postnatal phenotypes in outcome follow-up are highlighted in blue. These 4 fetuses with additional genomic abnormalities are depicted in green. Soft markers: Thickened nuchal translucency, Choroid plexus cysts, Absent or hypoplastic nasal bone, Echogenic intracardiac focus, Aberrant right subclavicular artery, Hyperechogenic bowel, Dilated renal pelvis, Hydronephrosis, Persistent right umbilical vein, Single umbilical artery, Short femur and humerus length, Ventriculomegaly, Widened cavum septum pellucidum, Small cavum septum pellucidum, Enlarged cisterna magnal. Structural malformations: Brain (Cerebellar hypoplasia), Cardiovascular system (Complex cardiac malformation, Complete endocardial cushion defect, Right ventricular double outlet, Thickened pulmonary valve, Increased anterior blood flow velocity of pulmonary valve, Pulmonary regurgitation, Tricuspid regurgitation), Urinary system (Polycystic kidney, Renal agenesis or dysplasia, Renal cyst), Cleft lip and palate, Syndactyly, Nuchal cystic hygroma, Fetal hydrops. Other ultrasound abnormalities: Arachnoid cyst, Fetal arhythmia, Liquid dark area in the right upper fetal bladder, Fetal left mandible subcutaneous fluid mass, Large head circumference, Small head circumference, Thickened placenta, Widened inferior vena cava, Widened inner diameter of rectum, Small magenblase, Abnormal echo in the liver, Subependymal cysts, FGR, Abnormal amniotic fluid volume (Oligohydramnios). | | | | | | | | | | | | | | |
|  |  |  |  |  |  |  |  |  |  |  |  |  |  |  |
|  |  |  |  |  |  |  |  |  |  |  |  |  |  |  |
|  |  |  |  |  |  |  |  |  |  |  |  |  |  |  |
|  |  |  |  |  |  |  |  |  |  |  |  |  |  |  |
|  |  |  |  |  |  |  |  |  |  |  |  |  |  |  |
|  |  |  |  |  |  |  |  |  |  |  |  |  |  |  |
|  |  |  |  |  |  |  |  |  |  |  |  |  |  |  |
|  |  |  |  |  |  |  |  |  |  |  |  |  |  |  |

| Table S5. CMA and clinical phenotypes of 91 peripheral blood samples with 16p13.11 deletion and duplication. | | | | | | | |
| --- | --- | --- | --- | --- | --- | --- | --- |
| Case index | Gender | Age | CMA | OMIM gene | Size (Mb) | Interval | Clinical phenotypes |
| 202 | Male | 34 years | arr[GRCh37] 16p13.11(15493046-16303388)×1 | 7 | 0.81 | II | Pedigree verifcation (Case 1) |
| 203 | Female | 22 years | arr[GRCh37] 16p13.12p13.11(14760734-16303388)×1 | 14 | 1.54 | I+II | Pedigree verifcation (Case 3) |
| 204 | Female | 32 years | arr[GRCh37] 16p13.12 p13.11(14760734-16308351)×1 | 14 | 1.55 | I+II | Pedigree verifcation (Case 4) |
| 205 | Male | 31 years | arr[GRCh37] 16p13.11(15052746-16289532)×1 | 10 | 1.24 | I+II | Pedigree verifcation (Case 6) |
| 206 | Female | 29 years | arr[GRCh37] 16p13.11(15052746-16303388)×1 | 10 | 1.25 | I+II | Pedigree verifcation (Case 18) |
| 207 | Female | 30 years | arr[GRCh37] 16p13.11(15052746_16303388)×1 | 10 | 1.25 | I+II | Pedigree verifcation (Case 28) |
| 208 | Male | 39 years | arr[GRCh37] 16p13.11(15052746_16303388)×1 | 10 | 1.25 | I+II | Pedigree verifcation (Case 31) |
| 209 | Female | 28 years | arr[GRCh37] 16p13.11(15140211_16725352)×1 | 11 | 1.59 | I+II | Pedigree verifcation (Case 33) |
| 210 | Female | 29 years | arr[GRCh37] 16p13.12p13.11(14760734_16786098)×1 | 15 | 2.03 | I+II | Pedigree verifcation (Case 35 and 42), pregnancy related hyperthyroidism |
| 211 | Male | 24 years | arr[GRCh37] 16p13.11(15052746_16303388)×1 | 10 | 1.25 | I+II | Pedigree verifcation (Case 36) |
| 212 | Female | 35 years | arr[GRCh37] 16p13.11p12.3(15493046_18156351)×1 | 9 | 2.66 | II+III | Pedigree verifcation for 16p13.11p12.3 del |
| 213 | Female | 34 years | arr[GRCh37] 16p13.11(15052746_16289532)×1 | 10 | 1.24 | I+II | Pedigree verifcation (Case 58) |
| 214 | Female | 29 years | arr[GRCh37] 16p13.11p12.3(15493046_18886254)×1 | 13 | 3.39 | II+III | Pedigree verifcation (Case 43), adverse pregnancy history (G3P3, a girl with DD and MDD at 6 months), hypothyroidism, hashimoto's thyroiditis |
| 215 | Male | 6 months | arr[GRCh37] 16p13.11p12.3(15489743_18542468)×1 | 10 | 3.05 | II+III | Born in 2018/04/23, W=3650g, L=52cm, full-term delivery and AGA, eutocia, G38w, neonatal pathological jaundice and motor developmental delay, hyperspasmia and epilepsy for 3 months and treatment with Levetiracetam, MRI of head showed widening of bilateral temporal lobe brain spaces, clinical diagnosis for epilepsy, myelin developmental delay and MDD at 6 months, DD at 7 months, right eye horizontal tremor (nystagmus) at 1 years, stand and walk alone at 2 years |
| 216 | Male | 11 months | arr[GRCh37] 16p13.11p12.3(15489945_17451962)×1 | 9 | 1.96 | II+III | Born in 2023/02/05, W=2360g, full-term delivery and SGA, eutocia, G37w, low birth weight infants (1500-2499g), neonatal sepsis, purulent meningitis, α- thalassemia, subependymal hemorrhage, patent foramen ovale. Clinical diagnosis for bronchopneumonia, hypoxemia, sepsis, gastrointestinal dysfunction, moderate anemia, electrolyte imbalance, immune dysfunction, bacterial conjunctivitis, Hashimoto's thyroiditis, GDD, ascites, pleural effusion, acute sinusitis, connective tissue disease (undifferentiated type) at 10 months, scattered red papules can be seen on the trunk and lower limbs, autoimmune antibody positivity, abnormality of the immune system, systemic lupus erythematosus with corresponding antibody positivity, Resist β2-Glycoprotein Ⅰ Antibody (aβ2-GP1) (IgM=4.90 U/mL, IgG=77.15 U/mL↑), anti cardiolipin antibody (ACA) (IgG=164.20 GPL/mL↑, IgM<3 MPL/mL), mllineutrophil cytoplasmic antibodies-cANCA(+), pANCA(-) at 11 months, Parents both have the history of thalassemia, his father has hyperthyroidism and his mother has urticaria, photodermatitis, and dermatomyositis. In the past year, she has facial erythema, hair loss, joint stiffness, limb swelling, abnormal nails, and weight loss of 25kg. |
| 217 | Female | 22 years | arr[GRCh37] 16p13.11p12.3(15377182_18240388)×1 | 9 | 2.86 | II+III | Pedigree verifcation (Case 46), born in 2000/07/16, facial white spots pigmentation and tinea versicolor |
| 218 | Male | 40 years | arr[GRCh37] 16p13.11p12.3(15405945_18570517)×1 | 10 | 3.16 | II+III | Pedigree verifcation (Case 53) |
| 219 | Female | 39 years | arr[GRCh37] 16p13.12p13.11(14760734_16688008)×1 | 15 | 1.93 | I+II | Pedigree verifcation (Case 49) |
| 220 | Female | 24 years | arr[GRCh37] 16p13.11(15049264_16620944)×1 | 11 | 1.57 | I+II | Health checkup, female infertility |
| 221 | Female | 73 years | arr[GRCh37] 16p13.11(15052746_16768780)×1 | 11 | 1.72 | I+II | Health checkup |
| 222 | Male | 28 years | arr[GRCh37] 16p13.11(15052746_16726571)×1 | 11 | 1.67 | I+II | Pedigree verifcation (Case 55) |
| 223 | Male | 32 years | arr[GRCh37] 11p15.3p15.2(10963705_12728651)×3, 16p13.11(15150833_16303388)×1 | 9 | 1.15 | I+II | Pedigree verifcation (Case 60), callosity and eczema |
| 224 | Female | 4 months | arr[GRCh37] 16p13.11(15052746-16633361)×3 | 11 | 1.58 | I+II | Born in 2015/03/31, severe pneumonia, respiratory failure, liver function impairment, acute respiratory distress syndrome |
| 225 | Male | 10 years | arr[GRCh37] 16p13.11(15132264-16276117)×3 | 10 | 1.15 | I+II | Idiopathic short stature, denovo |
| 226 | Male | 38 years | arr[GRCh37] 16p13.11(15052746-16308351)×3 | 10 | 1.26 | I+II | Pedigree verifcation (Case 65) |
| 227 | Male | 2 years | arr[GRCh37] 16p13.11(15126890-16308351)×3 | 10 | 1.18 | I+II | ADHD, phimosis, nasosinusitis |
| 228 | Female | 2 years | arr[GRCh37] 16p13.11(15052746-16308351)×3 | 10 | 1.26 | I+II | Born in 2014/07/12, DD at 2 years, mental retardation, repeated respiratory infection and a mass about 2cm on the right hallux toe at 5 years, size of the mass has increased compared to before at 8 years but no pain |
| 229 | Male | 30 years | arr[GRCh37] 16p13.11(15052746-16308351)×3 | 10 | 1.26 | I+II | Health checkup, chromosome karyotype (46,XY,t(11;22)(q25;q13)) |
| 230 | Female | 26 years | arr[GRCh37] 16p13.11(15052746-16270904)×3 | 10 | 1.22 | I+II | Pedigree verifcation (Case 73) |
| 231 | Male | 39 years | arr[GRCh37] 16p13.11(15052746-16682080)×3 | 11 | 1.63 | I+II | Pedigree verifcation (Case 74) |
| 232 | Male | 28 years | arr[GRCh37] 16p13.11(15129970-16308351)×3 | 10 | 1.18 | I+II | Pedigree verifcation (Case 76) |
| 233 | Male | 25 years | arr[GRCh37] 16p13.11(15239631-16279283)×3 | 7 | 1.04 | II | Pedigree verifcation (Case 191) |
| 234 | Male | 29 years | arr[GRCh37] 16p13.11(15052746-16308351)×3 | 10 | 1.26 | I+II | Pedigree verifcation (Case 79) |
| 235 | Female | 31 years | arr[GRCh37] 16p13.11(15239631-16308351)×3 | 7 | 1.07 | II | Pedigree verifcation (Case 81) |
| 236 | Female | 26 years | arr[GRCh37] 16p13.11(15052746-16308351)×3 | 10 | 1.26 | I+II | Pedigree verifcation (Case 82) |
| 237 | Male | 30 years | arr[GRCh37] 16p13.11(15140211-16279283)×3 | 10 | 1.14 | I+II | Pedigree verifcation (Case 84) |
| 238 | Female | 26 years | arr[GRCh37] 16p13.11(15126890-16308351)×3 | 10 | 1.18 | I+II | Pedigree verifcation (Case 87) |
| 239 | Female | 28 years | arr[GRCh37] 16p13.11(15239631-16308351)×3 | 7 | 1.07 | II | Pedigree verifcation (Case 193) |
| 240 | Male | 26 years | arr[GRCh37] 16p13.11p12.3(15239631-19033992)×3 | 14 | 3.79 | II+III | Pedigree verifcation (Case 92) |
| 241 | Male | 36 years | arr[GRCh37] 16p13.11(15239631-16212104)×3 | 6 | 0.97 | II | Pedigree verifcation (Case 94) |
| 242 | Male | 31 years | arr[GRCh37] 16p13.11(15129970-16308351)×3 | 10 | 1.18 | I+II | Pedigree verifcation (Case 96) |
| 243 | Male | 32 years | arr[GRCh37] 16p13.11(15140211-16308351)×3 | 10 | 1.17 | I+II | Pedigree verifcation (Case 99) |
| 244 | Male | 36 years | arr[GRCh37] 16p13.11(15127986-16308351)×3 | 10 | 1.18 | I+II | Pedigree verifcation for 16p13.11 dup |
| 245 | Female | 32 years | arr[GRCh37] 16p13.11(15129970-16308351)×3 | 10 | 1.18 | I+II | Pedigree verifcation (Case 100) |
| 246 | Female | 34 years | arr[GRCh37] 16p13.11(15129970-16308351)×3 | 10 | 1.18 | I+II | Pedigree verifcation (Case 101) |
| 247 | Female | 31 years | arr[GRCh37] 16p13.11(15126890-16633361)×3 | 11 | 1.51 | I+II | Pedigree verifcation (Case 126), chromosome karyotype (46,XX,1qh+) |
| 248 | Female | 28 years | arr[GRCh37] 16p13.11(15052746-16195898)×3 | 9 | 1.14 | I+II | Pedigree verifcation (Case 102) |
| 249 | Male | 42 years | arr[GRCh37] 16p13.11(15493046-16308351)×3 | 7 | 0.82 | II | Pedigree verifcation (Case 103) |
| 250 | Male | 33 years | arr[GRCh37] 16p13.11(15140211-16308351)×3 | 10 | 1.17 | I+II | Pedigree verifcation (Case 104) |
| 251 | Male | 47 years | arr[GRCh37] 16p13.11(15129970-16308351)×3 | 10 | 1.18 | I+II | Pedigree verifcation (Case 105) |
| 252 | Male | 34 years | arr[GRCh37] 16p13.12p13.11(14758345-16308351)×3 | 14 | 1.55 | I+II | Pedigree verifcation (Case 106) |
| 253 | Female | 21 years | arr[GRCh37] 16p13.11p12.3(15239631-18143302)×3 | 9 | 2.9 | II+III | Pedigree verifcation (Case 107) |
| 254 | Female | 33 years | arr[GRCh37] 16p13.11(15052746-16289532)×3 | 10 | 1.24 | I+II | Pedigree verifcation (Case 111) |
| 255 | Female | 32 years | arr[GRCh37] 16p13.11(15127986-16212104)×3 | 9 | 1.08 | I+II | Pedigree verifcation (Case 112 and 160), pityrosporum folliculitis, skin rash and itching all over the body (dermatitis) |
| 256 | Male | 40 years | arr[GRCh37] 16p13.11(15127986-16308351)×3 | 10 | 1.18 | I+II | Health checkup, adverse pregnancy history (microdeletion of chromosome 17 and microduplication of chromosome 19) |
| 257 | Male | 33 years | arr[GRCh37] 16p13.11(15127986-16308351)×3 | 10 | 1.18 | I+II | Pedigree verifcation (Case 113) |
| 258 | Female | 25 years | arr[GRCh37] 16p13.11(15126890-16279283)×3 | 10 | 1.15 | I+II | Pedigree verifcation (Case 114 and 164) |
| 259 | Male | 28 years | arr[GRCh37] 16p13.11(15239631-16279283)×3 | 7 | 1.04 | II | Pedigree verifcation (Case 115) |
| 260 | Female | 32 years | arr[GRCh37] 16p13.11(15127986-16308351)×3 | 10 | 1.18 | I+II | Pedigree verifcation for 16p13.11 dup |
| 261 | Female | 26 years | arr[GRCh37] 16p13.11(15127986-16633361)×3 | 11 | 1.51 | I+II | Pedigree verifcation (Case 119) |
| 262 | Male | 30 years | arr[GRCh37] 16p13.11(15127986-16308351)×3 | 10 | 1.18 | I+II | Pedigree verifcation (Case 122) |
| 263 | Female | 32 years | arr[GRCh37] 16p13.11(15239631_16279283)×3 | 7 | 1.04 | II | Pedigree verifcation (Case 125), repeated nosebleeds for 7 months |
| 264 | Male | 39 years | arr[GRCh37] 16p13.11(15129970_16308351)×3 | 10 | 1.18 | I+II | Health checkup, adverse pregnancy history |
| 265 | Male | 29 years | arr[GRCh37] 16p13.11(15127986_16308351)×3 | 10 | 1.18 | I+II | Pedigree verifcation for 16p13.11 dup |
| 266 | Male | 28 years | arr[GRCh37] 16p13.11(15140211_16308351)×3 | 10 | 1.17 | I+II | Pedigree verifcation (Case 128) |
| 267 | Female | 18 years | arr[GRCh37] 16p13.11(15127986_16272508)×3 | 10 | 1.14 | I+II | Pedigree verifcation (Case 129) |
| 268 | Male | 40 years | arr[GRCh37] 16p13.11(15127986_16308351)×3 | 10 | 1.18 | I+II | Pedigree verifcation (Case 130) |
| 269 | Female | 28 years | arr[GRCh37] 16p13.11(15542659_16319150)×3 | 6 | 0.78 | II | Pedigree verifcation (Case 131) |
| 270 | Male | 38 years | arr[GRCh37] 16p13.11(15126890_16289532)×3 | 10 | 1.16 | I+II | Pedigree verifcation (Case 137) |
| 271 | Female | 32 years | arr[GRCh37] 16p13.11(15052746_16308351)×3 | 10 | 1.26 | I+II | Pedigree verifcation (Case 138) |
| 272 | Male | 27 years | arr[GRCh37] 16p13.11(15126890_16633361)×3 | 11 | 1.51 | I+II | Pedigree verifcation (Case 139) |
| 273 | Female | 36 years | arr[GRCh37] 16p13.11(15052746_16633361)×3 | 11 | 1.58 | I+II | Health checkup, female infertility |
| 274 | Female | 27 years | arr[GRCh37] 16p13.11(15129970_16308351)×3 | 10 | 1.18 | I+II | Pedigree verifcation (Case 142) |
| 275 | Male | 6 years | arr[GRCh37] 16p13.11p12.3(15239631_18156351)×3 | 9 | 2.92 | II+III | CMA of AF in prenatal diagnosis shownd 2.66Mb duplication in 16p13.11p12.3 region. Born in 2015/03/15, ADHD, sensory disorder for 1 year, normal intellectual and language development |
| 276 | Female | 29 years | arr[GRCh37] 16p13.11(15052746_16279283)×3 | 10 | 1.23 | I+II | Pedigree verifcation (Case 145) |
| 277 | Female | 28 years | arr[GRCh37] 16p13.11(15127986_16308351)×3 | 10 | 1.18 | I+II | Born in 1993/11/20, moderate to severe ID, normal motor development, adverse pregnancy history (G2P1, a girl with MDD) |
| 278 | Male | 30 years | arr[GRCh37] 16p13.11(15126890_16308351)×3 | 10 | 1.18 | I+II | Pedigree verifcation (Case 149), skin rash and itching all over the body (dermatitis) |
| 279 | Female | 35 years | arr[GRCh37] 16p13.11p12.3(15239631_18156351)×3 | 9 | 2.92 | II+III | Pedigree verifcation (Case 155) |
| 280 | Female | 31 years | arr[GRCh37] 16p13.11(15140211_16633361)×3 | 11 | 1.49 | I+II | Pedigree verifcation (Case 159) |
| 281 | Female | 27 years | arr[GRCh37] 16p13.11(15052746_16308351)×3 | 10 | 1.26 | I+II | Pedigree verifcation (Case 161) |
| 282 | Male | 41 years | arr[GRCh37] 16p13.11(15052746_16308351)×3 | 10 | 1.26 | I+II | Health checkup, secondary infertility, oligozoospermia |
| 283 | Male | 4 years | arr[GRCh37] 16p13.11(14805443_16441779)×3 | 13 | 1.64 | I+II | Born in 2016/12/09, DD, ID, ASD, cannot speak at 3 years and 5 months |
| 284 | Male | 10 days | arr[GRCh37] 16p13.11(14818301_16485993)×3 | 13 | 1.66 | I+II | Neonatal convulsions, epilepsy, feeding intolerance, bronchopulmonary dysplasia, congenital septum pellucidum abnormalities, central atrial septal defect (foramen ovale), bilateral cryptorchidism, myocardial zymogram abnormalities, sinusitis. DD and dystrophia at 1 month, MDD at 6 months |
| 285 | Female | 28 years | arr[GRCh37] 16p13.11p12.3(15474185_18341860)×3 | 9 | 2.87 | II+III | Pedigree verifcation (Case 163) |
| 286 | Male | 34 years | arr[GRCh37] 16p13.11(15054096_16351528)×3 | 11 | 1.3 | I+II | Pedigree verifcation (Case 175), azoospermia |
| 287 | Male | 40 years | arr[GRCh37] 16p13.11(14979007_16173004)×3 | 11 | 1.19 | I+II | Pedigree verifcation (Case 180) |
| 288 | Female | 29 years | arr[GRCh37] 16p13.11(15057763_16398315)×3 | 11 | 1.34 | I+II | Pedigree verifcation (Case 182) |
| 289 | Female | 22 years | arr[GRCh37] 16p13.11(15126890_16633361)×3 | 11 | 1.51 | I+II | Born in 2000/10/27, ID |
| 290 | Female | 25 years | arr[GRCh37] 16p13.11(15117808_16719098)×3 | 11 | 1.6 | I+II | Pedigree verifcation (Case 166) |
| 291 | Male | 27 years | arr[GRCh37] 16p13.11(15052746_16308351)×3 | 10 | 1.26 | I+II | Pedigree verifcation for 16p13.11 dup |
| 292 | Male | 29 years | arr[GRCh37] 16p13.11(15126890_16682080)×3 | 11 | 1.56 | I+II | Pedigree verifcation (Case 188) |
| DD, global developmental delay; GDD, growth developmental delay; MDD, motor developmental delay; LDD, language developmental delay; ADHD, attention-deficit hyperactivity disorder; ID, intellectual disability; ASD, autism spectrum disorder. These 14 patients with abnormal clinical phenotypes are highlighted in pink or blue. Case 223 with additional genomic abnormalities are depicted in green. | | | | | | | |
|  |  |  |  |  |  |  |  |
|  |  |  |  |  |  |  |  |
